# Hydrogen Isotope Exchange in Pyridine Catalyzed by an Iron(II) Imido Complex: Counterion‐Directed Regioselectivity

**DOI:** 10.1002/anie.4646822

**Published:** 2026-04-28

**Authors:** Bin Feng, Guorong Li, Yafei Gao, Nobuyuki Yamamoto, Maren Pink, Qian Peng, Jeremy M. Smith

**Affiliations:** ^1^ Department of Chemistry Indiana University Bloomington Indiana USA; ^2^ State Key Laboratory of Elemento‐Organic Chemistry Tianjin Key Laboratory of Biosensing and Molecular Recognition College of Chemistry Frontiers Science Center for New Organic Matter Nankai University Tianjin China; ^3^ Haihe Laboratory of Sustainable Chemical Transformations Tianjin China

**Keywords:** C─H activation, deuterium, hydrogen isotope exchange, iron imido complex

## Abstract

High‐spin (*S* = 2) iron(II) imido complexes [Ph_2_B(*^t^* BuIm)_2_Fe═NDipp]M (M = Li^+^, K^+^, K(18‐c‐6)^+^) are catalysts for the hydrogen isotope exchange (HIE) reaction with pyridine as the substrate. As dictated by the counter‐cation, these complexes catalyze site‐selective *α*‐, *α*,*β*,*γ*‐, and *β*,*γ*‐deuteration of pyridine. Experimental and computational mechanistic investigations reveal the critical role of the counter‐cation in catalysis, which activates the substrate, facilitates deuteration, and dictates HIE regioselectivity. The stoichiometric reaction of pyridine with [Ph_2_B(*^t^* BuIm)_2_Fe═NDipp]Li affords the catalytically active bis(2‐pyridyl) complex [Ph_2_B(*^t^* BuIm)_2_Fe(2‐Py)_2_Li(THF)_2_]. By maintaining coordination to the substrate during the catalytic cycle, Li^+^ preorganizes pyridine for regioselective *α*‐deuteration by this catalyst. On the other hand, [Ph_2_B(*^t^* BuIm)_2_Fe═NDipp]K reacts with pyridine to afford the 2‐pyridyl amido complex [Ph_2_B(*^t^* BuIm)_2_Fe(2‐Py)NHDipp]^−^, which has been structurally characterized with [K(18‐c‐6)(THF)_2_]^+^ and [K(dibenzo‐18‐c‐6)(THF)_2_]^+^ counterions. As dictated by the size of the counter‐cation, [Ph_2_B(*^t^* BuIm)_2_Fe═NDipp]^−^ catalyzes regioselective *α*,*β*,*γ*‐ and *β*,*γ*‐deuteration of pyridine. Here, the counter‐cation stabilizes the appropriate pyridyl regioisomer for the selectivity‐determining deuteration step.

## Graphical abstract

Anionic high‐spin iron(II) imido complexes are catalysts for the hydrogen isotope exchange (HIE) reaction with pyridine as the substrate. The alkali metal counter‐cation plays critical roles in activating the substrate and facilitating deuteration. These complexes catalyze site‐selective *α*‐, *α*,*β*,*γ*‐, and *β*,*γ*‐deuteration of pyridine, where the counter‐cation stabilizes the appropriate pyridyl regioisomer for the selectivity‐determining deuteration step.

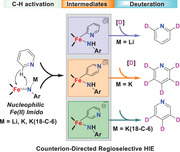

## 1 Introduction

Deuterium‐labeled organic molecules have received substantial attention in fields ranging from mechanistic elucidation to biological metabolite investigation [1, 2, 3, 4, 5, 6, 7, 8, 9]. The greater stability of C─D over C─H bonds, resulting from kinetic and equilibrium isotope effects, can alter the biological profile of a compound [10, 11, 12, 13, 14, 15, 16]. For example, this isotopic substitution may increase the metabolic stability, prolong the biological half‐life, and reduce the toxicity of a compound, resulting in an improved pharmacokinetic profile [3, 17, 18]. In addition, selectively deuterated probes allow the fate of a particular molecular site to be tracked, which can provide important information on reaction mechanisms and metabolism [6, 7, 8, 9]. The promise of selectively deuterated drug molecules is illustrated by deutetrabenazine (Austedo), which was approved by the FDA for the treatment of tardive dyskinesia caused by Huntington's disease [19, 20, 21]. The rate of drug metabolism is slowed by deuterium incorporation, allowing for less frequent dosing.

The hydrogen isotope exchange (HIE) reaction is a leading strategy for introducing deuterium at specific C─H bonds in organic compounds [4, 5]. Significant research efforts have been directed toward the development of both stoichiometric and catalytic methods for HIE, including those that are driven by electrochemical and photochemical energy sources [22, 23, 24, 25, 26, 27]. In the case of thermally driven HIE, two general catalyst design strategies have emerged. The first strategy takes advantage of the ability of certain metal complexes to effect hydrocarbon C─H activation. In general, these catalysts are selective for HIE at *sp* ^2^‐hybridized C─H bonds and are commonly applied to substrates containing arene functional groups. The second strategy uses alkali metal bases to deprotonate C─H bonds for H/D exchange. In this case, the HIE selectivity is dictated by the acidity of the C─H bond.

Despite significant advances in catalyst design, HIE in *N*‐heterocycles such as pyridine is still challenging, particularly in the absence of activating groups [28, 29, 30, 31]. Here, an alkali base catalyst was reported to be selective for HIE at the more acidic *β* and *γ* positions in pyridine [28, 30]. Since *α*‐deprotonation creates a relatively unstable anion resulting from electronic repulsion with the adjacent lone pair on the nitrogen atom, two‐step strategies are required for base‐catalyzed α‐selective HIE [31, 32]. While *α*‐selective HIE has been observed with transition metal catalysts [33, 34, 35, 36], multiple‐site deuteration may also occur [37, 38].

**FIGURE 1:**
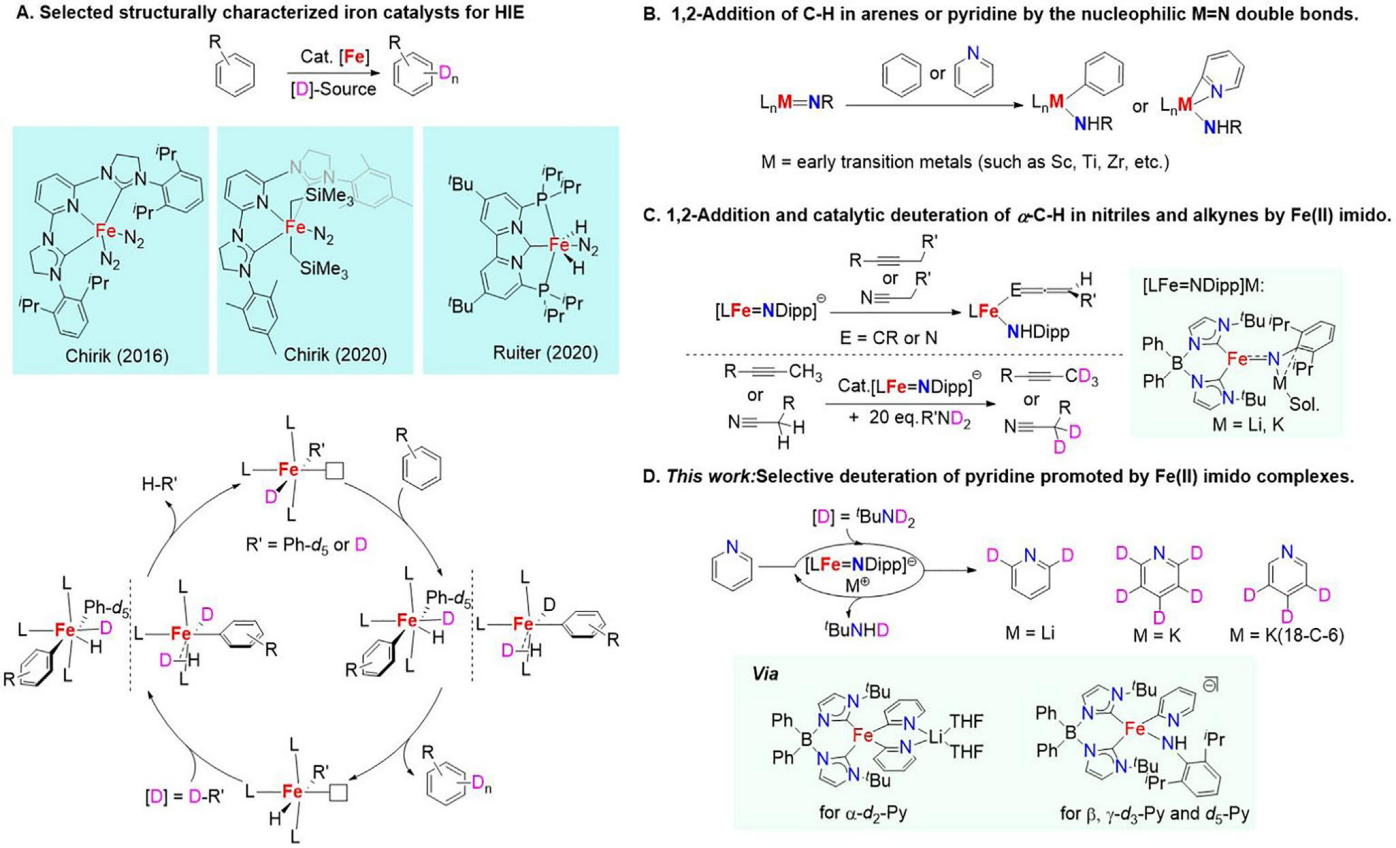
(A) Reported iron catalysts for the HIE reaction of arene and the mechanisms involved. (B) Reactivity patterns of rare‐earth metal imido complexes with arene. (C) Ene‐like reactivity of the Fe(II) imido complexes enables the catalytic *α*‐deuteration of nitriles and alkynes. (D) This work: Selective catalytic deuteration of pyridine by the cooperative action of an Fe(II) imido complex and the alkali metal ion counterions.

While historically focused on precious metal catalysts, base metal complexes for catalytic HIE have received greater attention in recent years [34, 39, 40, 41, 42, 43, 44, 45, 46, 47]. For example, the iron pincer catalysts reported by Chirik and de Ruiter groups (Figure 1A) catalyze HIE in arene‐based substrates under mild conditions. Experimental and computational studies suggest that HIE involves either a complex‐assisted *σ*‐bond metathesis pathway [41] or occurs via oxidative addition/reductive elimination steps involving a Fe(0/II/IV) cycle [48, 49, 50, 51, 52, 53, 54, 55] with iron dihydrogen complexes as key intermediates. These mechanisms share common features with those proposed for C─H activation by precious metal complexes, where the transition metal ion is the locus of HIE reactivity.

As exemplified by the reactions of hydrocarbons with early transition metal imido complexes, a complementary pathway for transition metal‐mediated C─H activation involves the 1,2‐addition of a C─H bond across a metal ligand double bond (Figure 1B) [56, 57, 58, 59, 60]. This reactivity stems from the polarized M═N double bond creating a nucleophilic imido ligand, enabling a concerted 1,2‐addition reaction that involves interactions between the imido nitrogen lone pair with the C─H σ* orbital and an empty metal d orbital with the C─H *σ* orbital [61].

Unusually for a late metal, the imido ligand in the anionic high‐spin Fe(II) complexes [Ph_2_B(*^t^* BuIm)_2_Fe = N(Dipp)]M (**1‐M, M = Li^+^, Na^+^, K^+^, K(18‐c‐6)^+^, K(crypt)^+^**), as shown in Figure 1C, is nucleophilic, with reactivity patterns akin to those of early metal imido complexes. For example, like early metal imido complexes, these complexes also undergo 1,2‐addition reactions with certain hydrocarbons to create new Fe─C and N─H bonds. In contrast to early metal imido complexes, the p*K*_a_ of the substrate C─H bond is a good reactivity predictor, suggesting the transition state for C─H activation is less concerted in **1‐M**. This 1,2‐addition reactivity forms the basis of catalytic applications, including selective HIE for the *α*‐C─H bonds of nitriles and alkynes (Figure 1C) [62, 63, 64, 65].

We previously estimated p*K*_a_ ≈ 36 in DMSO for the N─H bond of the conjugate acid of **1‐M** (Ph_2_B(*^t^* BuIm)_2_Fe─N(H)(Dipp)) [65]. Although the C─H bonds of pyridine are less acidic (computed p*K*_a_ = 41.0 – 43.6 in DMSO) [66], we anticipated that the energy gain associated with Fe─C bond formation may provide sufficient driving force for the iron imido unit to insert into a pyridine C─H. Such reactivity would provide a platform for developing catalytic HIE with pyridine as the substrate.

In this work, we report on the catalytic activity of complexes **1‐M** in regioselective HIE with pyridine by *^t^* BuND_2_ as a deuterium source [67]. While the stoichiometric reaction of [Ph_2_B(*^t^* BuIm)_2_Fe═N(Dipp)]^−^ with pyridine demonstrates the inherent reactivity of the complex toward C─H insertion, the counter‐cations are critical to enabling catalysis. Experimental and computational investigations reveal the counter‐cations electrostatically activate the pyridine substrate as well as sterically dictate the regioselectivity of HIE (Figure 1D).

## 2 Results and Discussion

### 2.1 HIE Regioselectivity is Determined by the Catalyst

We observe α‐selective HIE of pyridine with *^t^* BuND_2_ as the deuterium source in the presence of 5 mol % **1‐Li** (Table 1, entry 1). Faster conversion occurs in C_6_D_6_ than THF‐*d*_8_, suggesting an intimate role for Li^+^ in catalysis that is hindered by solvation in THF‐*d*_8_ (Table 1, entries 1 and 2). The *α*‐*d*_2_‐pyridine product was quantified by ^1^H, ^2^H and ^13^C{^1^H} NMR spectroscopies (Figures S10–S12). Catalysis is notable for the elevated level of deuteration and high regioselectivity. This regioselectivity is comparable to that observed with some transition metal catalysts [33, 35, 68].

**TABLE 1:** Catalytic site‐selective deuteration of pyridine^a^, ^b^.

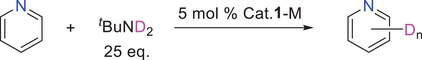					
No.	Catalyst	Temperature (^o^C)	Solvent	Time (h)	Product
1	**1**‐Li	100	THF‐*d*_8_	72	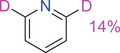
2	**1**‐Li	100	C_6_D_6_	72	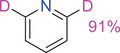
3	**1**‐K	100	THF‐*d*_8_	72	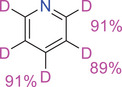
4	**1**‐K	100	C_6_D_6_	72	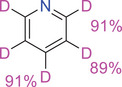
5^c^	**1‐K(18‐c‐6)**	60	THF‐*d*_8_	72	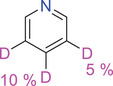
6^d^	**1‐K(18‐c‐6)**	40	THF‐*d*_8_	72	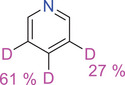
7	**1‐K(dibenzo‐18‐c‐6)**	40	THF‐*d*_8_	72	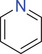
8	**1**‐K(cryptand)	40	THF‐*d*_8_	72	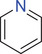

^a^ Reaction conditions: pyridine (0.7 mmol), *^t^* BuND_2_ (17.2 mmol), with 5 mol % Cat.(**1‐M**) in 0.4 mL of THF‐*d*_8_ or C_6_D_6_.

^b^ Deuterium incorporation of was determined by a combination of ^1^H and ^13^C{^1^H} NMR spectroscopies (Mesitylene as the internal standard for *d*_5_‐pyridine).

^c^ The level of deuteration approaches the final value during the first few hours of the reaction and only increases slightly with extended reaction times.

^d^ Catalyst **1‐K(18‐c‐6)** was added in 3 mol% aliquots every 24 h to reach a total of 9 mol%.

Catalytic HIE between pyridine and *^t^* BuND_2_ is also observed in the presence of **1‐K**. Interestingly, and in contrast to catalysis with **1‐Li**, HIE is observed at all positions of the pyridine substrate. In this case, the rate of catalysis is not solvent dependent (Table 1, entries 3 and 4). Similarly high levels of perdeuteration have been observed with some homogeneous and heterogeneous precious metal catalysts [37, 38].

Changing the coordination environment around K^+^ leads to further changes in regioselectivity. Specifically, when K^+^ is coordinated by 18‐crown‐6, HIE is *γ*‐selective, with smaller levels of enrichment at the β‐position. Notably, HIE is not observed at the *α*‐position of pyridine (Table 1, entries 5 and 6). To our knowledge, this regioselectivity is unique for transition metal‐catalyzed HIE, although similar to that observed with KO*^t^* Bu in DMSO‐d_6_ [28, 29]. Finally, coordinating K^+^ with the larger dibenzo‐18‐crown‐6 or sequestering it with 2,2,2‐cryptand completely shuts down catalysis (Table 1, entries 7 and 8).

Together, these observations point to an active role for the alkali–metal counter‐cation in HIE catalysts by **1‐M**. The absence of HIE catalysis for **1‐K(dibenzo‐18‐c‐6)** and **1‐K(crypt)** suggests that the counter‐cation plays an active role in the reaction. This is further evidenced by the strong dependence of regioselectivity on the nature of the counter cation. To provide insight into these observations, we conducted mechanistic investigations through a combination of synthetic and computational studies.

### 2.2 Synthetic Studies Reveal the Mode of Pyridine C─H Activation is Dictated by the Counter‐Cation

**FIGURE 2:**
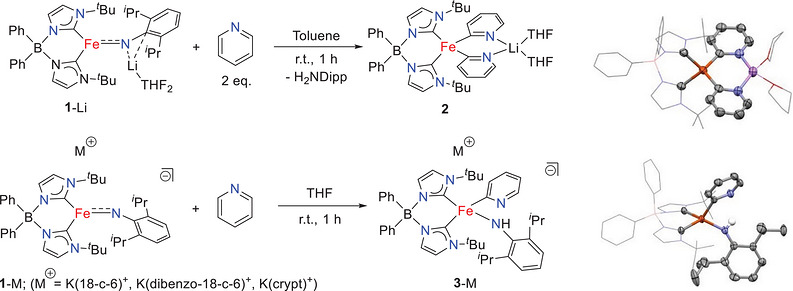
Left: Synthesis of Fe(II) complexes **2‐** and **3‐M (M = K(18‐c‐6)^+^**, **K(dibenzo‐18‐c‐6)^+^**, **K(crypt)^+^**). Right: Molecular structure of **2‐** and **3‐K(dibenzo‐18‐c‐6)**, as determined by single crystal X‐ray diffraction. Ellipsoids are shown at 50% probability level, THF and bis(carbene)borate ligand (except for carbene carbon atoms) represented as a wireframe. Most hydrogen atoms, K(dibenzo‐18‐c‐6)^+^ counterion, and solvent molecules are omitted for clarity. The dark gray, light gray, blue, pink, orange, red, and purple ellipsoids represent carbon, hydrogen, nitrogen, boron, iron, oxygen, and lithium, respectively.

Brown‐red **1‐Li** reacts with excess pyridine to afford the orange high‐spin (*S* = 2) Fe(II) complex [Ph_2_B(*^t^* BuIm)_2_Fe(2‐Py)_2_Li(THF)_2_] (**2**), which can be isolated in 71% yield (Figure 2, top). Substoichiometric quantities of pyridine result in incomplete conversion of **1**‐**Li** to **2**. 2,6‐Diisopropylaniline is identified as the other product of the reaction, as determined by GC‐MS.

The molecular structure of **2** has been determined by single‐crystal X‐ray diffraction. The most notable feature of the structure is the newly formed bis(2‐pyridyl)metalate ligand that is *N*‐coordinated to the lithium counter‐cation. The Fe─C(2‐Py) bond lengths (2.077(3) and 2.066(3) Å) are within the range observed other for Fe─C(2‐Py) bonds (2.065–2.084 Å), for example, LFe(*μ*‐4‐R_1_‐pyridin‐2‐yl)(*μ*‐R_2_)FeL (L = 4‐bis(2,6‐dimethylphenylimino)‐3‐methylpent‐3‐yl; R_1_ = H or *^t^* Bu; R_2_ = H or NCHCHPh_2_) [69]. The Li─N bond lengths (2.000(7) and 2.008(7) Å) are similar to those found in other bis(2‐pyridyl)metalate ligands, including [{Me_2_Al(6‐Me‐2‐py)_2_}{Li}] (2.052(3) and 2.068(3) Å) and [{R_2_Ga(2‐^6O^ *^t^* ^Bu^py)_2_}{Li(THF)}] (R = *^i^* Pr, 1.990 Å (avg.); R = *^t^* Bu, 1.963 Å (avg.)) [70, 71]. The metrical parameters also reveal that the pyridine rings retain their aromaticity. Together, the structural data indicate the formation of a tight ion‐pair complex between the ferrate complex [Ph_2_B(*^t^* BuIm)_2_Fe(2‐Py)_2_]^−^ and a Li^+^ cation.

Complex **2** is ^1^H NMR silent, with the Evans’ method solution magnetic moment (*µ*_eff_ = 5.0(1) *µ*_B_) consistent with a high‐spin state (*S* = 2). No intermediates are detected by ^1^H NMR spectroscopy when **1‐Li** is titrated with pyridine (Figures S7 and S8). Together with the structural data, this suggests that **2** is formed in a stepwise mechanism that involves initial proton transfer from pyridine to the basic imide ligand, and that chelation of the lithium ion drives formation of the bis(2‐pyridyl)metalate ligand in a second proton transfer reaction.

Since **1‐Li** reacts with excess pyridine to afford **2**, this suggests the latter complex is the active species for catalysis. Indeed, **2** also catalyzes HIE in pyridine with *^t^* BuND_2_ as the deuterium source, with a performance that is identical to that of **1‐Li**.

**SCHEME 1:**
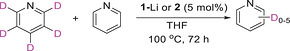
Catalytic Isotope Exchange between *d*_0_‐pyridine and *d*_5_‐pyridine by **1‐Li** or **2**.

An isotope exchange experiment between pyridine and *d*_5_‐pyridine in the presence of 5 mol% **1**‐Li or **2** leads to the formation of H/D mixed pyridines (*d*_1‐4_‐pyridines), as observed by mass spectrometry (Figure S22). This result demonstrates that formation of the 2‐pyridyl ligands in **2** is reversible (Scheme 1).

Pyridine also reacts with **1‐K(dibenzo‐18‐c‐6)**, however, in contrast to **1‐Li**, the iron imido unit inserts into the *α*‐C─H bond to afford **3‐K(dibenzo‐18‐c‐6)** (Figure 2, bottom). An analogous complex is likely the initial product of the reaction of **1‐Li** with pyridine. It is notable that **3‐K(dibenzo‐18‐c‐6)** does not react with additional pyridine, highlighting the critical role of the counter‐cation in determining the final reaction product.

The molecular structure of **3‐K(dibenzo‐18‐c‐6)**, as determined by single‐crystal X‐ray diffraction [72], reveals a four‐coordinate iron center (*τ* = 0.8) [73] that is bound to the bis(carbene)borate, 2‐pyridyl, and 2,6‐diisopropylanilido ligands. The Fe─C(2‐Py) bond length (2.088(5) Å) is similar to analogous bonds in **2** (2.077(3) and 2.066(3) Å), while the Fe─N bond length (2.018(4) Å) is similar those observed in the previously reported bis(anilido) complex [Ph_2_B(*^t^* BuIm)_2_Fe(NHDipp)_2_][K(18‐c‐6)THF_2_] (2.050(2) and 2.024(2) Å) [62]. The amido ligand proton was located in the Fourier difference map. The ^1^H NMR spectrum of **3‐K(dibenzo‐18‐c‐6)** reveals paramagnetically shifted resonances whose integrations are consistent with the solid‐state structure. The high‐spin (*S* = 2) state is determined by Evans’ solution magnetometry (*µ*_eff_ = 5.0(1) *µ*_B_). Pyridine reacts similarly with **1‐K(18‐c‐6)** and **1**‐**K(crypt)** to afford **3‐K(18‐c‐6)** and **3‐K(crypt)**, as determined by ^1^H NMR spectroscopy. Except for resonances attributed to the crown ether units, these spectra are identical (Figure S10), suggesting that the counter‐cation has little impact on the solution structure of the iron pyridyl anilido complex.

**SCHEME 2:**
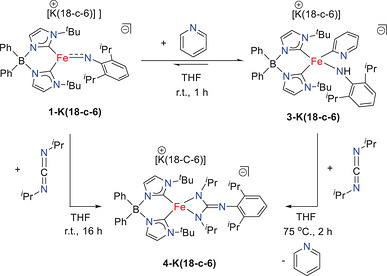
Reversible activation of the pyridine *α*‐C─H bond by **1‐K(18‐c‐6)**.

An additional experiment establishes that the formation of **3‐K(18‐c‐6)** is reversible, albeit with a sizeable barrier. Although there is no reaction at room temperature, heating **3‐K(18‐c‐6)** with equimolar *^i^* PrN═C═N*^i^* Pr results in quantitative formation of the guanidinate complex [Ph_2_B(*^t^* BuIm)_2_Fe(*^i^* PrN)_2_CNDipp]^−^ **4‐K(18‐c‐6)** [62] (Scheme 2 and Figure S13), as determined by ^1^H NMR spectroscopy. We have previously shown that **4‐K(18‐c‐6)** is formed by reaction of *^i^* PrN═C═N*^i^* Pr with the imido complex **1‐K(18‐c‐6)**, indicating that deprotonation of pyridine by the nucleophilic imido group of **1‐K(18‐c‐6)** is reversible (Scheme 2).

While these observations establish the ability of complexes **1‐M** and **2** to activate pyridine, they do not provide insight into the role of the counter‐cation in enabling catalysis or dictating the observed HIE regioselectivity. We therefore turned to density functional theory (DFT) calculations to better understand these issues.

### 2.3 Computational Investigations Establish the Critical Role of Counter Cations in HIE Catalysis

All calculations were performed at the UωB97X‐D/def2‐TZVP/SMD(solvent)//UωB97X‐D/def2‐SVP level of theory, with the solvent specified as benzene for **2** and THF for **1‐K(crypt)**, **1‐K**, and **1‐K(18‐crown‐6)**, consistent with their experimental conditions (see above, Table 1).

### 2.4 *α*‐Selective HIE by **2**

**SCHEME 3:**
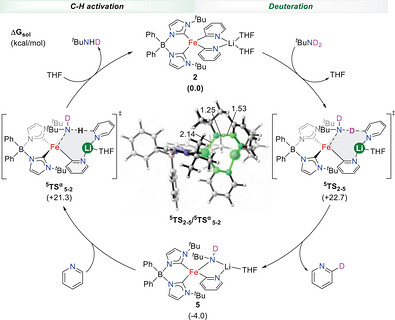
DFT‐calculated reaction mechanism for *α*‐selective HIE in pyridine with **2** as the catalyst. Relative Gibbs free energies (Δ*G_sol_*) on the quintet surface, computed with SMD(benzene) solvation, are given in parentheses. Spin state energetics were evaluated as shown in Table S6. The inset shows the optimized structure of the transition states **^5^TS_2–5_** and **^5^TS^α^_5–2_**. Bond distances are in Å.

With **2** established as the catalytically active species generated from **1‐Li**, DFT calculations were performed to elucidate the mechanism and origin of the regioselective HIE mediated by **2**, as summarized by the catalytic cycle in Scheme 3 and its full energy profile in Figure S27.

Starting from **2**, reaction with *^t^* BuND_2_ generates amido pyridyl complex **5** with the release of *α*‐deuterated pyridine. The transition state for this step (**^5^TS_2‐5_**, Δ*G* ^‡^ = 22.7 kcal/mol) results from the coordination of *^t^* BuND_2_ to Fe, concomitant with deuteration of the Fe─C bond and THF loss from Li^+^. This required solvent loss suggests that catalysis will be slower in THF than in benzene, in line with experimental observations (Table 1, entries 1 and 2).

Reaction of **5** with additional pyridine regenerates **2** through α‐C─H activation (**^5^TS** ^α^ **_5‐2_**, Δ*G* ^‡^ = 25.3 kcal/mol with respect to **5**) and closes the catalytic cycle. This α‐selective pathway is kinetically favored over the alternative *β*‐ and *γ*‐selective pathways, where the respective transition states (**^5^TS^β^_5‐2_** and **^5^TS^γ^_5‐2_**) have significantly higher barriers of 34.8 and 31.9 kcal/mol (Figure S27).

Notably, the pyridine nitrogen remains coordinated to Li^+^ throughout the catalytic cycle. This coordination enables a cooperative Li^+^/Fe bimetallic mechanism with favorable cyclic transition state geometries. Specifically, transition states, **^5^TS_2‐5_** and **^5^TS^α^_5‐2_**, are stabilized by coordination of the pyridine nitrogen to Li^+^ and the *^t^* BuND_2_ nitrogen to the Fe center (see Figure S28 for alternative transition states). Preorganization of the pyridine substrate via Li─N coordination leads to lower activation barriers for *α*‐selective HIE than for the *β*‐ and *γ*‐selective transition states, where this stabilizing coordination is absent (**^5^TS^β^_5‐2_** and **^5^TS^γ^_5‐2_**, see Figure S29 for 3D structures).

### 2.5 Pyridine Deprotonation by **1‐K(crypt)**

Our experimental results establish that the charge‐separated species **1‐K(crypt)** reacts with pyridine to afford **3‐K(crypt)** only (Figure 2), however, this complex is not active for catalytic HIE. Since the K^+^ ion is fully encapsulated by the cryptand in this complex, DFT calculations for the anionic iron(II) complex **1^−^** were used to probe the intrinsic reactivity of the iron center towards HIE catalysis, with particular emphasis on the C─H activation and deuteration steps.

**FIGURE 3:**
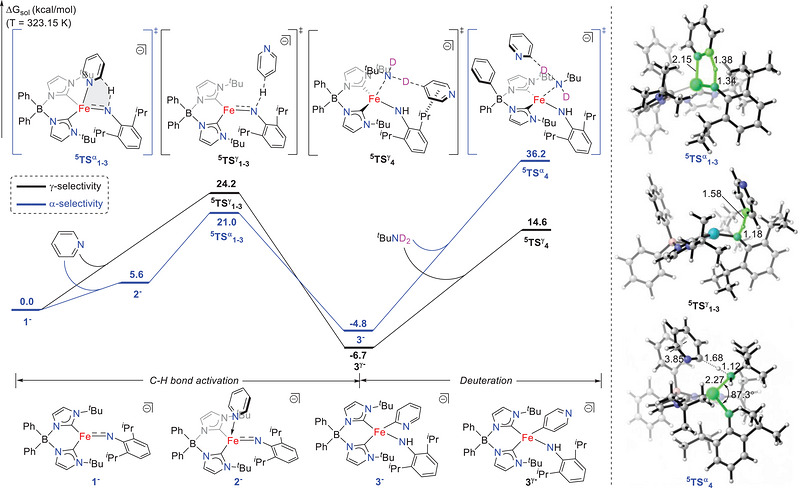
Computed Gibbs free energy profile for the deprotonation of pyridine by catalyst **1‐K(crypt)** on the quintet surface, including SMD(THF) solvation. Spin state energetics are evaluated as shown in Table S6. Bond distances are in Å.

As shown in Figure 3, the iron complex **1^−^** can deprotonate pyridine at the *α‐*position via favorable **^5^TS** ^α^ **_1‐3_** (Δ*G* ^‡^ = 21.0 kcal/mol) to yield **3^−^** (Δ*G* = −4.8 kcal/mol), which features a four‐coordinate iron center in a nearly tetrahedral geometry (*τ* = 0.88) [73]. Deprotonation at the *γ*‐position involves a higher barrier of 24.2 kcal/mol, making this pathway kinetically disfavored. The kinetic accessibility of the *α*‐position can be attributed to the cyclic transition state **^5^TS^α^_1‐3_,** unlike its counterpart for *γ*‐deprotonation (**^5^TS^γ^_1‐3_)**, which is acyclic. In the absence of an alkali metal, the coordinatively unsaturated iron center fulfills the role of a Lewis acid by coordinating the pyridine nitrogen. This coordination preorganizes the pyridine substrate for exclusive *α‐*deprotonation by bringing the *α*‐C─H bond into the proximity of the basic imido nitrogen atom. The reverse reaction to regenerate **1^−^** and pyridine via **^5^TS** ^α^ **_1‐3_** must overcome a barrier of 25.8 kcal/mol, which is beyond the thermal energy available at room temperature. These results are consistent with our experimental observation that pyridine reacts with **1‐K(crypt)^+^** to provide the α‐deprotonation product **3‐K(crypt)^+^** at room temperature (Figure 2), as well as the fact that heat is required to reverse this reaction.

Although the α‐deprotonation of pyridine by **1^−^** is kinetically favorable, the subsequent deuteration by *^t^* BuND_2_ has an insurmountable barrier (Δ*G* ^‡^ = 41.0 kcal/mol from **3^–^** to **^5^TS^α^_4_**). This high barrier arises from the acyclic transition state **^5^TS^α^_4_**, which lacks alkali–metal/Fe bimetallic cooperation. In **^5^TS^α^_4_**, the Fe center has already adopted a tetrahedral geometry (*τ* = 0.76) [73] prior to the approach of *^t^* BuND_2_. This constrained structure lacks available coordination sites required to bind both the nitrogen atoms of *^t^* BuND_2_ and pyridine. As a result, iron is only coordinated to the *^t^* BuND_2_ nitrogen, leaving the pyridine nitrogen uncoordinated. Pyridine is forced to engage in a weak π–π stacking interaction with the Ph_2_B(*^t^* BuIm)_2_^−^ ligand (see Figure S30 for the Interaction Region Indicator (IRI) analysis results). This crowded environment imposes a highly distorted geometry at the Fe center (∠N(D)─Fe─N(H) = 87.3°), resulting in a high reaction barrier. In contrast, the pyridine in **^5^TS^γ^_4_** engages in a more favorable C─H…π interaction with the NHDipp moiety (see Figure S30), which imposes less of a steric constraint. This leads to a less distorted geometry (∠N(D)‐Fe‐N(H) = 98.1°, see Figure S30) and a lower barrier (Δ*G* ^‡^ = 21.3 kcal/mol from **3^γ−^** to **^5^TS^γ^_4_**). However, since initial *α*‐deprotonation is kinetically disfavored, this pathway is inaccessible.

In summary, computational analysis of this alkali‐metal‐free system suggests that a cooperative bimetallic mechanism is required for HIE catalysis. Although the C─H activation step can be achieved by the iron center alone, its inability to mediate the deuteration step suggests an active role for the alkali metal ion in catalysis. This prompted us to investigate the role of the counter‐cation in dictating the HIE mechanism, including: (i) how the K^+^ cation fulfills an essential cooperative role and (ii) how the larger **K(18‐c‐6)^+^** cation modifies HIE regioselectivity.

### 2.6 Non‐Regioselective Catalysis by **1‐K**

**FIGURE 4:**
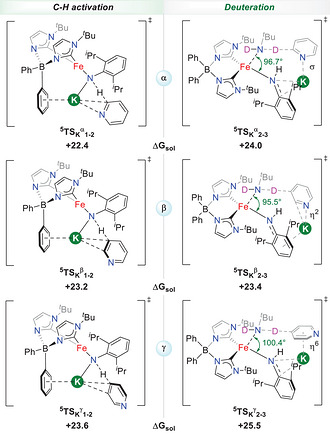
Quintet‐state transition states for *α*‐, *β*‐, and *γ*‐selective pyridine HIE with **1‐K** as the catalyst. Relative Gibbs free energies (Δ*G_sol_*) are given in kcal/mol, computed with SMD(THF) solvation.

Interestingly, while the presence of K^+^ enables catalysis at 100°C (Table 1, entry 4), the transition state energies for all three regioisomers (*α*, *β*, and *γ*) in both C─H activation and deuteration steps are comparable (Figure 4, see Figure S31 for the full energy profile). This can be attributed to the flexible coordination of K^+^ that is enabled by its large ionic radius.

In the C─H activation step, the large size of the K^+^ ion allows it to engage in a *η* ^2^‐type interaction, which enables activation at the *α*‐(via a K^+^‐*η* ^2^‐(N,C) interaction) as well as the *β*‐ and *γ*‐positions (via K^+^‐*η* ^2^‐(C,C) interactions) of pyridine (Figure 4, left). These interactions are supported by the independent gradient model based on Hirshfeld partition (IGMH) analysis (Figure S32). This leads to smaller energy barrier differences for the *α*‐, *β*‐, and *γ*‐deprotonation pathways by **1‐K** (e.g., ΔΔ*G* ^‡^ = 1.2 kcal/mol between *α*‐ and *γ*‐pathways), indicating that deprotonation proceeds without regioselectivity.

In the subsequent deuteration step, the versatility of K^+^ in engaging in cation–π interactions with the NHDipp moiety enables cyclic transition states involving a cooperative bimetallic K^+^/Fe mechanism (Figure 4, right). These cyclic transition states, **^5^TS_K_^α^_2‐3_**, **^5^TS_K_** ^β^ **_2‐3_**, and **^5^TS_K_** ^γ^ **_2‐3_**, are distinct from the acyclic transition state **^5^TS^α^_4_** of **1‐K(crypt)**. In these cyclic structures, K^+^ directs pyridine while *^t^* BuND_2_ coordinates to Fe. This allows the Fe center to adopt the optimal geometry for accommodating the incoming *^t^* BuND_2_ (e.g., ∠N(D)‐Fe‐N(H) = 96.7°, 95.5°, and 100.4°, respectively), in contrast to **^5^TS^α^_4_**, where the angle is 87.3°. Notably, although K^+^ directs pyridine through different modes ranging from *σ*‐ to *η* ^6^‐interactions (see Figure S33 for 3D structures and IGMH analysis results), its large size enables the effective construction of cyclic transition states for deuteration at all positions. Therefore, the associated energy barriers at the *α*‐, *β*‐, and *γ*‐positions are comparable (Δ*G* ^‡^ = 24.0, 23.4, and 25.5 kcal/mol for *α*‐, *β*‐, and *γ*‐positions, respectively). These barriers are accessible at the experimental temperature of 100°C, accounting for the non‐regioselective HIE observed with **1‐K** as the catalyst.

### 2.7 Regioselective Catalysis by **1‐K(18‐c‐6)**

**FIGURE 5:**
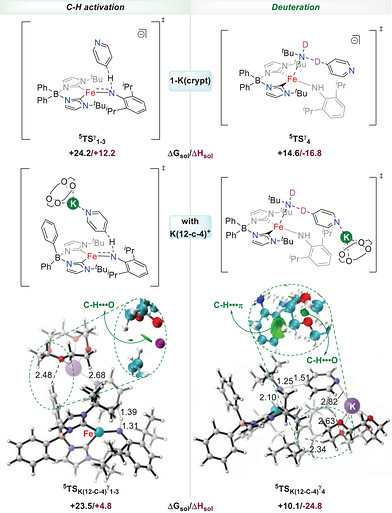
Quintet‐state transition states for *γ*‐C─H activation and *γ*‐deuteration, without and with K(12‐c‐4)^+^ coordination. Relative Gibbs free energies (black, Δ*G*_sol_) and enthalpies (dark red, Δ*H*_sol_) are given in kcal/mol with respect to the starting complex **1** ^−^, computed with SMD(THF) solvation. Bond distances are in Å.

To elucidate the role of 18‐crown‐6 in achieving regioselectivity, the transition states for the dominant *γ*‐selective pathway (**^5^TS_K(12‐C‐4)_^γ^_1‐3_** and **^5^TS_K(12‐C‐4)_^γ^_4_**) were calculated using 12‐crown‐4 as a computationally tractable surrogate. Figure 5 compares the relative energies (Δ*G_sol_* and Δ*H_sol_*) of the model **1‐K(12‐c‐4)** catalyst with those of **1‐K(crypt)**, thereby allowing for a reasonable estimate of the entropy penalty associated with K(12‐c‐4)^+^ coordination.

This energy analysis shows that K(12‐c‐4)^+^ provides *substantial enthalpic stabilization* to both **^5^TS^γ^_1‐3_** (ΔΔ*H* ^‡^ = −7.4 kcal/mol) and **^5^TS** ^γ^ **_4_** (ΔΔ*H* ^‡^ = −8.0 kcal/mol). This stabilization arises from electrostatic interactions between K^+^ and the pyridine nitrogen. Moreover, noncovalent interactions between the crown ether and the iron(II) complex **1^−^** are observed in an IGMH analysis (Figure 5, bottom). Specifically, **^5^TS_K(12‐C‐4)_^γ^_1‐3_** exhibits O…H─C dispersion interactions between the crown ether and the Ph_2_B(*^t^* BuIm)_2_^−^ ligand, while **^5^TS_K(12‐C‐4)_^γ^_4_** features C─H…π and O…H─C interactions between the crown ether and the NHDipp^−^ moiety. The substantial enthalpic gains more than compensate for the entropic cost of K(12‐c‐4)^+^ coordination, leading to an overall reduction in the free‐energy barriers (ΔΔ*G* ^‡^ = −0.7 and −4.5 kcal/mol for **^5^TS^γ^_1‐3_** and **^5^TS^γ^_4_**, respectively). This reveals that K(12‐c‐4)^+^ coordination favors the *γ*‐pathway relative to **1‐K(crypt)**, consistent with the experimental observation that **1‐K(18‐c‐6)** catalyzes *γ*‐selective HIE, whereas **1‐K(crypt)** is inactive. In contrast, attempts to model crown ether coordination for *α*‐deuteration were unsuccessful due to overwhelming steric effects, suggesting the intrinsic susceptibility of the α‐pathway to steric hindrance.

Based on these insights from the 12‐crown‐4 model, we propose two crucial roles for the larger K(18‐c‐6)^+^. First, K(18‐c‐6)^+^ directs the pyridine substrate toward the basic imido nitrogen through noncovalent interactions with **1^−^**, promoting *γ*‐C─H bond activation while sterically hindering access to the *α*‐C─H bond. Second, the larger 18‐crown‐6 more effectively restricts the cation–π interactions observed in **1‐K** (see above), allowing for *β*‐ and *γ*‐deuteration only. Taken together, these results lead to a unified mechanistic understanding of catalytic HIE by complexes **1‐M**. Specifically, the alkali–metal/Fe bimetallic cooperativity is modulated by the nature of the counter‐cation, with the *α*‐pathway sterically suppressed in **1‐K(18‐c‐6)** and catalysis inhibited in **1‐K(crypt)** due to the suppression of critical interactions with K^+^.

### 2.8 Catalysis by 1‐M (M = K, K(18‐c‐6))

**FIGURE 6:**
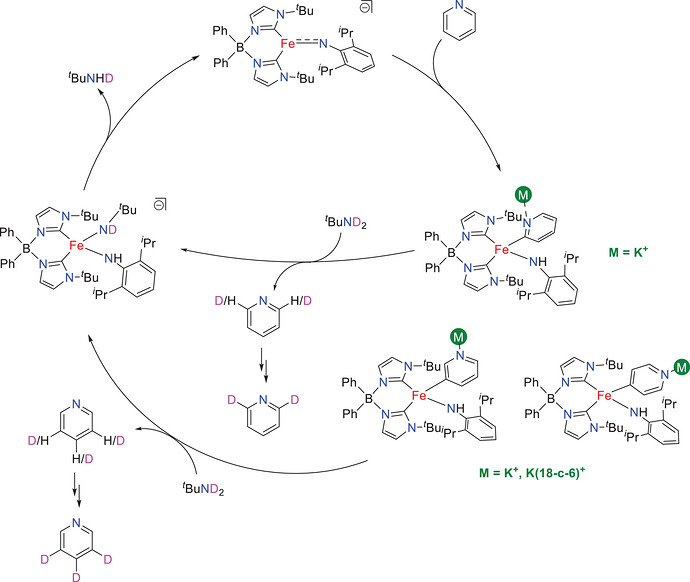
Proposed catalytic cycle for HIE in pyridine with **1‐K** and **1‐K(18‐c‐6)** catalysts based on experimental and computational mechanistic investigation.

Based on our combined experimental and computational studies, we propose a catalytic cycle shown in Figure 6. The reaction commences with pyridine deprotonation to insert the iron imido bond into a C─H bond, where counter‐cation coordination to the nitrogen atom of pyridine electrostatically activates the C─H bonds for deprotonation. This is followed by reaction with *^t^* BuND_2_ to release the deuterated pyridine and form a bis(amido) complex, with HIE regioselectivity dictated by the size of the counterion. Specifically, the large K(18‐c‐6)^+^ cation can coordinate to pyridine only when iron is bound at the *β* or *γ* positions, leading to the observed *β*‐, *γ*‐selective HIE, whereas the smaller K^+^ cation is not subject to these steric considerations, resulting in non‐regioselective HIE. In the case of **K(benzo‐18‐c‐6)** ^+^ and **K(crypt)** ^+^, the cation is unable to coordinate pyridine, preventing deuteration from occurring. Finally, amine loss regenerates the imido complex [Ph_2_B(*^t^* BuIm)_2_Fe═N(Dipp)]^−^, as previously reported [62, 64].

## 3 Conclusion

The foregoing results demonstrate the critical role of the counter cation in HIE catalysis by the Fe(II) imido complex **1** ^−^. Although the complex can activate the *α*‐C─H bond of pyridine, counter cation assistance is required for catalytic HIE. In addition to assisting substrate deprotonation through electrostatic activation of the substrate, the K^+^ and K(18‐c‐6)^+^ counter‐cations sterically dictate the regioselectivity of HIE. By contrast, with Li^+^, the imido complex is transformed to the bis(2‐pyridyl) complex **2**, where the counter‐cation kinetically favors *α*‐selective HIE by preorganizing the pyridine substrate. As discussed in the introduction, there are few examples for HIE with unfunctionalized pyridine. Moreover, transition metal and alkali base catalysts typically have divergent regioselectivities. Our work demonstrates a single transition metal catalyst with unprecedented control over HIE regioselectivity, as effected through simple modifications to the counter‐cation.

There is a growing recognition of the ability of alkali metal ions to play an active role in mediating chemical reactivity [74]. Alkali metal ions have been demonstrated to have a number of cooperative effects in the activation of C─H bonds by transition metal complexes, including modulating the activity of a basic ligand [62, 75, 76], controlling the oxidation state and coordination sphere of the metal ion [77], or modifying the equilibrium position of a reversible reaction [78]. Our results demonstrate the ability of alkali metal counter‐cations to modify the properties of the substrate, both enabling catalysis as well as dictating product regioselectivity. We anticipate that this strategy may have applicability beyond HIE catalysis in heterocycles.

## Conflicts of Interest

The authors declare no conflicts of interest.

## Supporting information



Additional experimental details, characterization data (NMR, X‐ray, UV–vis, mass spectrometry), and computational details (pdf). The authors have cited additional references within the Supporting Information. **Supporting File**: anie72343‐sup‐0001‐SuppMat.docx.

## Data Availability

The data that supports the findings of this study are available in the supplementary material of this article.
