# Supplementary material for: Hydrogen Isotope Exchange in Pyridine Catalyzed by an Iron(II) Imido Complex: Counterion‐Directed Regioselectivity
Source: Angew Chem Int Ed Engl. 2026 Apr 28;65(24):e4646822. doi: 10.1002/anie.4646822 (PMC13245597; doi:10.1002/anie.4646822)
Supplement: Supplementary file 1 — Additional experimental details, characterization data (NMR, X‐ray, UV–vis, mass spectrometry), and computational details (pdf). The authors have cited additional references within the Supporting Information. Supporting File: anie72343‐sup‐0001‐SuppMat.docx. [file ANIE-65-e4646822-s001.docx]

**Supporting Information**

**Hydrogen Isotope Exchange in Pyridine Catalyzed by an Iron(II) Imido Complex: Counterion-Directed Regioselectivity**

Bin Feng,^†^ Guorong Li,^‡^ Yafei Gao,^†^ Nobuyuki Yamamoto,^†^ Maren Pink,^†^ Qian Peng,*^,‡^ Jeremy M. Smith*^,†^

^†^ Department of Chemistry, Indiana University, 800 East Kirkwood Avenue, Bloomington, Indiana 47405, United States

^‡^ State Key Laboratory of Elemento-Organic Chemistry, Tianjin Key Laboratory of Biosensing and Molecular Recognition, College of Chemistry, Frontiers Science Center for New Organic Matter, Nankai University, Tianjin 300071, China;

Haihe Laboratory of Sustainable Chemical Transformations, Tianjin 300192, China

**Contents**

**Experimental Details, Materials and Methods** S3.

**Characterization Data of Deuterated Pyridine**........................................................................S8.

[**Supplementary Figures** S11](#_Toc44319719).

1. Molecular structures.......................................................................................................S11.

2. NMR spectra for all compounds.....................................................................................S13.

3. UV-vis spectra for **2**, **3**-K(dibenzo-18-c-6) and **3**-K(dibenzo-18-c-6)...........................S23.

[**Crystallographic Data Collection** S](#_Toc44319721)25.

**Computational Details** [S](#_Toc44319721)31.

[**References** S110](#_Toc44319722).

**Experimental Details, Materials and Methods**

**General Considerations.** All manipulations were performed under a nitrogen atmosphere by standard Schlenk techniques or in an MBraun glove box. Glassware was dried at 130 °C overnight before cooling under a dynamic vacuum in an antechamber. Diethyl ether (Et_2_O), tetrahydrofuran (THF), toluene, and pentane were purified by a PPT solvent purification system. Pyridine was degassed and stored over molecular sieves for at least one day before use. Celite was dried overnight at 130 °C under vacuum. Complexes **1-K/Li** were synthesized according to literature procedures.^1^ Deuterated solvents were purchased from Cambridge Isotope Laboratories. C_6_D_6_, C_5_D_5_N and THF-*d*_8_ were degassed and stored over molecular sieves for at least one day before use. *^t^*BuND_2_ was synthesized according to the literature procedure.^2^ All other chemicals were purchased from chemical vendors and used as received unless otherwise noted. ^1^H NMR spectroscopic data were recorded on Varian spectrometers. Solution magnetic susceptibilities were determined by Evans’ method.^3^ UV-Vis spectroscopic data were collected on an Agilent Technologies Cary 60 UV-Vis instrument. Mass spectra were recorded using negative electrospray ionization on a Thermo Electron Corp MAT-95XP spectrometer.

**Synthesis of** **[Ph_2_B(*^t^*BuIm)_2_Fe(2-Py)_2_Li] (2).** A vial was charged with [Ph_2_B(*^t^*BuIm)_2_Fe=NDipp]Li (160 mg, 0.20 mmol) and toluene (3 mL). Pyridine (64 mg, 0.80 mmol) was added at room temperature and the solution was allowed to stir for 1 h. The toluene was removed under vacuum, affording a brown-red residue that was washed with pentane (3×5 mL) to give [Ph_2_B(*^t^*BuIm)_2_Fe(**2-**Py)_2_Li] (**2**) as a brown-red powder (90 mg, 71% yield). Crystals suitable for X-ray diffraction were grown at -35 ^°^C from a concentrated THF solution that was layered with pentane. This complex is NMR silent, likely due to rapid exchange process on the 1H NMR timescale (Figures S11 and S12).a *μ*_eff_ (Evans’, C_6_D_6_, 25 ^°^C): 5.0(1) μ_B_. UV-vis (THF, 25 ^°^C): λ_max_, nm (*ε*, M^−1^cm^−1^) = 290 (17437), 320 (17139), 365 (12195). Despite multiple attempts, we have been unable to obtain satisfactory HRMS data for [Ph_2_B(*^t^*BuIm)_2_Fe(**2-**Py)_2_Li(THF)_2_] **2** was not obtained. The same situation occurred with complex [Ph_2_B(*^t^*BuIm)_2_Fe(**2-**Py)_2_MgCl(THF)_2_] (see below).

**Synthesis of [Ph_2_B(*^t^*BuIm)_2_Fe(2-Py)_2_MgCl(THF)_2_].** A vial was charged with [Ph_2_B(*^t^*BuIm)_2_Fe(*^t^*BuImH)Cl] (313 mg, 0.5 mmol) and toluene (3 mL), followed by a solution of 2-Pyridylmagnesium bromide (191 mg, 1.05 mmol) in THF (1 mL). The solution was stirred at room temperature for 4 h and then dried under vacuum. The residue was extracted with toluene, filtered through Celite and the volatiles removed under vacuum to afford a white residue that was washed with cold pentane (3 mL) to give [Ph_2_B(*^t^*BuIm)_2_Fe(**2-**Py)_2_MgCl(THF)_2_] as a yellow powder (322 mg, 74%). Crystals suitable for X-ray diffraction were grown at -35 ^°^C from a concentrated THF solution that was layered with pentane. *μ*_eff_ (Evans’, C_6_D_6_, 25 ^°^C): 4.9(1) μ_B_. ^1^H NMR (400 MHz, C_6_D_6_, 25 ^o^C): *δ* (ppm) 70.6, 49.7, 37.5, 35.4, 20.5, 18.8, 13.6, 9.9, 8.9, 4.8, 1.9, -18.4. ^1^H NMR (400 MHz, THF-*d*_8_, 25 ^o^C): *δ* (ppm) 118.4, 84.4, 53.5, 15.3, 9.8, 6.8, 4.2, -18.4.^11^B{^1^H} NMR (128 MHz, C_6_D_6_, 25 ^o^C): *δ* (ppm) 71.7. ^11^B{^1^H} NMR (128 MHz, C_6_D_6_ mixed with drops of THF-*d*_8_, 25 ^o^C): *δ* (ppm) 179.9, 70.5 (Upon comparison, this signal matches that of the complex in C_6_D_6_).

**Synthesis of [Ph_2_B(*^t^*BuIm)_2_Fe(2-Py)NHDipp][K(18-c-6)(THF)_2_] 3-K(18-c-6) and [Ph_2_B(*^t^*BuIm)_2_Fe(2-Py)NHDipp][K(dibenzo-18-c-6)(THF)_2_]** **3-K(dibenzo-18-c-6).**

A vial was charged with [Ph_2_B(*^t^*BuIm)_2_Fe=NDipp]K (136 mg, 0.20 mmol) and toluene (3 mL). Pyridine (16 mg, 0.20 mmol) and crown ether (18-crown-6 or dibenzo-18-crown-6, 0.20 mmol) were added at room temperature and the solution was allowed to stir for 1 h. The toluene was removed under vacuum, affording an orange residue that was washed with pentane (3 × 5 mL) to give [Ph_2_B(*^t^*BuIm)_2_Fe(2-Py)NHDipp][K(18-c-6)(THF)_2_] **3-K(18-c-6)** or [Ph_2_B(*^t^*BuIm)_2_Fe(2-Py)NHDipp][K(dibenzo-18-c-6)(THF)_2_] **3-K(dibenzo-18-c-6)** as an orange powder (**3-K(18-c-6**): 143 mg, 70% yield; **3-K(dibenzo-18-c-6)**: 166 mg, 74% yield). Crystals suitable for X-ray diffraction of 3-K(dibenzo-18-c-6) were grown at -35 ^°^C from a concentrated THF solution that was layered with pentane. ^1^H NMR (400 MHz, THF-*d*_8_, 25 ^o^C) of **3**-K(18-c-6): *δ* (ppm) 73.0 (1H), 37.6 (2H), 34.4 (1H), 28.9 (2H), 28.0 (1H), 26.4 (1H), 22.7 (2H), 19.3 (1H), 13. 7 (12H), 12.8 (2H), 10.5 (1H), 9.9 (2H), 7.8 (2H), 7.8 (1H), 3.6 (45H, overlapped with the residual solvent resonance of the deuterated solvent), 1.7 (4H, overlapped with the residual solvent resonance of the deuterated solvent), -53.6 (1H), -61.6 (1H); ^1^H NMR (400 MHz, THF-*d*_8_, 25 ^o^C) of **3**-K(dibenzo-18-c-6): *δ* (ppm) 73.0 (1H), 37.6 (2H), 34.4 (1H), 28.9 (2H), 28.0 (1H), 26.4 (1H), 22.7 (2H), 19.3 (1H), 13. 7 (12H), 12.8 (2H), 10.5 (1H), 9.9 (2H), 7.8 (2H), 7.8 (1H), 7.02 (4H), 6.9 (5H), 4.3 (8H), 4.1 (8H), 3.6 (45H, overlapped with the residual solvent resonance of the deuterated solvent), 1.7 (4H, overlapped with the residual solvent resonance of the deuterated solvent), -53.6 (1H), -61.6 (1H); ^1^H NMR (400 MHz, THF-*d*_8_, 25 ^o^C) of **3-K(dibenzo-18-c-6)**: *δ* (ppm) 73.0 (1H), 37.6 (2H), 34.4 (1H), 28.9 (2H), 28.0 (1H), 26.4 (1H), 22.7 (2H), 19.3 (1H), 13. 7 (12H), 12.8 (2H), 10.5 (1H), 9.9 (2H), 7.8 (2H), 7.8 (1H), 3.6 (45H, overlapped with the residual solvent resonance of the deuterated solvent), 1.7 (4H, overlapped with the residual solvent resonance of the deuterated solvent), -53.6 (1H), -61.6 (1H). The ^1^H NMR of **3-K(18-c-6)** and **3-K(dibenzo-18-c-6)** are almost the same except for counterions (see Figure S10). *μ*_eff_ (Evans’, C_6_D_6_, 25 ^°^C) of **3-K(18-c-6)**: 5.0(1) μ_B_; *μ*_eff_ (Evans’, C_6_D_6_, 25 ^°^C) of **3**-K(dibenzo-18-c-6): 5.1(1) *μ*_B_. UV-vis (THF, 25 ^°^C) of **3**-K(18-c-6): λ_max_, nm (*ε*, M^−1^cm^−1^) = 253 (25120), 289 (19425), 334 (19160), 698 (2080); UV-vis (THF, 25 ^°^C) of **3-K(dibenzo-18-c-6)**: 272 (17105), 334 (11932), 686 (1345). Despite multiple attempts, the satisfied HRMS result of [Ph_2_B(*^t^*BuIm)_2_Fe(2-Py)NHDipp][K(18-c-6)(THF)_2_] **3-K(18-c-6)** or [Ph_2_B(*^t^*BuIm)_2_Fe(2-Py)NHDipp][K(dibenzo-18-c-6)(THF)_2_] **3-K(dibenzo-18-c-6)** was not obtained. This may be because the complex is highly sensitive to water and oxygen, and even soft ionization methods such as ESI or ACPI may cause its decomposition.

**^1^H NMR Titration of 1-Li with Pyridine in C_6_D_6_.**

A J-Young tube was charged with **1-Li** (34 mg, 0.043 mmol) and C_6_D_6_ (0.4 mL). This solution was titrated with the solution was titrated with C_6_D_6_ solution (40 mg) of pyridine (0.25 eq. 0.85 mg, 0.0107 mmol). Although the reaction between **1-Li** and pyridine occurs immediately, the solution allowed to equilibrate was further at room temperature over a period of 30 min. Chemical shift was determined by ^1^H NMR spectroscopy at 25 ^o^C, demonstrating that the NMR silent complex [Ph_2_B(*^t^*BuIm)_2_Fe(2-Py)_2_Li] **2** was generated without observable intermediates (see Figure S11).

**General procedure for the catalytic deuteration of pyridine using 2 and 3-K(18-c-6) as the catalyst**.

In a typical experiment, a J-Young tube was charged with pyridine (54 mg, 0.688 mmol), *^t^*BuND_2_ (1.296 g, 17.2 mmol), **2** or **3-K(18-c-6)** (0.034 mmol, 5 mol%) and C_6_D_6_ or THF-*d*_8_ (0.4 mL). The reaction was heated at 100 °C for 72 h. The volatiles are transferred into another J-Young tube under vacuum. The deuteration ratios of pyridine were listed in Table 1.

**General procedure for the catalytic deuteration of pyridine using 3-K(dibenzo-18-c-6) as the catalyst**.

In a typical experiment, a J-Young tube was charged with pyridine (27 mg, 0.34 mmol), *^t^*BuND_2_ (128 mg, 1.7 mmol), **3-K(dibenzo-18-c-6)** (0.034 mmol, 10 mol%) and THF-*d*_8_ (0.4 mL). The reaction was heated at 40 °C for 3 days. The volatiles are transferred into another J-Young tube under vacuum. This J-Young tube was charged with **3-K(dibenzo-18-c-6)** (0.034 mmol, 10 mol%), and then the reaction was heated at 40 °C for 3 days. Repeat vacuum transfer, add catalyst, and heat at 40° for 3 days. The deuteration ratios were listed in Table 1.

**Characterization Data of Deuterated Pyridine**

^1^H NMR (500 MHz, 25 °C, C_6_D_6_): *δ* (ppm) 8.47 (d, *J* = 3.8 Hz, 0.18 H), 7.31 (t, *J* = 7.6 Hz, 1 H), 6.94 (d, *J* = 7.5 Hz, 2 H). ^2^H NMR (92 MHz, 25 °C, C_6_D_6_): *δ* (ppm) 8.46 (s). ^13^C{^1^H} NMR (151 MHz, 25 °C, C_6_D_6_): *δ* (ppm) 150.0 (dd, *J* = 52.9, 25.8 Hz), 135.5 (m), 123.6 (m). These data are in accordance with the literature.^4^

^1^H NMR (500 MHz, 25 °C, THF-*d*_8_): *δ* (ppm) 8.54 (s, 0.19 H), 7.61 (s, 0.09 H), 7.21 (s, 0.23 H). ^2^H NMR (92 MHz, 25 °C, THF-*d*_8_): *δ* (ppm) 8.54, 7.63, 7.23. ^13^C{^1^H} NMR (151 MHz, 25 °C, THF-*d*_8_) δ (ppm) 149.47(m), 135.84 (m), 122.9 (m). These data are in accordance with the literature.^5^

^1^H NMR (500 MHz, 25 °C, THF-*d*_8_): *δ* (ppm) 8.54 (s, 0.19 H), 7.61 (s, 0.09 H), 7.21 (s, 0.23 H). ^2^H NMR (92 MHz, 25 °C, THF-*d*_8_) δ (ppm) 8.54, 7.63, 7.23. ^13^C{^1^H} NMR (151 MHz, 25 °C, THF-*d*_8_): *δ* (ppm) 149.47(m), 135.84 (m), 122.9 (m). These data are in accordance with the literature.^6^

**Supplementary Figures**

**1. Molecular structures for 2, [Ph_2_B(*^t^*BuIm)_2_Fe(2-Py)_2_MgCl] and 3-K(dibenzo-18-c-6).**


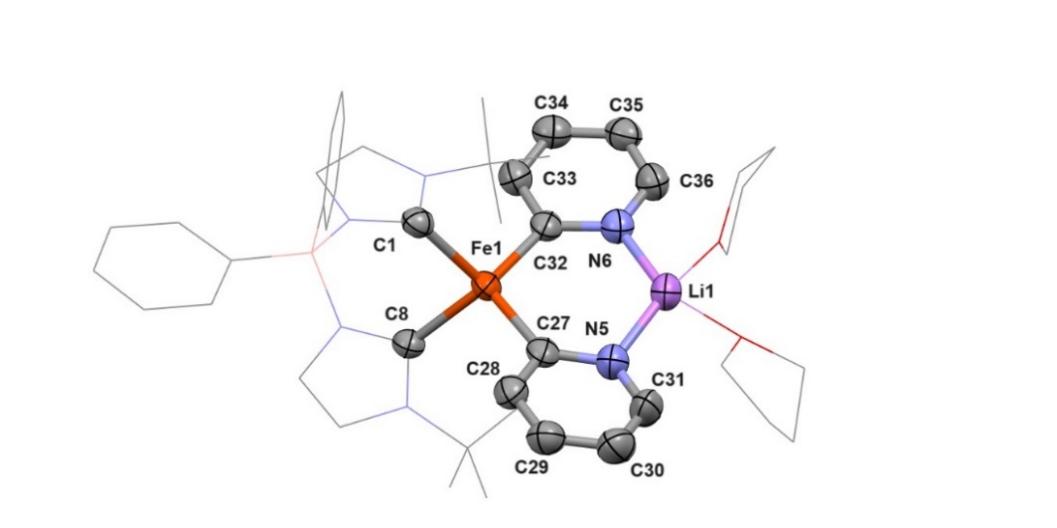


**Figure S1.** Molecular structure of [Ph_2_B(*^t^*BuIm)_2_Fe(Py)_2_Li(THF)_2_] (**2**), as determined by single crystal X-ray diffraction. Ellipsoids are shown at 50% probability level, THF and bis(carbene)borate ligand (except for carbene carbon atoms) represented as wireframe. Hydrogen atoms are omitted for clarity. Color scheme: C, black; N, blue; B, pink; Fe, orange, Li, purple; O, red. Selected bond distances (Å) and angles (deg): Fe1-C1 2.115(3), Fe1-C8 2.108(3), Fe1-C27 2.077(3), Fe1-C32 2.066(3), Li1-N5 2.000(7), Li1-N6 2.008(7), N5-C27 1.370(4), N5-C31 1.348(5), C27-C28 1.402(5), C28-C29 1.370(5), C29-C30 1.384(6), C30-C31 1.353(6), N6-C32 1.374(4), N6-C36 1.345(4), C32-C33 1.404(5), C33-C34 1.373(5), C34-C35 1.376(5), C35-C36 1.357(5); C1-Fe1-C8 95.53(12), C1-Fe1-C32 109.93(12), C8-Fe1-C27 108.09(12), C27-Fe1-C32 113.43(13), C32-N6-Li1 119.0(3), C27-N5-Li1 118.8(3), N5-C27-Fe1 122.5(3), N6-C32-Fe1 123.6(2).


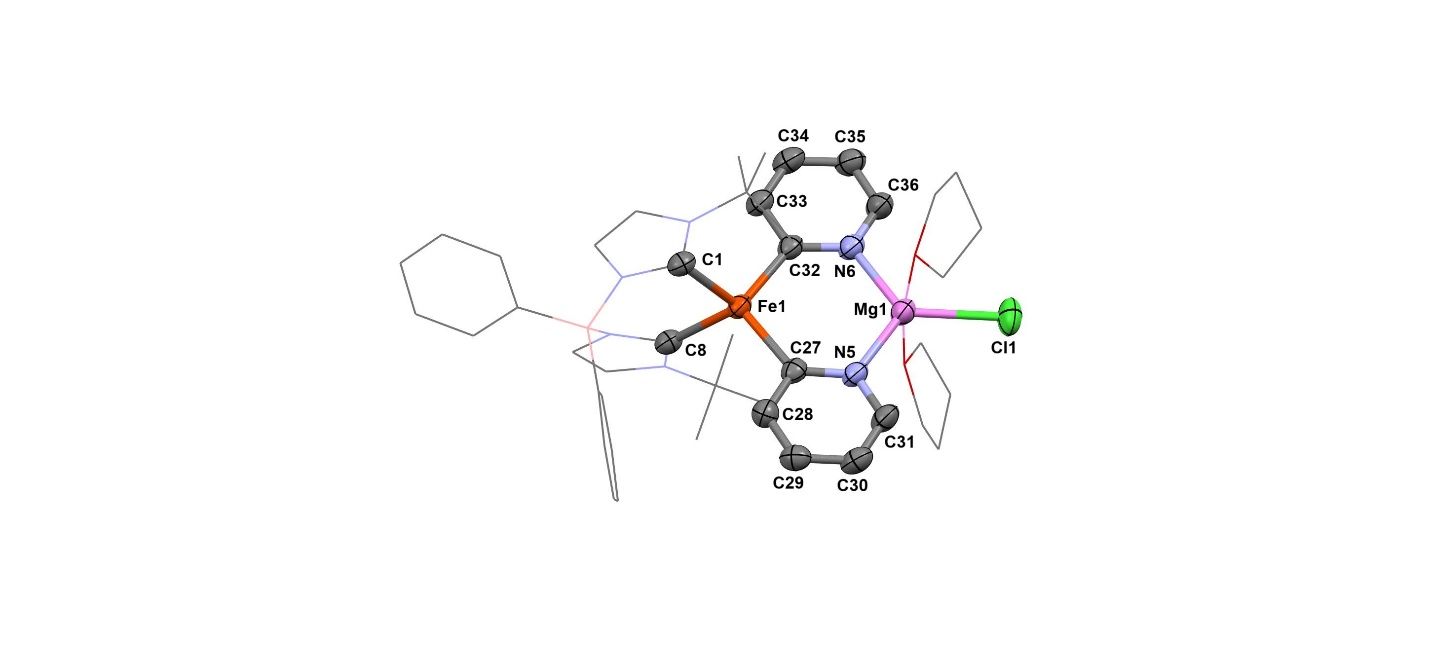


**Figure S2.** Molecular structure of [Ph_2_B(*^t^*BuIm)_2_Fe(2-Py)_2_Mg(THF)_2_], as determined by single crystal X-ray diffraction. Ellipsoids are shown at 50% probability level, THF and bis(carbene)borate ligand (except for carbene carbon atoms) represented as wireframe. Hydrogen atoms are omitted for clarity. Color scheme: C, black; N, blue; B, pink; Fe, orange, Mg, violet; O, red, Cl, green. Selected bond distances (Å) and angles (deg): Fe1-C1 2.137(3), Fe1-C8 2.129(3), Fe1-C27 2.097(3), Fe1-C32 2.100(4), Mg-N5 2.086(3), Mg-N6 2.097(3), Mg-Cl 2.389(1), N5-C27 1.373(4), N5-C31 1.368(4), C27-C28 1.401(5), C28-C29 1.374(5), C29-C30 1.376(5), C30-C31 1.374(5), N6-C32 1.370(4), N6-C36 1.360(5), C32-C33 1.414(5), C33-C34 1.376(5), C34-C35 1.385(5), C35-C36 1.370(5); C1-Fe1-C8 94.23(12), C1-Fe1-C32 113.88(12), C8-Fe1-C27 115.00(12), C27-Fe1-C32 111.02(13), C32-N6-Mg1 121.0(2), C27-N5-Mg1 120.6(2), N5-C27-Fe1 126.3(2), N6-C32-Fe1 125.7(2).


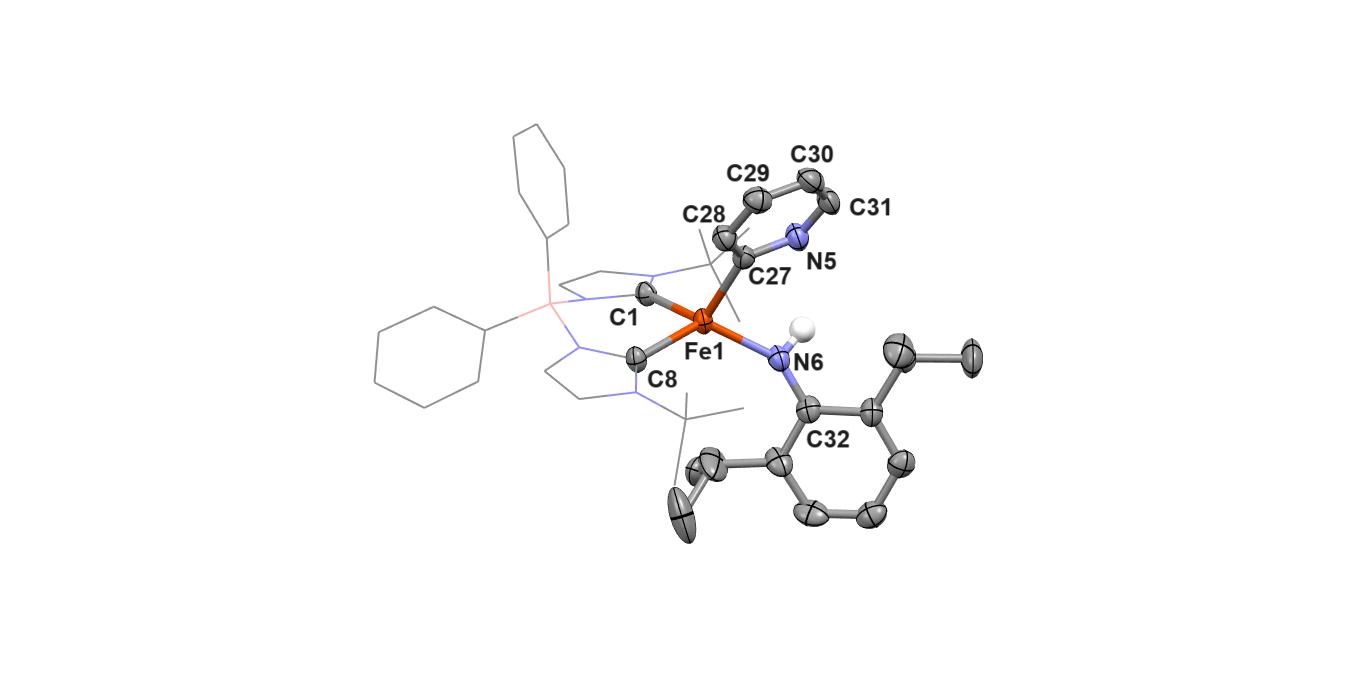


**Figure S3.** Molecular structure of anionic part of [Ph_2_B(*^t^*BuIm)_2_Fe(2-Py)NHDipp][K(dibenzo-18-c-6)(THF)_2_] (**3**-K(dibenzo-18-c-6)), as determined by single crystal X-ray diffraction. Ellipsoids are shown at 50% probability level. Bis(carbene)borate ligand (except for carbene carbon atoms) represented as wireframe. Most hydrogen atoms, counterions and solvent molecules are omitted for clarity. Color scheme: C, black; N, blue; B, pink; Fe, orange; O, red. Selected bond distances (Å) and angles (deg): Fe1-C1 2.126(5), Fe1-C8 2.133(5), Fe1-N6 2.018(4), Fe1-C27 2.088(5), N5-C27 1.368(7), N5-C31 1.327(7), C27-C28 1.398(8), C28-C29 1.384(8), C29-C30 1.372(8), C30-C31 1.385(9), N6-C32 1.362(6); C1-Fe1-C8 95.98(17), C1-Fe1-C27 118.81(18), N6-Fe1-C8 127.91(18), N6-Fe1-C27 95.06(18), C32-N6-Fe1 146.4(3).

**2. NMR spectra for all compounds**


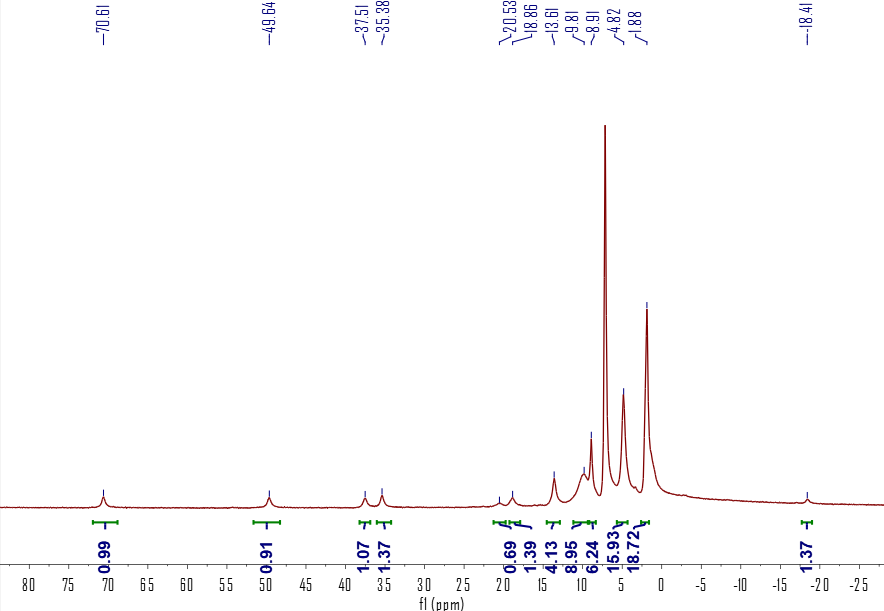


^

*

**Figure S4.** ^1^H NMR spectrum (400 MHz, 25 °C, C_6_D_6_ (*)) of [Ph_2_B(*^t^*BuIm)_2_Fe(2-Py)_2_MgCl(THF)_2_]. Small quantities of an impurity are observed (^).


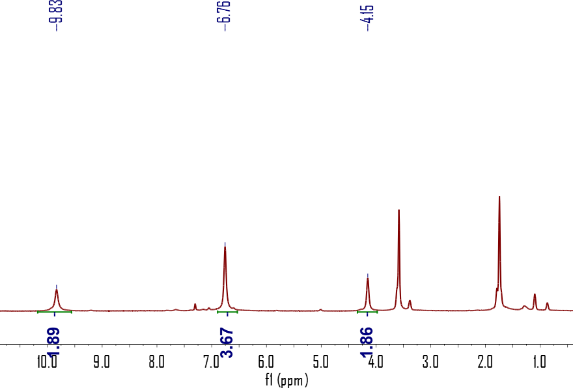

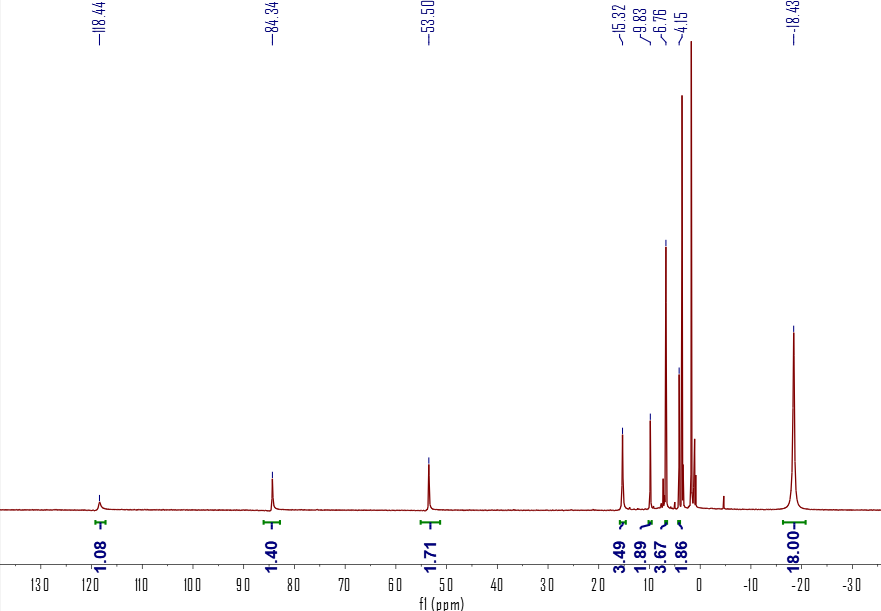


^

^

^

*

*

**Figure S5.** ^1^H NMR spectrum (400 MHz, 25 °C, THF-*d*_8_ (*)) of [Ph_2_B(*^t^*BuIm)_2_Fe(2-Py)_2_MgCl(THF)_2_]. Small quantities of an impurity are observed (^).


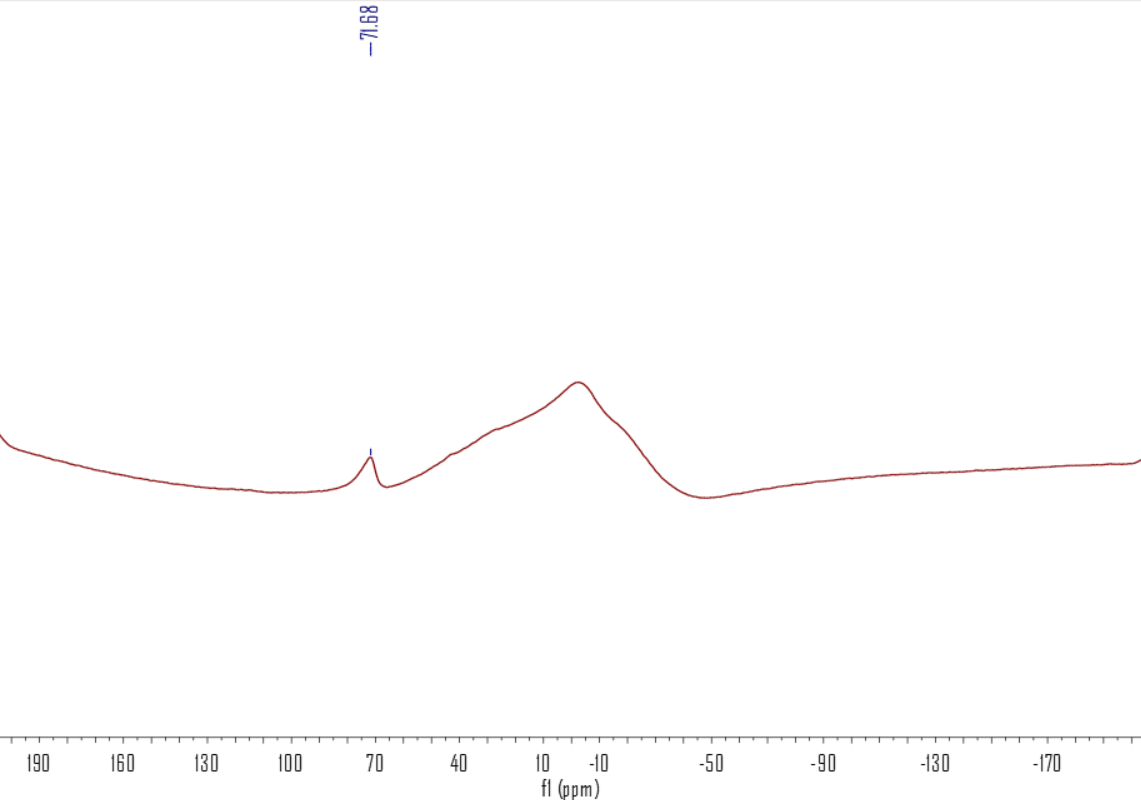


**Figure S6.** ^11^B{^1^H} NMR spectrum (128 MHz, 25 °C, C_6_D_6_) of [Ph_2_B(*^t^*BuIm)_2_Fe(2-Py)_2_MgCl(THF)_2_]. The broad peak in the middle of the spectrum is the background signal of the borosilicate glass NMR tube.


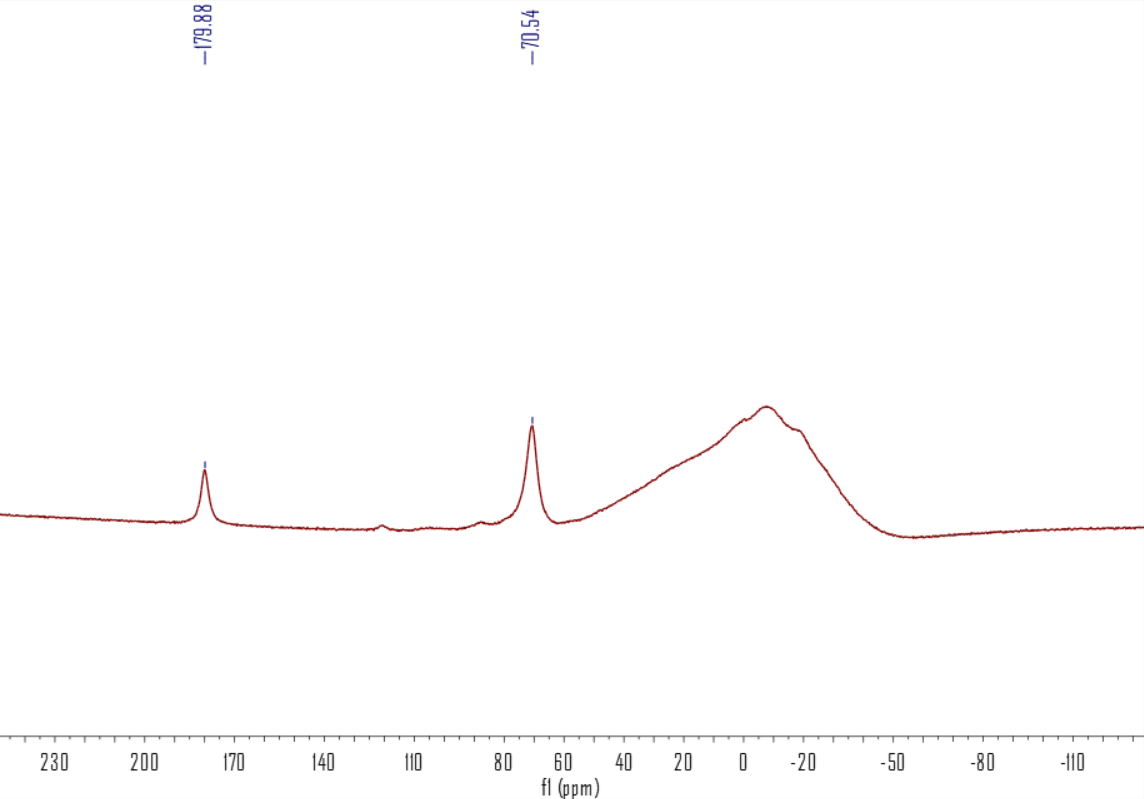


**^**

**Figure S7.** ^11^B{^1^H} NMR spectrum (128 MHz, 25 °C, C_6_D_6_ mixed with drops of THF-*d*_8_) of [Ph_2_B(*^t^*BuIm)_2_Fe(2-Py)_2_MgCl(THF)_2_]. Upon comparison, this signal (^) matches that of the complex in C_6_D_6_. The very broad peak in the right of the spectrum is the background signal of the borosilicate glass NMR tube.


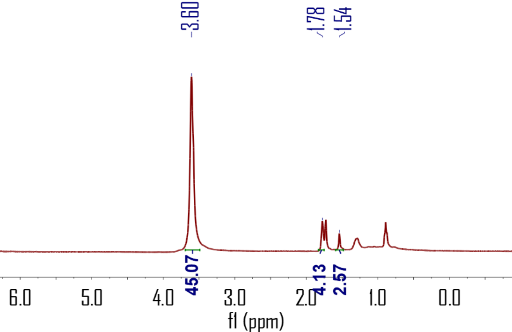
**
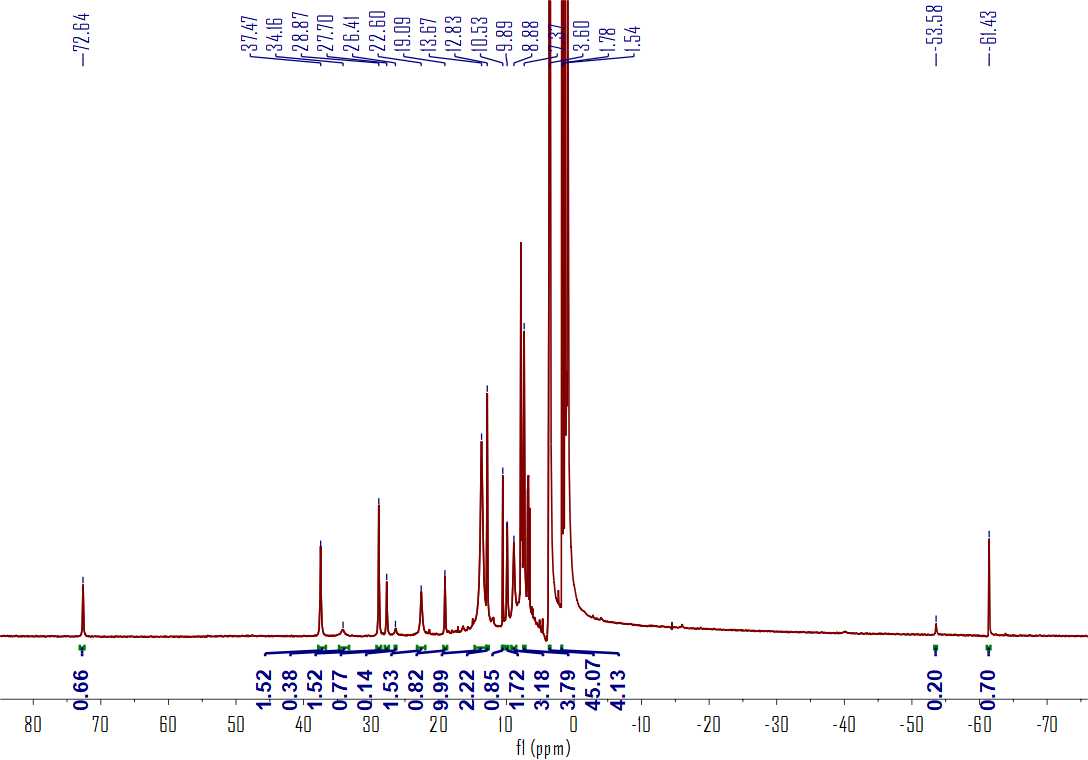
**

*

*

**Pentane**

**Figure S8.** ^1^H NMR spectrum (400 MHz, 25 °C, THF-*d*_8_ (*)) of [Ph_2_B(*^t^*BuIm)_2_Fe(2-Py)NHDipp][K(18-c-6)(THF)_2_].


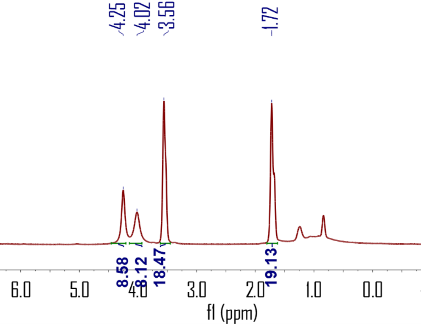
**
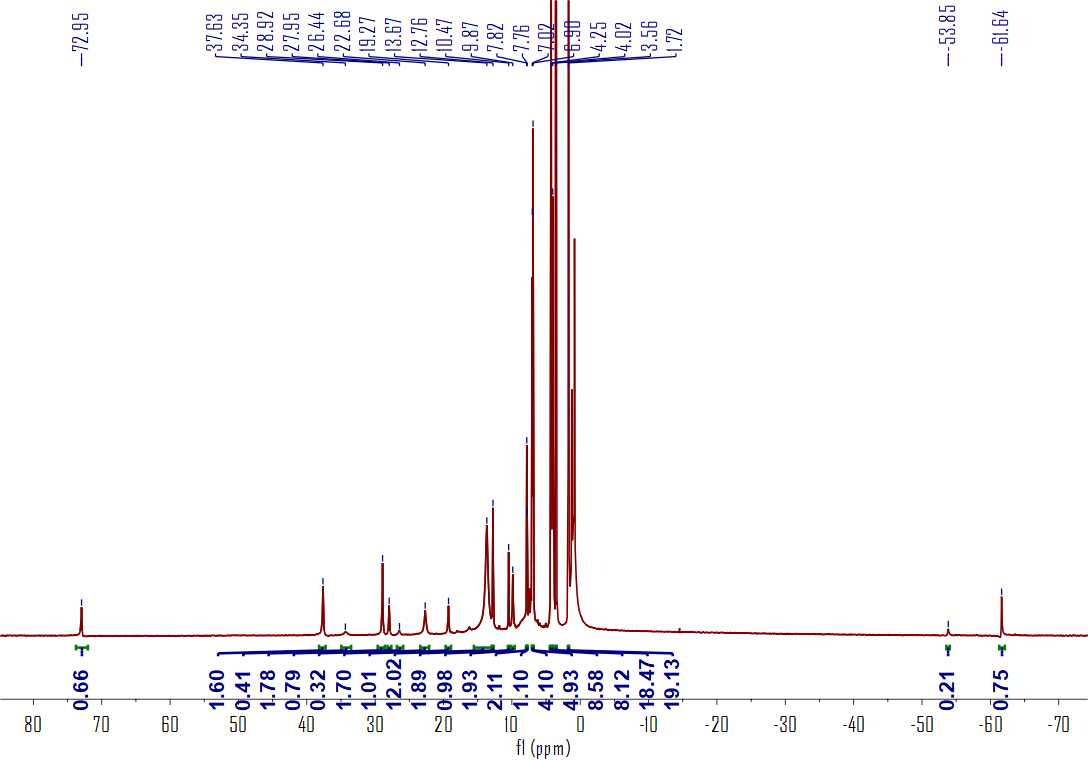
**

*

**Pentane**

*

**Figure S9.** ^1^H NMR spectrum (400 MHz, 25 °C, THF-*d*_8_ (*)) of [Ph_2_B(*^t^*BuIm)_2_Fe(2-Py)NHDipp][K(dibenzo-18-c-6)(THF)_2_].

**
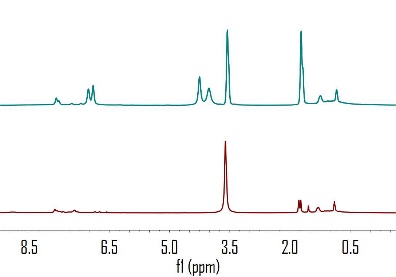

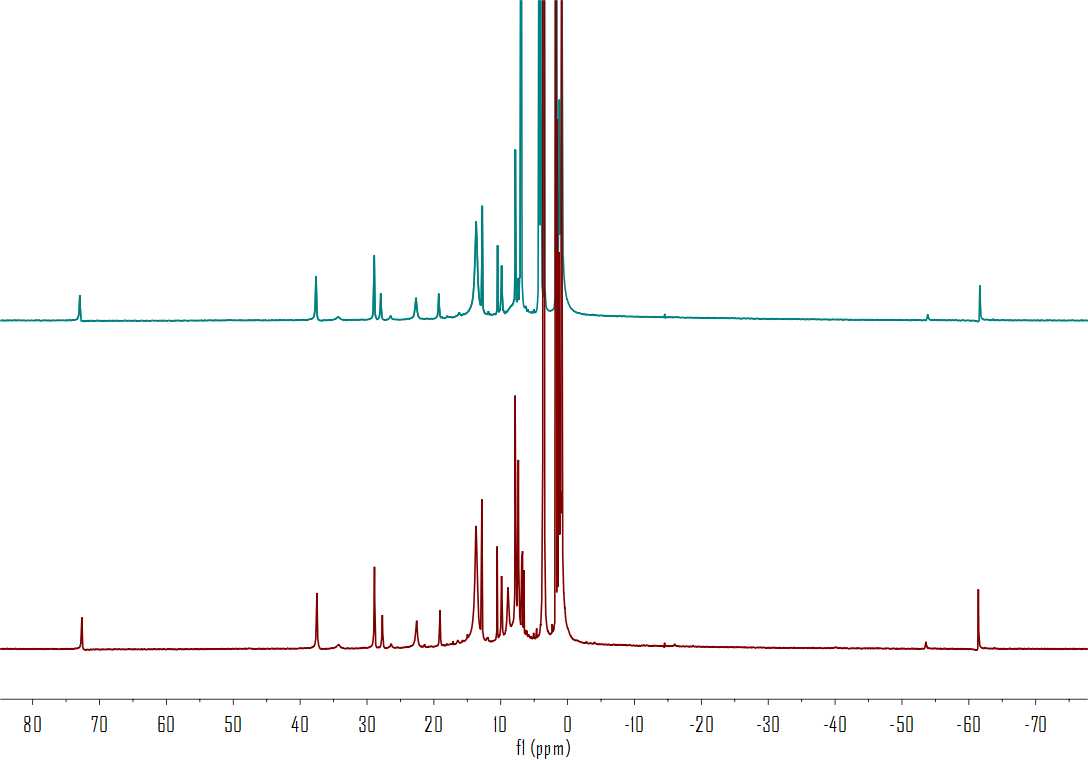
**

**[Ph_2_B(*^t^*BuIm)_2_Fe(2-Py)NHDipp][K(dibenzo-18-c-6)(THF)_2_]**

**[Ph_2_B(*^t^*BuIm)_2_Fe(2-Py)NHDipp][K(18-c-6)(THF)_2_]**

K(dibenzo-18-c-6)

K(18-c-6)

*

*

*

*

**Figure S10.** The comparision of ^1^H NMR spectrum of [Ph_2_B(*^t^*BuIm)_2_Fe(2-Py)NHDipp][K(18-c-6)(THF)_2_] and [Ph_2_B(*^t^*BuIm)_2_Fe(2-Py)NHDipp][K(dibenzo-18-c-6)(THF)_2_] in THF-*d*_8_ (*).

Pyridine equiv.

**
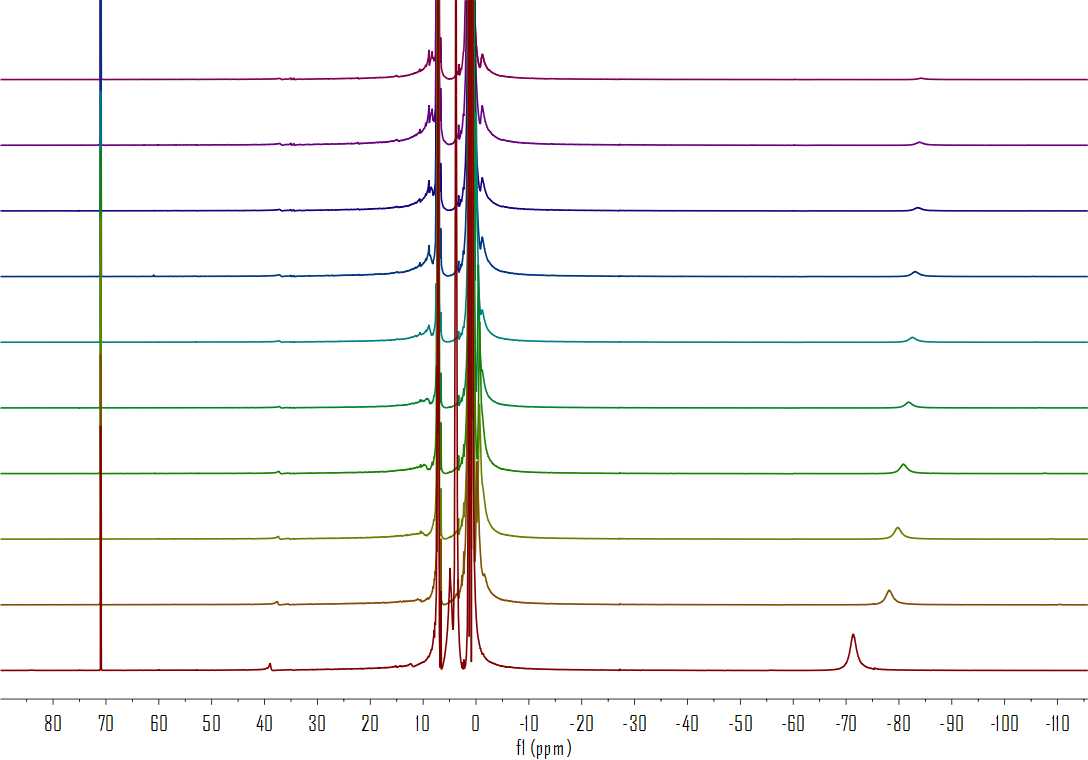
**

2.25

2.00

1.75

1.50

1.25

1.00

0.50

0.75

0.25

**Figure S11.** ^1^H NMR titration (400 MHz, 25 °C, C_6_D_6_): Titration of pyridine into [Ph_2_B(*^t^*BuIm)_2_Fe=NDippLi(THF)_2_NHDipp] leads to the formation of [Ph_2_B(*^t^*BuIm)_2_Fe(Py)_2_Li(THF)_2_] in the range of -120 to 90 ppm.

**
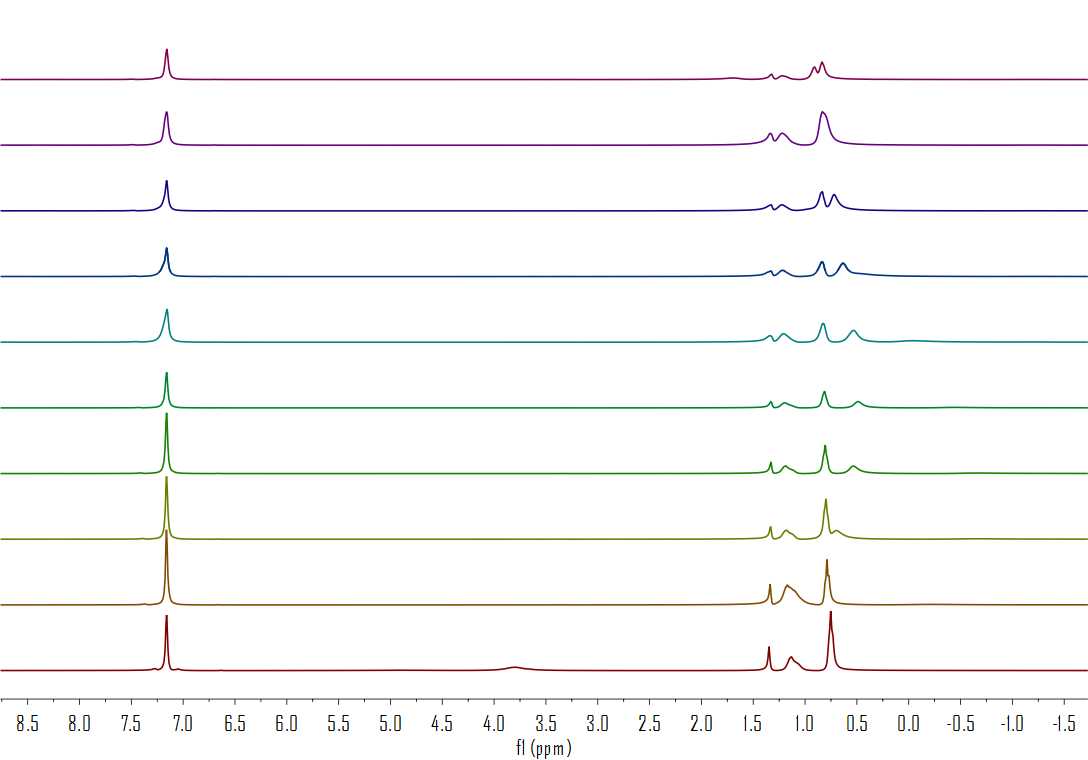
**

Pyridine equiv.

^

^

^

^

^

^

^

^

^

^

0.50

0.25

0.75

1.00

1.25

1.50

1.75

2.00

2.25

**Figure S12.** ^1^H NMR titration (400 MHz, 25 °C, C_6_D_6_ (^)): Titration of pyridine into [Ph_2_B(*^t^*BuIm)_2_Fe=NDippLi(THF)_2_NHDipp] leads to the formation of [Ph_2_B(*^t^*BuIm)_2_Fe(Py)_2_Li(THF)_2_] in the range of -1.5 to 8.5 ppm.

**
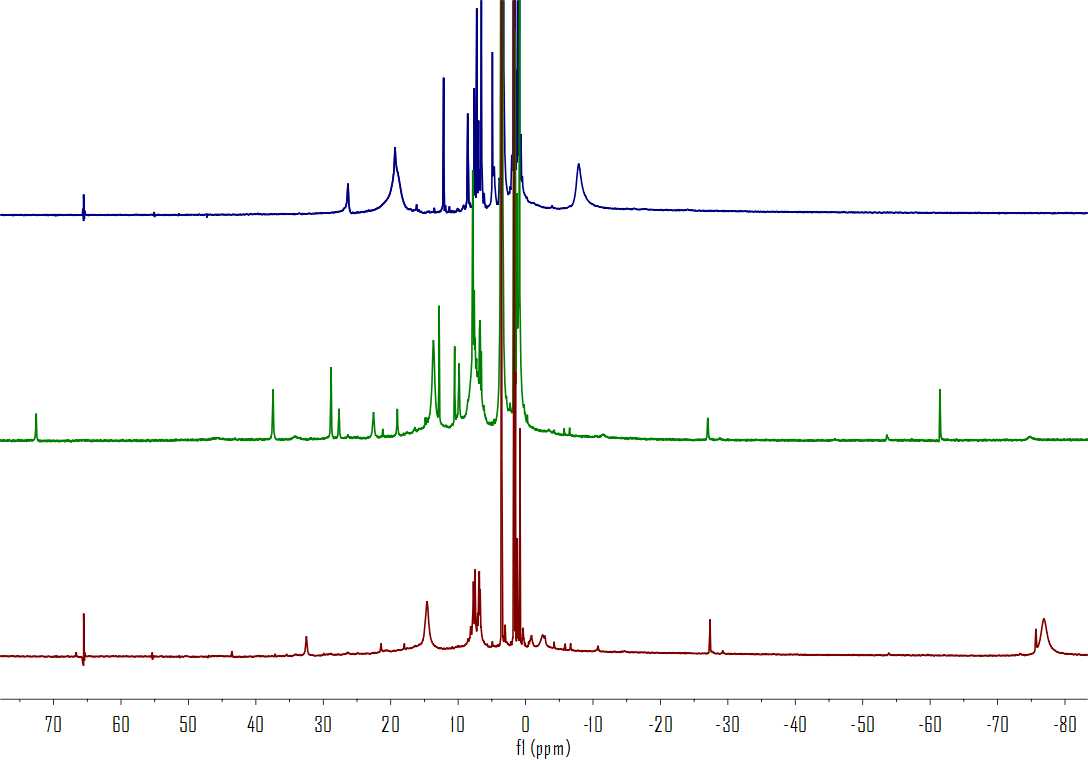
**

**[Ph_2_B(*^t^*BuIm)_2_Fe(2-Py)NHDipp][K(dibenzo-18-c-6)(THF)_2_]**

**[Ph_2_B(*^t^*BuIm)_2_Fe=NDipp][K(18-c-6)(THF)_2_]**

**[Ph_2_B(*^t^*BuIm)_2_Fe(*^i^*PrN)_2_CNDipp][K(18-c-6)(THF)_2_]**

**+ 1eq. *^i^*PrNCN*^i^*Pr, 75 ^o^C., 2h**

**+ 1eq. pyridine, r.t., 2h**

**Figure S13.** ^1^H NMR (400 MHz, 25 °C, THF-*d*_8_) of the reaction of [Ph_2_B(*^t^*BuIm)_2_Fe=NDipp][K(18-c-6)(THF)_2_] with pyridine and *N,N'*-diisopropylcarbodiimide (*^i^*PrNCN*^i^*Pr).


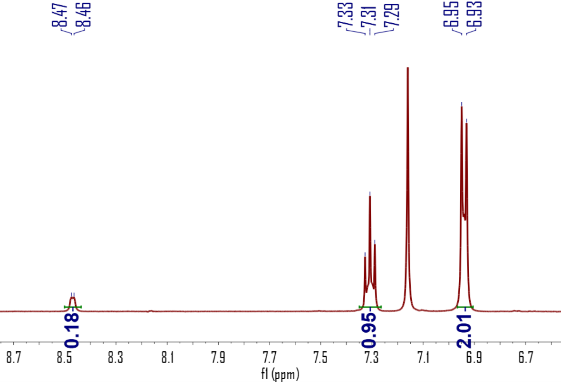

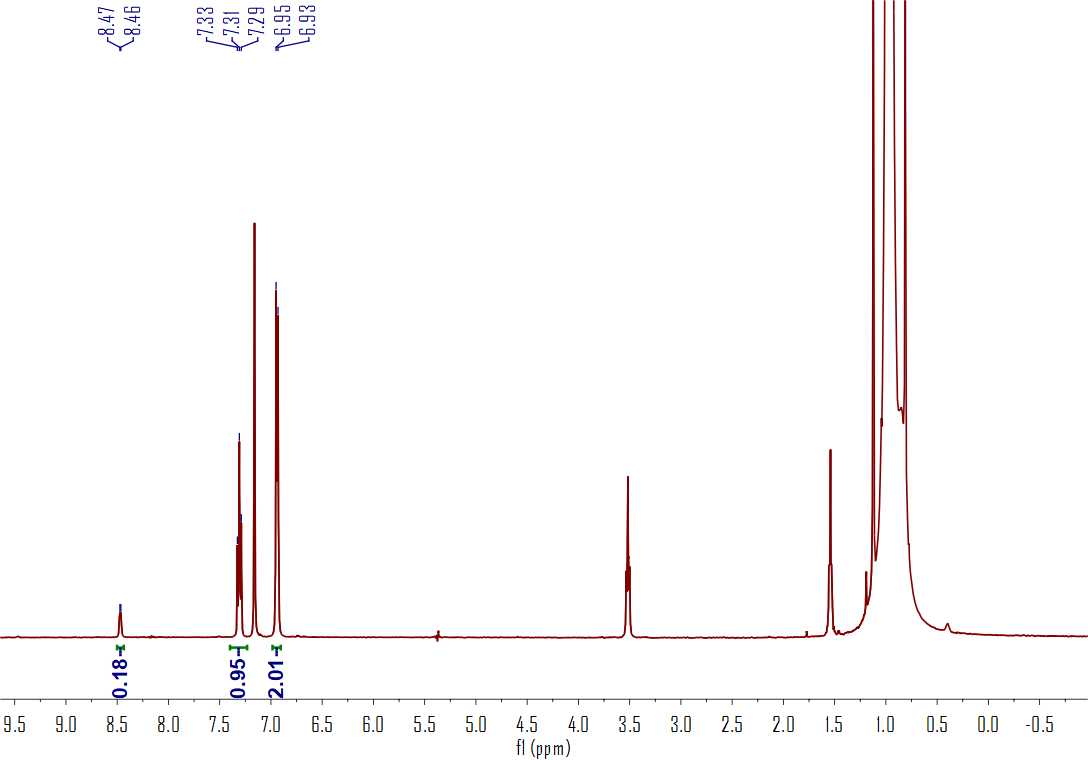


*

*

**Figure S14.** ^1^H NMR spectrum (500 MHz, 25 °C, C_6_D_6_) of *α*-*d*_2_ pyridine. Residual THF is noted as *.


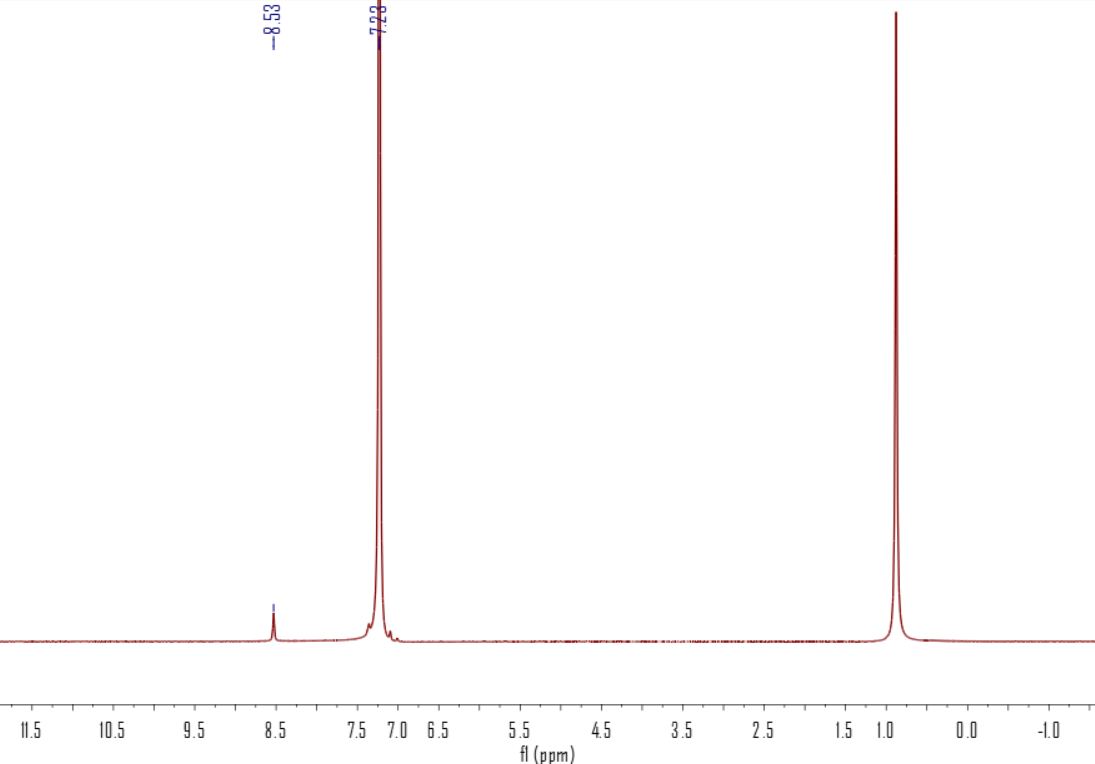


**Figure S15.** ^2^H NMR spectrum (92 MHz, 25 °C, C_6_D_6_) of *α*-*d*_2_ pyridine.


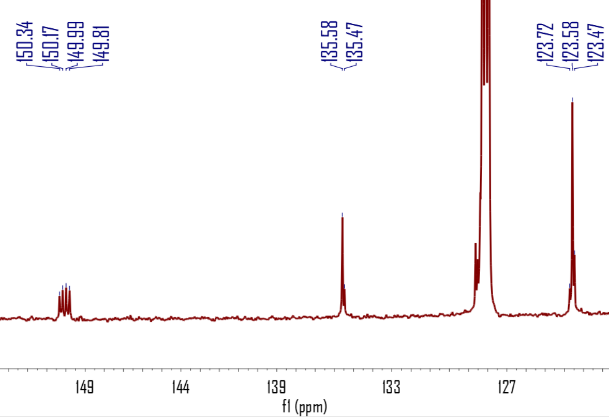

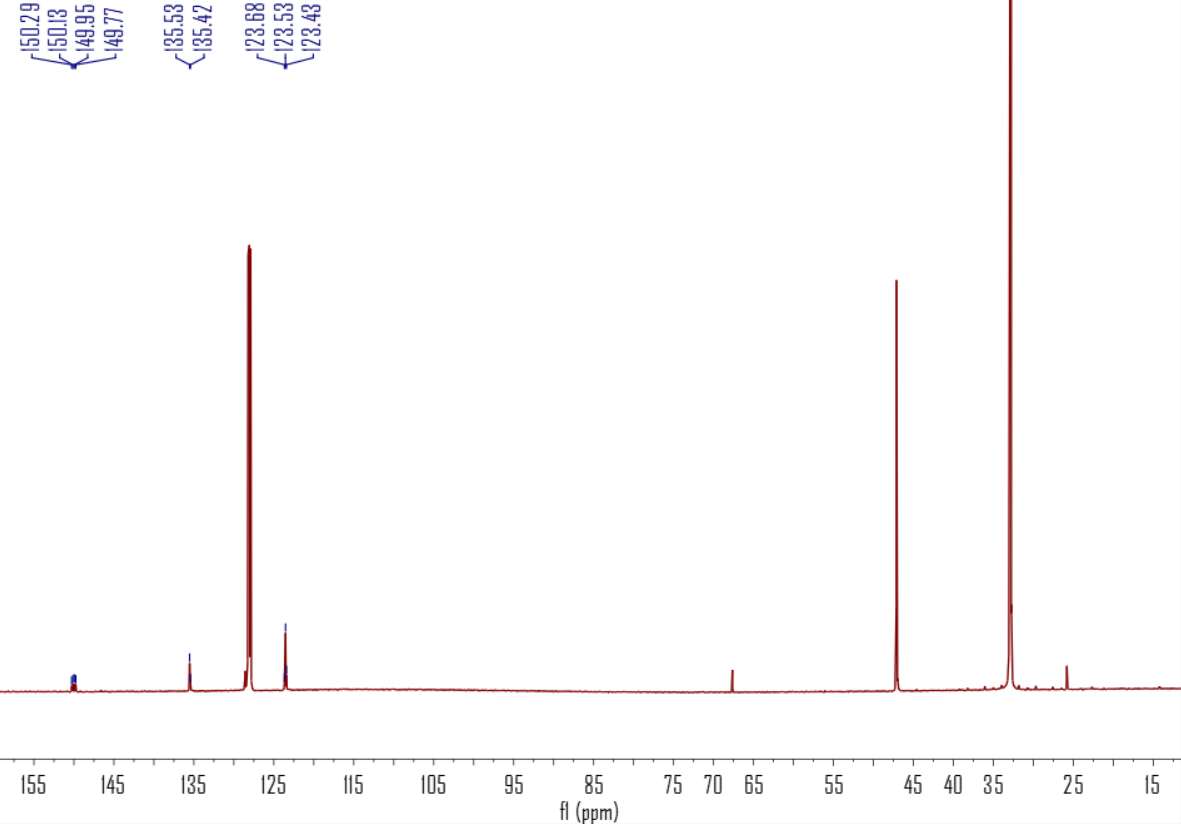


*

*

**Figure S16.** ^13^C{^1^H} NMR spectrum (151 MHz, 25 °C, C_6_D_6_) of *α*-*d*_2_ pyridine. Residual THF is noted as *.


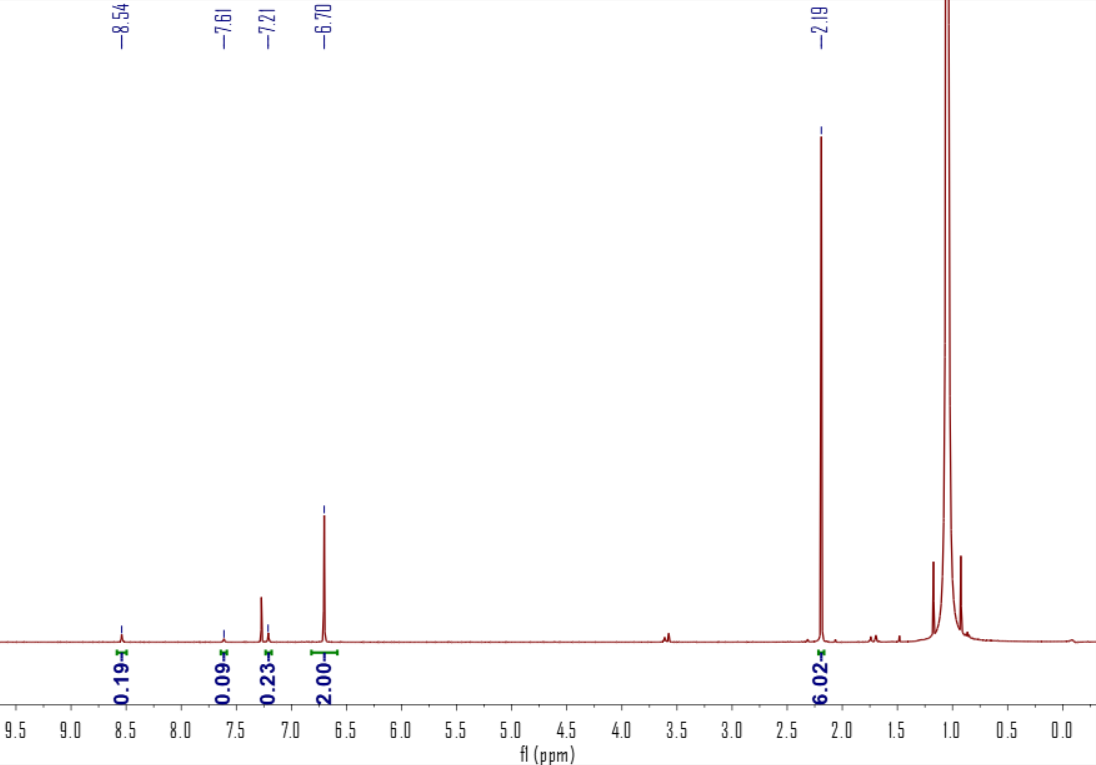


**Figure S17.** ^1^H NMR spectrum (500 MHz, 25 °C, THF-*d*_8_) of *d*_5_-deuterated pyridine.


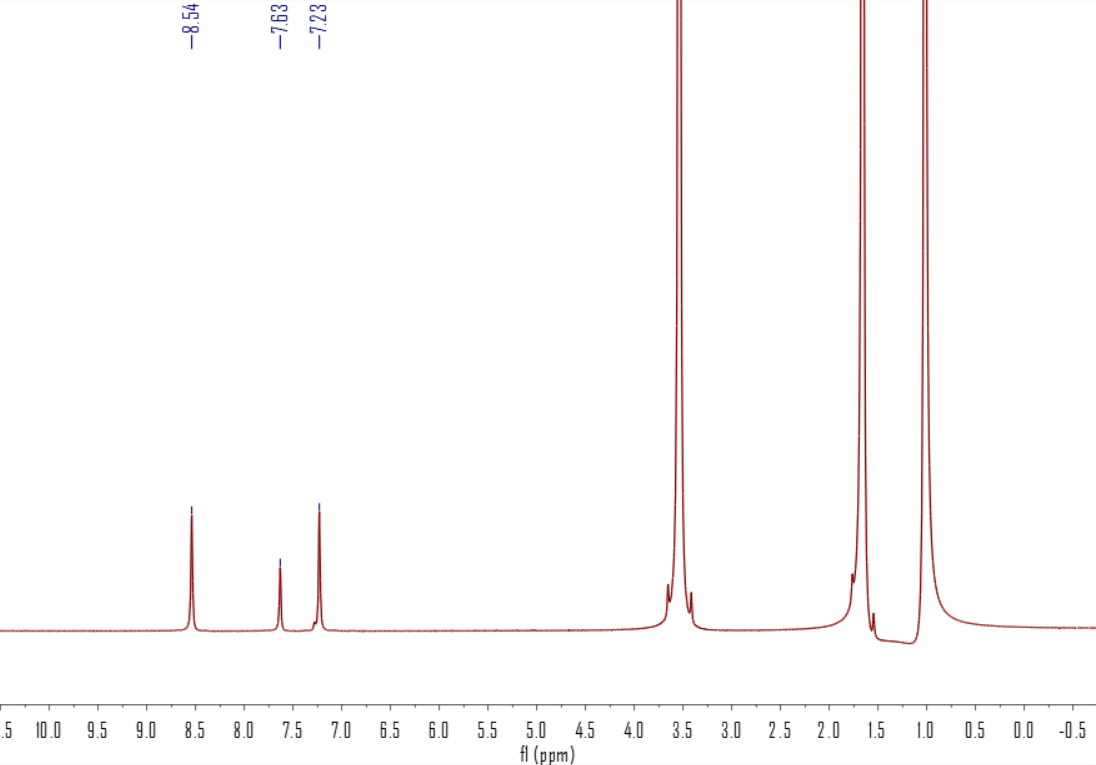


*

*

**Figure S18.** ^2^H NMR spectrum (92 MHz, 25 °C, THF-*d*_8_ (*)) of *d*_5_-deuterated pyridine.


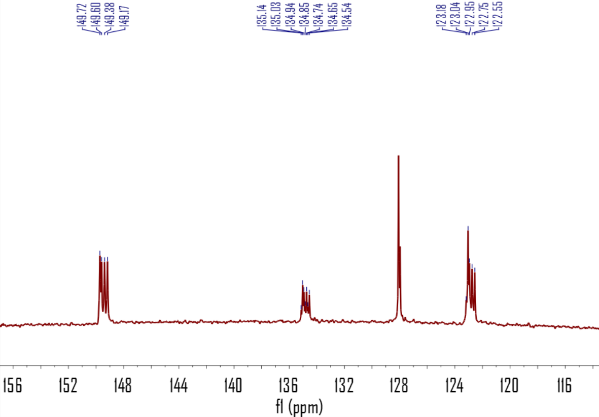

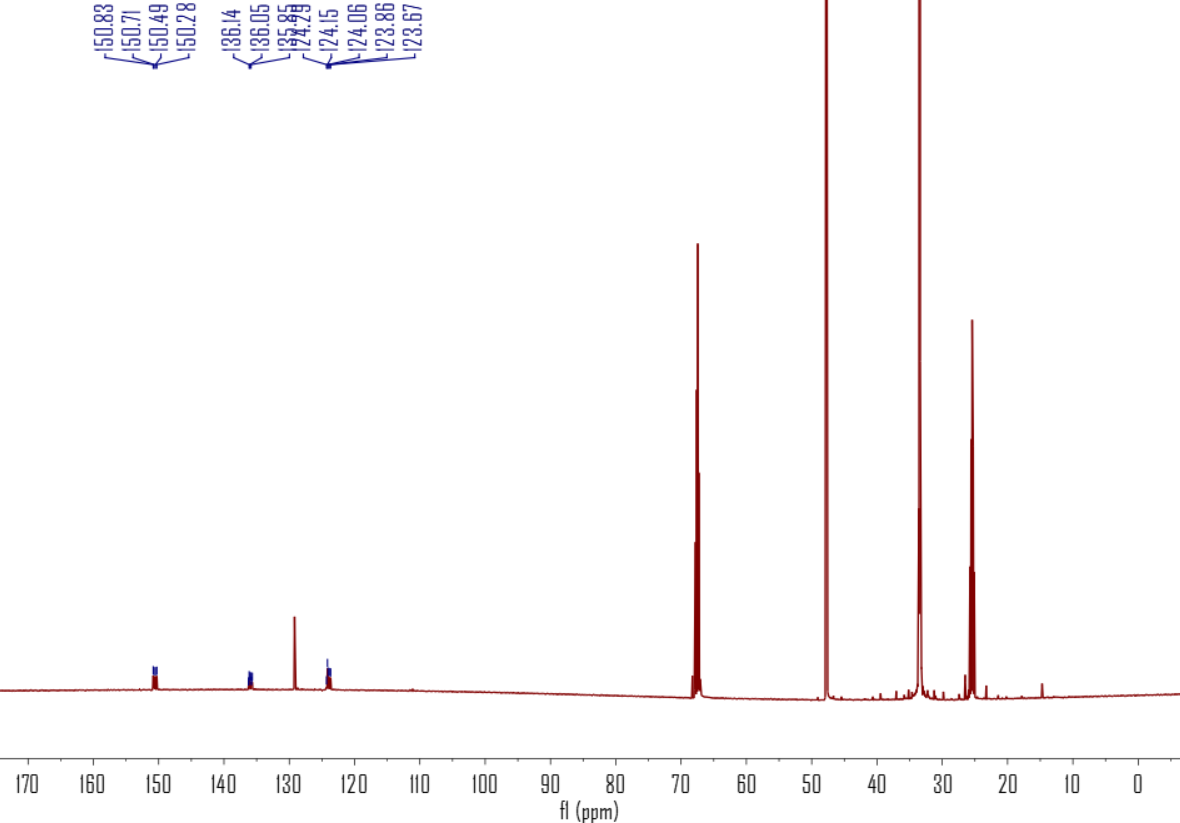


*

*

**Figure S19.** ^13^C{^1^H} NMR spectrum (126 MHz, 25 °C, *d*_8_-THF(*)) of *d*_5_-deuterated pyridine.


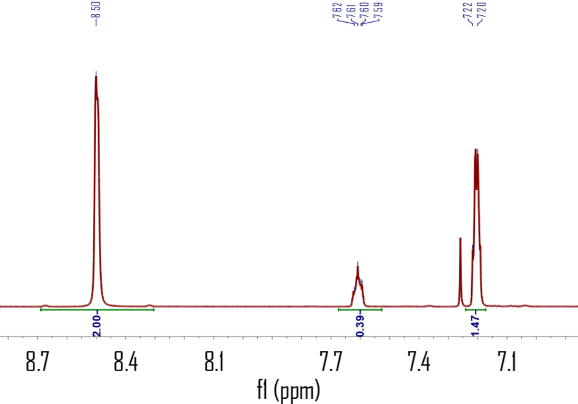

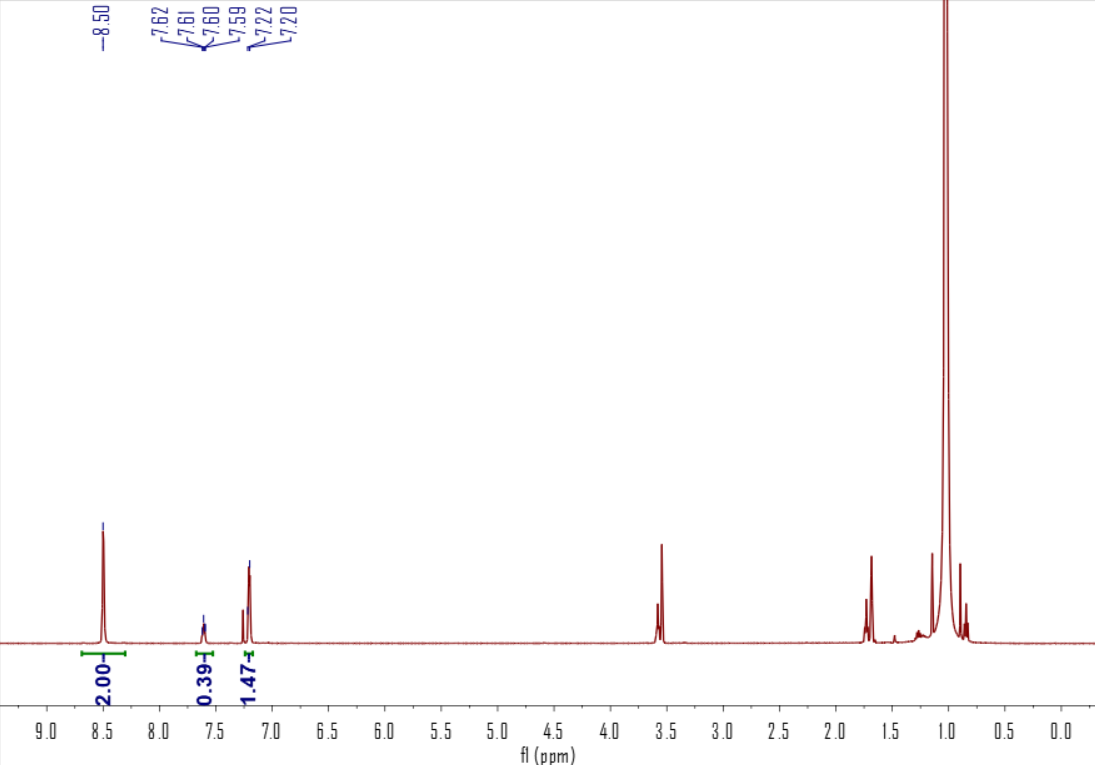


*

*

*

*

**Figure S20.** ^1^H NMR spectrum (500 MHz, 25 °C, *d*_8_-THF(*)) of *β,γ*-deuterated pyridine. Residual THF is noted as *.


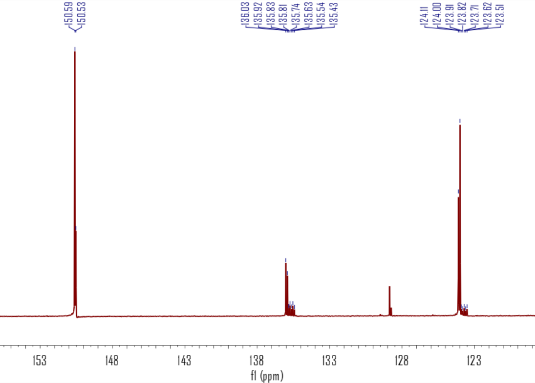

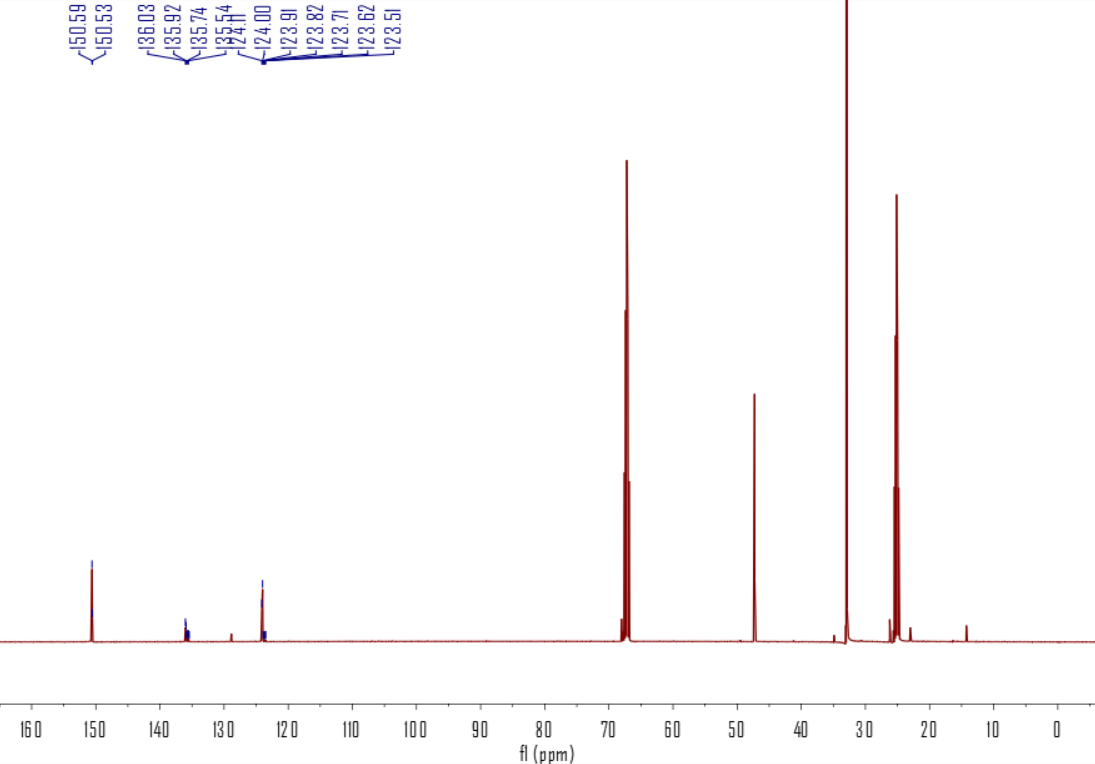


*

*

**Figure S21.** ^13^C{^1^H} NMR spectrum (126 MHz, 25 °C, *d*_8_-THF(*)) of *β,γ*-deuterated pyridine.

**3. HRMS samples of hydrogen isotope exchange between *d*_0_- and d_5_-pyridines.**


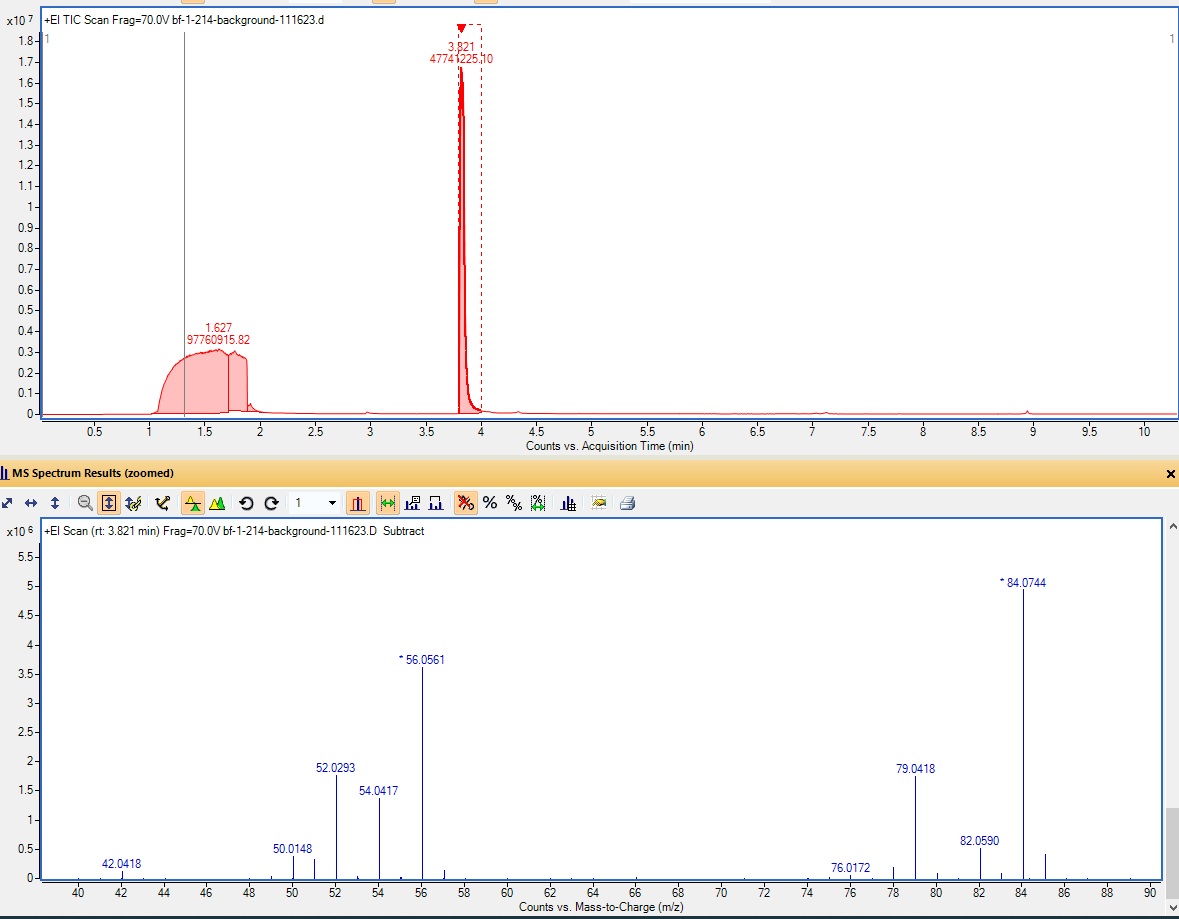


**(a)**


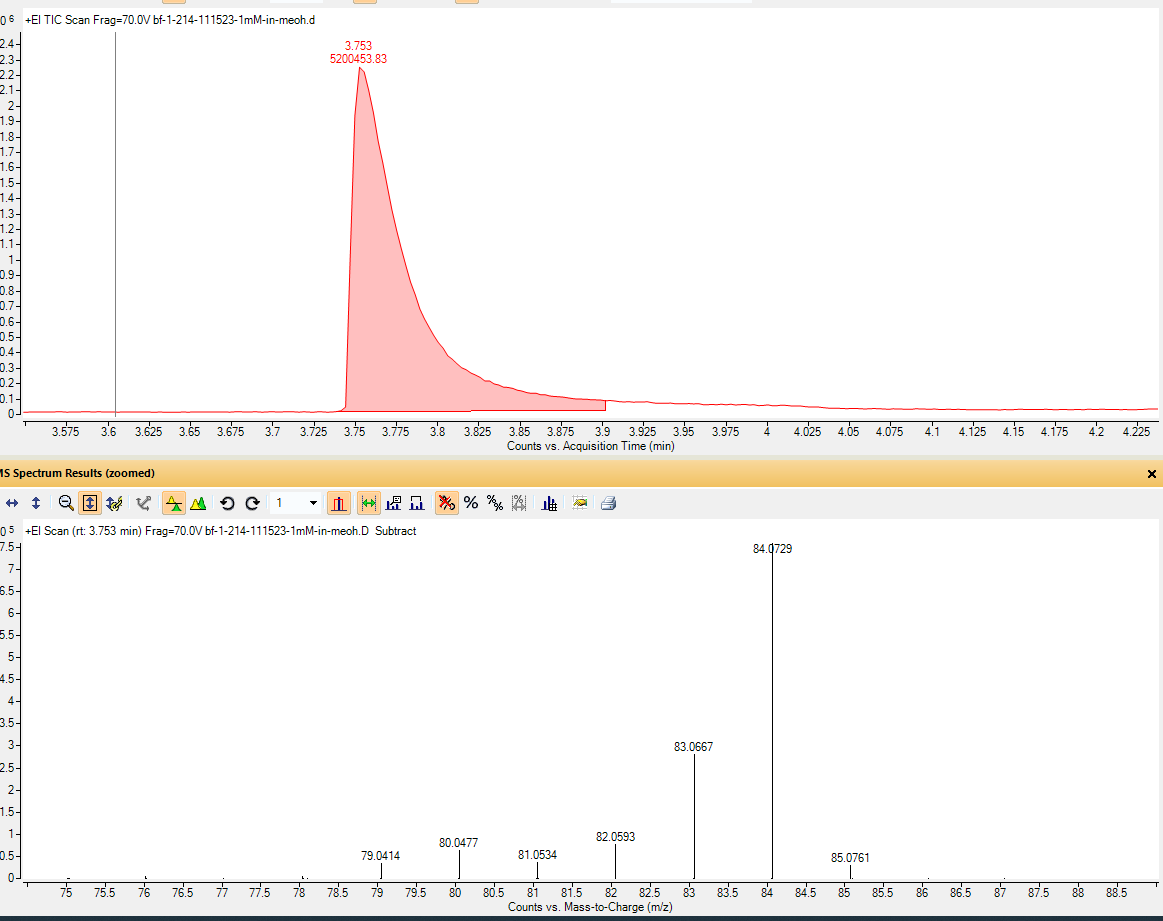


**(b)**

**Figure S22.** HRMS of (a) solution of *d*_0_ and *d*_5_ pyridines as background. (b) solution of containing H/D mixed (*d*_1-4_-pyridines) after the HIE reaction.

**4. UV-vis spectra**


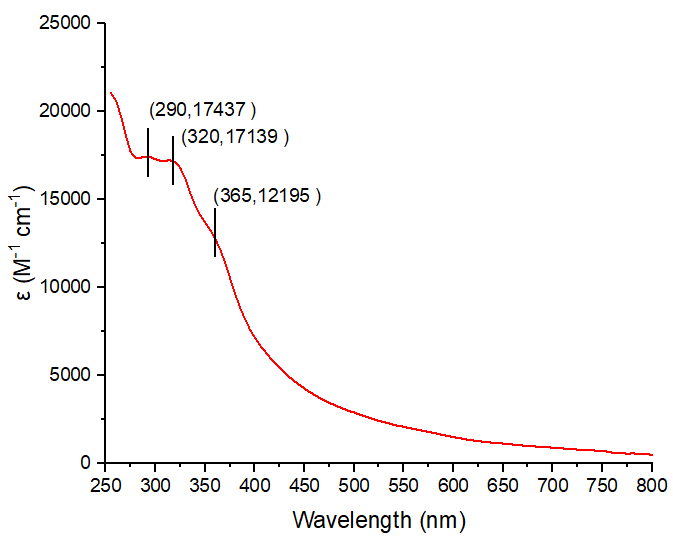


**Figure S23.** UV-vis spectrum of [Ph_2_B(*^t^*BuIm)_2_Fe(Py)_2_Li(THF)_2_] measured at 25 °C in THF.


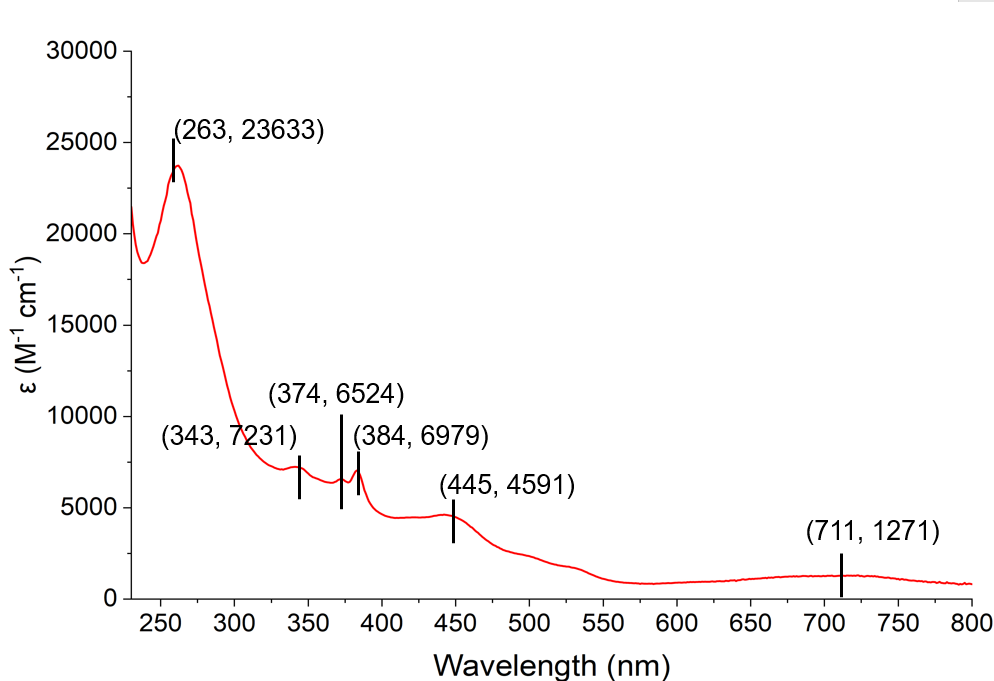


**Figure S24.** UV-vis spectrum of [Ph_2_B(*^t^*BuIm)_2_Fe(Py)_2_MgCl(THF)_2_] measured at 25 °C in THF.


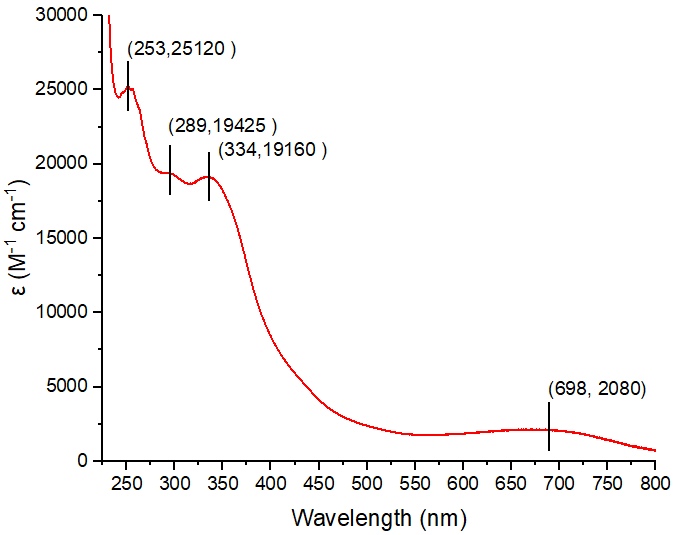


**Figure S25.** UV-vis spectrum of [Ph_2_B(*^t^*BuIm)_2_Fe(2-Py)NHDipp][K(18-c-6)] measured at 25 °C in THF.


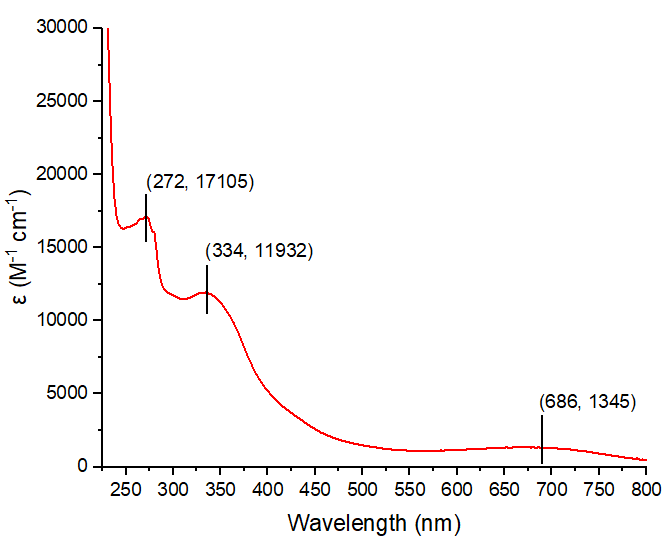


**Figure S26.** UV-vis spectrum of [Ph_2_B(*^t^*BuIm)_2_Fe(2-Py)NHDipp][K(dibenzo-18-c-6)(THF)_2_] measured at 25 °C in THF.

**Crystallographic Data Collection**

[Ph_2_B(*^t^*BuIm)_2_Fe(2-Py)_2_Li] (**2**)

**Data collection**

The data collection was carried out using Mo Kα radiation (graphite monochromator) with a frame time of 1, 5, and 80 seconds and a detector distance of 5.0 cm. A collection strategy was calculated and complete data to a resolution of 0.84 Å with a redundancy of 13.53 were collected. Twelve major sections of frames were collected with 1.00º ϕ and ω scans. A total of 2178 frames were collected. The total exposure time was 17.34 hours. The frames were integrated with the Bruker SAINT software package^7^ using a narrow-frame algorithm. The integration of the data using a monoclinic unit cell yielded a total of 105443 reflections to a maximum θ angle of 25.09° (0.84 Å resolution), of which 7653 were independent (average redundancy 13.778, completeness = 99.6%, R_int_ = 9.77%, R_sig_ = 4.07%) and 5433 (70.99%) were greater than 2σ(F^2^). The final cell constants of a = 13.8061(8) Å, b = 18.1602(10) Å, c = 17.8179(10) Å, β = 104.9822(18)°, volume = 4315.5(4) Å^3^, are based upon the refinement of the XYZ-centroids of 9977 reflections above 20 σ(*I*) with 4.485° < 2θ < 49.44°. Data were corrected for absorption effects using the Multi-Scan method (SADABS).^8^ The ratio of minimum to maximum apparent transmission was 0.727. The calculated minimum and maximum transmission coefficients (based on crystal size) are 0.8620 and 0.9870.

**Structure solution and refinement**

The space group *P*2_1_/*c* was determined based on intensity statistics and systematic absences. The structure was solved with intrinsic-methods and refined with full-matrix-least squares using the SHELX suite of programs in the Olex2. All non-hydrogen atoms were refined with anisotropic displacement parameters. The hydrogen atoms were placed in ideal positions and refined as riding atoms with relative isotropic displacement parameters. Disorder was found on tetrahydrofuran moieties. Site occupancies and constraints/restraints were applied. The final anisotropic full-matrix least-squares refinement on F^2^ with 534 variables converged at R1 = 5.65%, for the observed data and wR2 = 17.16% for all data. The goodness-of-fit was 1.098. The largest peak in the final difference electron density synthesis was 0.314 e^-^/Å^3^ and the largest hole was -0.521 e^-^/Å^3^ with an RMS deviation of 0.061 e^-^/Å^3^. On the basis of the final model, the calculated density was 1.192 g/cm^3^ and F(000), 1648 e^-^.

Table S1. Crystal data and structure refinement for [Ph_2_B(*^t^*BuIm)_2_Fe(2-Py)_2_Li] (**2**)

| Empirical formula | C_44_H_56_BFeLiN_6_O_2_ | |
| --- | --- | --- |
| Formula weight | 774.54 | |
| Crystal color, shape, size | brown plate, 0.392 × 0.144 × 0.034 mm^3^ | |
| Temperature | 173(2) K | |
| Wavelength | 0.71073 Å | |
| Crystal system, space group | Monoclinic, *P*2_1_/*c* | |
| Unit cell dimensions | a = 13.8061(8) Å | α = 90°. |
|  | b = 18.1602(10) Å | β = 104.9822(18)°. |
|  | c = 17.8179(10) Å | γ = 90°. |
| Volume | 4315.5(4) Å^3^ | |
| Z | 4 | |
| Density (calculated) | 1.192 Mg/m^3^ | |
| Absorption coefficient | 0.391 mm^-1^ | |
| F(000) | 1648 | |
| *Data collection* | | |
| Diffractometer | Venture D8, Bruker | |
| Source | Iμ3.0, Incoatec | |
| Detector | Photon III | |
| Theta range for data collection | 2.014 to 25.087°. | |
| Index ranges | -16<=h<=16, -21<=k<=21, -21<=l<=21 | |
| Reflections collected | 105443 | |
| Independent reflections | 7653 [R_int_ = 0.0977] | |
| Observed Reflections | 5433 | |
| Completeness to theta = 25.087° | 99.6 % | |
| Solution and Refinement | | |
| Absorption correction | Multi-Scan | |
| Max. and min. transmission | 0.7452 and 0.5421 | |
| Refinement method | Full-matrix least-squares on F^2^ | |
| Weighting scheme | w = [σ^2^Fo^2^ + AP^2^ + BP]^-1^, with  P = (Fo^2^ + 2 Fc^2^)/3, A = 0.0803, B = 3.2002 | |
| Data / restraints / parameters | 7653 / 633 / 534 | |
| Goodness-of-fit on F^2^ | 1.098 | |
| Final R indices [I>2σ(I)] | R1 = 0.0565, wR2 = 0.1416 | |
| R indices (all data) | R1 = 0.0902, wR2 = 0.1716 | |
| Largest diff. peak and hole | 0.314 and -0.521 e.Å^-3^ | |

[Ph_2_B(*^t^*BuIm)_2_Fe(2-Py)_2_MgCl]

**Data collection**

The data collection was performed using 0.5° ω and φ scans, frame times of 3 and 90 s, and a detector distance of 40 mm. Overall, 3004 frames were collected with a total exposure time of 52.60 hours. The frames were integrated with the SAINT V8.41 package^7^ using a narrow-frame algorithm.⁠ The integration of the data using a monoclinic unit cell yielded 164504 reflections to a maximum θ angle of 25.17° (0.84 Å resolution), of which 9476 were independent (average redundancy 17.36, completeness = 99.3%, R_int_ = 11.20%, R_sig_ = 3.66%) and 7291 (76.9%) were greater than 2σ(F2). The final cell constants of a = 37.463(9) Å, b = 17.580(3) Å, c = 18.155(4) Å, α = 90°, β = 117.353(12)°, γ = 90°, volume = 10620(4) Å^3^, are based upon the refinement of the XYZ-centroids of 9094 reflections above 20 σ(*I*) with 2.24° < 2θ < 25.01°. Data were corrected for absorption effects using the Multi-Scan method in SADABS 2016/2.^8^ The calculated minimum and maximum transmission coefficients (based on crystal size) are 0.929 and 0.985.⁠ Additional crystal and refinement information can be found in the tables.

**Data collection**

The space group *C*2/*c*(15) was determined based on intensity statistics and systematic absences. The structure was solved by SHELXT 2018/2 and refined with full-matrix least squares/difference Fourier cycles using SHELXL-2019/2; Z = 8 for the formula unit C_47_H_62_BClFeMgN_6_O_2.75_.⁠ Non-hydrogen atoms were refined with anisotropic displacement parameters. The hydrogen atoms were placed in ideal positions and refined as riding atoms with relative isotropic displacement parameters. The final anisotropic full-matrix least-squares refinement on *F*^2^ with 630 variables against 9476 data points and 961 restraints converged at *R*_1_ = 6.18%, for the observed data and w*R*_2_ = 20.45% for all data. The goodness-of-fit on *F*^2^ was 1.06. The largest peak in the final difference electron density synthesis was 0.75 e^−^/Å^3^ and the deepest hole was −0.41 e^−^/Å^3^ with an RMS deviation of 0.089 e^−^/Å^3^. On the basis of the final model, the calculated density was 1.10 g/cm^3^ and *F*(000), 3744 e^−^. Disorder was refined for a coordinated THF moiety and a co-crystallized THF solvent molecule using restraints and constraints.

Table S2. Crystal data and structure refinement for [Ph_2_B(*^t^*BuIm)_2_Fe(2-Py)_2_MgCl]

| Empirical formula | C_47_H_62_BClFeMgN_6_O_2.75_ | |
| --- | --- | --- |
| Formula weight | 881.44 | |
| Crystal color, shape, size | yellow plate, 0.195 × 0.137 × 0.039 mm^3^ | |
| Temperature | 153(2) K | |
| Wavelength | 0.71073 Å | |
| Crystal system, space group | Monoclinic, C2/c | |
| Unit cell dimensions | a = 37.463(9) Å | α = 90°. |
|  | b = 17.580(3) Å | β = 117.353(12)°. |
|  | c = 18.155(4) Å | γ = 90°. |
| Volume | 10620(4) Å^3^ | |
| Z | 8 | |
| Density (calculated) | 1.103 Mg/m^3^ | |
| Absorption coefficient | 0.386 mm^-1^ | |
| F(000) | 3744 | |
| *Data collection* | | |
| Diffractometer | Venture D8, Bruker | |
| Source | Incoatec Iµ3.0, Photon III | |
| Theta range for data collection | 2.005 to 25.174° | |
| Index ranges | -44<=h<=44, -20<=k<=18, -21<=l<=21 | |
| Reflections collected | 164504 | |
| Independent reflections | 9476 [R_int_ = 0.1120] | |
| Observed Reflections | 7291 | |
| Completeness to theta = 25.087° | 99.3 % | |
| Solution and Refinement | | |
| Absorption correction | Semi-empirical from equivalents | |
| Max. and min. transmission | 0.7452 and 0.6352 | |
| Solution | Intrinsic methods | |
| Refinement method | Full-matrix least-squares on F^2^ | |
| Weighting scheme | w = [σ^2^Fo^2^ + AP^2^ + BP]^-1^, with  P = (Fo^2^ + 2Fc^2^)/3, A = 0.124900, B = 15.1901 | |
| Data / restraints / parameters | 9476 / 961 / 630 | |
| Goodness-of-fit on F^2^ | 1.065 | |
| Final R indices [I>2σ(I)] | R1 = 0.0618, wR2 = 0.1845 | |
| R indices (all data) | R1 = 0.0821, wR2 = 0.2045 | |
| Largest diff. peak and hole | 0.750 and -0.414 e.Å^-3^ | |

[Ph_2_B(*^t^*BuIm)_2_Fe(2-Py)NHDipp][K(dibenzo-18-c-6)(THF)_2_] **3-K(dibenzo-18-c-6)**.

**Data collection**

The data collection was carried out using Mo Kα radiation (graphite monochromator) with a frame time of 60, 45, 1, and 0.75 seconds and a detector distance of 7.00 cm. A collection strategy was calculated and complete data to a resolution of 0.77 Å (twelve sets of frames) were collected with 0.75º ω and φ scans. A total of 3376 frames were collected. The total exposure time was 23.54 hours. The frames were integrated with the Bruker SAINT software package^7^ using a narrow-frame algorithm. The integration of the data using a monoclinic unit cell yielded a total of 157262 reflections to a maximum θ angle of 25.05° (0.84 Å resolution), of which 25175 were independent (average redundancy 6.247, completeness = 99.9%, R_int_ = 5.97%, R_sig_ = 3.87%) and 23807 (94.57%) were greater than 2σ(F^2^). The final cell constants of a = 19.2134(6) Å, b = 16.9966(6) Å, c = 44.5606(16) Å, β = 97.7440(10)°, volume = 14419.1(9) Å^3^, are based upon the refinement of the XYZ-centroids of 9496 reflections above 20 σ(I) with 4.670° < 2θ < 49.99°. Data were corrected for absorption effects using the Multi-Scan method (SADABS).^8^ The ratio of minimum to maximum apparent transmission was 0.720. The calculated minimum and maximum transmission coefficients (based on crystal size) are 0.9110 and 0.9410.

**Data collection**

The space group Cc was determined based on intensity statistics and systematic absences. The structure was solved and refined using the SHELX suite of programs. An intrinsic-methods solution was calculated, which provided most non-hydrogen atoms from the E-map. Full-matrix least squares/difference Fourier cycles were performed, which located the remaining non-hydrogen atoms. All non-hydrogen atoms were refined with anisotropic displacement parameters. The hydrogen atoms were placed in ideal positions and refined as riding atoms with relative isotropic displacement parameters. The structure was carefully checked for additional (missed) symmetry, instead it is twinned with a domain ratio of 67:33. Disorder was refined for some isopropyl groups and the co-crystallizing solvent using restraints and constraints. The final anisotropic full-matrix least-squares refinement on F^2^ with 1751 variables converged at R1 = 4.83%, for the observed data and wR2 = 12.70% for all data. The goodness-of-fit was 1.044. The largest peak in the final difference electron density synthesis was 0.531 e^-^/Å^3^ and the largest hole was -0.258 e^-^/Å^3^ with an RMS deviation of 0.049 e^-^/Å^3^. On the basis of the final model, the calculated density was 1.232 g/cm^3^ and F(000), 5728 e^-^.

Table S3. Crystal data and structure refinement for **3-K(dibenzo-18-c-6)**.

| Empirical formula | C_75_H_102_BFeKN_6_O_9_ | |
| --- | --- | --- |
| Formula weight | 1337.38 | |
| Crystal color, shape, size | orange block, 0.291 × 0.272 × 0.190 mm^3^ | |
| Temperature | 173(2) K | |
| Wavelength | 0.71073 Å | |
| Crystal system, space group | Monoclinic, Cc | |
| Unit cell dimensions | a = 19.2134(6) Å | α = 90° |
|  | b = 16.9966(6) Å | β = 97.7440(10)° |
|  | c = 44.5606(16) Å | γ = 90° |
| Volume | 14419.1(9) Å^3^ | |
| Z | 8 | |
| Density (calculated) | 1.232 Mg/m^3^ | |
| Absorption coefficient | 0.326 mm^-1^ | |
| F(000) | 5728 | |
| *Data collection* | | |
| Diffractometer | Venture D8, Bruker | |
| Source | Iµ3.0, Incoatec | |
| Detector | Photon III | |
| Theta range for data collection | 1.845 to 25.054° | |
| Index ranges | -22<=h<=22, -20<=k<=20, -53<=l<=53 | |
| Reflections collected | 157262 | |
| Independent reflections | 25175 [R_int_ = 0.0597] | |
| Observed Reflections | 23807 | |
| Completeness to theta = 25.087° | 99.9 % | |
| Solution and Refinement | | |
| Absorption correction | Semi-empirical from equivalents | |
| Max. and min. transmission | 0.7452 and 0.5367 | |
| Solution | Intrinsic methods | |
| Refinement method | Full-matrix least-squares on F^2^ | |
| Weighting scheme | w = [σ^2^Fo^2^ + 0.0628P^2^ + 18.9448P]^-1^, with  P = (Fo^2^ + 2 Fc^2^)/3 | |
| Data / restraints / parameters | 25175 / 597 / 1751 | |
| Goodness-of-fit on F^2^ | 1.044 | |
| Final R indices [I>2σ(I)] | R1 = 0.0483, wR2 = 0.1241 | |
| R indices (all data) | R1 = 0.0514, wR2 = 0.1270 | |
| Twin domain ratio | 67:33, inversion twin | |
| Largest diff. peak and hole | 0.531 and -0.258 e.Å^-3^ | |

**Computational Details**

All calculations were performed with the Gaussian 16 program.^9^ Geometry optimization and frequency calculations were performed in the gas phase using the ωB97X-D^10^ functional, which includes empirical dispersion corrections. This functional has shown good performance in benchmark studies^11-12^ and is widely employed in mechanistic investigations of iron-catalyzed systems^13-15^. The def-SVP^16,17^ basis set was used for all atoms. The nature of all stationary points was confirmed by the number of imaginary frequencies. All energy minima had zero imaginary frequency, and all transition states had only one imaginary frequency. Intrinsic reaction coordinate (IRC)^18,19^ calculations confirmed that all transition states are connected to the correct intermediates. To verify the reliability of this computational method, key bond lengths and angles obtained from DFT-optimized structures were compared with available X-ray crystallographic data (Tables S4 and S5). The close agreement indicates the reliability of the chosen method. Single-point energy calculations were then performed at the ωB97X-D/def2-TZVP^20^ level of theory, including solvation energy corrections for benzene and tetrahydrofuran using the SMD^21^ solvation model. For the system under investigation, the quintet state was identified as the most stable spin state (Table S6). Therefore, all calculations were focused on the quintet state. Non-covalent interaction analysis was performed using two approaches. The non-covalent interactions between the K^+^ ion (or [K(12-C-4)]^+^) and the organic moiety were characterized using the Independent Gradient Model based on Hirshfeld partition (IGMH)^22^ analysis, with the two entities treated as separate fragments. Additionally, the Interaction Region Indicator (IRI)^23^ analysis was employed to map all non-covalent interactions within the entire molecular structure of the transition states **^5^TS^α^_4_** and **^5^TS^γ^_4_**. Both analyses were performed using the Multiwfn program.^24^ The visualization of both the IGMH and IRI isosurfaces was accomplished using VMD.^25^ All computed structures were illustrated with CYLview (version 1.0).^26^

**Table S4.** Summary of key parameters in the crystal structure and DFT results of [Ph_2_B(*^t^*BuIm)_2_Fe(Py)_2_Li(THF)_2_] (**2**).

|  | | Exp. | Cal. |
| --- | --- | --- | --- |
| Bond length (Å) | Fe1-C1 | 2.12 | 2.13 |
|  | Fe1-C8 | 2.11 | 2.13 |
|  | Fe1-C27 | 2.08 | 2.13 |
|  | Fe1-C32 | 2.07 | 2.11 |
|  | Li1-N5 | 2.00 | 2.01 |
|  | Li1-N6 | 2.01 | 1.99 |
| Angle (deg) | ∠C1-Fe1-C8 | 96 | 96 |

**Table S5.** Summary of key parameters in the crystal structure and DFT results of the anionic part of [Ph_2_B(*^t^*BuIm)_2_Fe(2-Py)NHDipp][K(dibenzo-18-c-6)(THF)_2_] (**3**-K(dibenzo-18-c-6)).

|  | | Exp. | Cal. |
| --- | --- | --- | --- |
| Bond length (Å) | Fe1-C1 | 2.13 | 2.13 |
|  | Fe1-C8 | 2.13 | 2.14 |
|  | Fe1-N6 | 2.09 | 2.00 |
|  | Fe1-C27 | 2.09 | 2.09 |
|  | N6-C32 | 1.36 | 1.36 |
| Angle (deg) | ∠C1-Fe1-C8 | 96 | 96 |

**Table S6.** Relative energies (kcal/mol) of possible spin states of selected intermediates and transition states. For the **1-K(crypt)** system, energies are referenced to the quintet state of **1^-^**.

| **System** | **Species** | Quintet (*S* = 2) | Triplet (*S* = 1) | Singlet (*S* = 0) |
| --- | --- | --- | --- | --- |
| **2** | **2** | 0.0 | 16.8 | - |
| **1-K(crypt)** | **1^-^** | 0.0 | 9.7 | 32.0 |
|  | **2^-^** | 5.6 | 25.7 | - |
|  | **TS^α^_1-3_** | 21.0 | 40.0 | 56.5 |
|  | **TS^γ^_1-3_** | 24.2 | 46.7 | 67.2 |
|  | **3^-^** | -4.8 | 12.8 | - |
|  | **3^γ-^** | -6.7 | 10.3 | - |
|  | **TS^γ^_4_** | 14.6 | 36.9 | - |

The triplet state of complex **2** is calculated to lie 16.8 kcal/mol above the quintet state, and a stable singlet state could not be located computationally. These results align with experimental data confirming a high-spin (*S* = 2) ground state for **2**. For system **1-K(crypt)**, calculations indicate that the anionic complex **1^-^** is most stable in the quintet state, consistent with its high-spin ground state^27^. This quintet preference extends to intermediates **2**^-^, **3^-^** and **3^γ-^**, as well as to transition states **TS^α^_1-3_**, **TS^γ^_1-3_** and **TS^γ^_4_**, all of which are found to be most stable in the quintet state, with their triplet states being about 20 kcal/mol less stable. Therefore, all subsequent calculations were conducted on the quintet surface.

**Figure S27**. Computed Gibbs free energy profile for the deuteration of pyridine by **2** on the quintet surface. Computations were performed at the UωB97X-D/def2-TZVP/SMD(Benzene)//UωB97X-D/def2-SVP level of theory.

**Figure S28.** Alternative transition states to those presented in Figure S27, computed without isotope effects. All energies are given in kcal/mol.

**Figure S29.** Optimized structures of the transition states **^5^TS^α^_5-2_**, **^5^TS^β^_5-2_** and **^5^TS^γ^_5-2_**. Bond distances are in Å.

**Figure S30.** IRI analysis (isovalue = 1.0) of the transition states **^5^TS^α^_4_** and **^5^TS^γ^_4_**.. Bond distances are in Å.

**Figure S31.** Computed Gibbs free energy profile for the deuteration of pyridine by catalyst **1-K** on the quintet surface, employing 1-K’ (the model of 1-K without two THF ligands) as the computational starting point. Computations were performed at the same level as for **2**, with THF as solvent.

**Figure S32.** Optimized structures and IGMH analysis (isovalue = 0.008) of the transition states **^5^TS_K_^α^_1-2_**, **^5^TS_K_^β^_1-2_** and **^5^TS_K_^γ^_1-2_**. Bond distances are in Å.

**Figure S33.** Optimized structures and IGMH analysis (isovalue = 0.008) of the transition states **^5^TS_K_^α^_2-3,_ ^5^TS_K_^β^_2-3_** and **^5^TS_K_^γ^_2-3_**. Bond distances are in Å.

**Energy tables for the stationary points discussed in Scheme 3, Figure 3-5 and Figure S24-30.**

**Table S7** The UωB97X-D/def2-SVP computed electronic energy, thermal energies, enthalpies and free energies of all stationary points at 298.15K discussed in Scheme 3 and Figure S24-S26.

| **species** | charge ◊ spin | E0 | E0+ZPE | U | H | G |
| --- | --- | --- | --- | --- | --- | --- |
| **2** | (0 ◊ 3) | -3483.219092 | -3482.277822 | -3482.223357 | -3482.222413 | -3482.369634 |
| **2** | (0 ◊ 5) | -3483.240175 | -3482.299661 | -3482.244758 | -3482.243814 | -3482.393962 |
| **5** | (0 ◊ 5) | -3216.582997 | -3215.705191 | -3215.654664 | -3215.653720 | -3215.791569 |
| **TS_2-5_** | (0 ◊ 5) | -3464.590685 | -3463.627977 | -3463.571880 | -3463.570936 | -3463.721713 |
| **TS_2-5_^’^** | (0 ◊ 5) | -3464.587968 | -3463.618948 | -3463.563339 | -3463.562395 | -3463.711133 |
| **TS_2-5_^’-2^** | (0 ◊ 5) | -3232.345785 | -3231.497311 | -3231.447658 | -3231.446714 | -3231.582433 |
| **TS_2-5_^’-3^** | (0 ◊ 5) | -3696.826139 | -3695.738223 | -3695.676247 | -3695.675303 | -3695.839146 |
| **TS_2-5_^’-4^** | (0 ◊ 5) | -3464.559218 | -3463.592886 | -3463.536437 | -3463.535493 | -3463.688752 |
| **TS_2-5_^’-5^** | (0 ◊ 5) | -3696.796595 | -3695.710301 | -3695.647844 | -3695.646899 | -3695.812608 |
| **TS_2-5_^’-6^** | (0 ◊ 5) | -3232.317089 | -3231.469973 | -3231.419872 | -3231.418927 | -3231.555960 |
| **TS^α^_5-2_** | (0 ◊ 5) | -3464.590685 | -3463.625912 | -3463.569871 | -3463.568927 | -3463.719612 |
| **TS^β^_5-2_** | (0 ◊ 5) | -3464.569869 | -3463.605224 | -3463.549274 | -3463.548330 | -3463.699450 |
| **TS^γ^_5-2_** | (0 ◊ 5) | -3464.575088 | -3463.610952 | -3463.554744 | -3463.553799 | -3463.705261 |
| **Pyridine** | (0 ◊ 1) | -248.016503 | -247.926808 | -247.922574 | -247.921630 | -247.954189 |
| ***α*-deuterated pyridine** | (0 ◊ 1) | -248.015509 | -247.929967 | -247.925675 | -247.924731 | -247.957442 |
| **THF** | (0 ◊ 1) | -232.210360 | -232.092641 | -232.087807 | -232.086863 | -232.120674 |
| ***^t^*BuNHD** | (0 ◊ 1) | -213.591127 | -213.445275 | -213.438460 | -213.437515 | -213.474317 |
| ***^t^*BuND_2_** | (0 ◊ 1) | -213.591127 | -213.448714 | -213.441758 | -213.440814 | -213.477923 |

E0 is the electronic energy calculated using UωB97X-D/def2-SVP method. ZPE is the zero-point vibrational energy. U, H and G are the thermal energy, enthalpy and Gibbs free energy obtained by adding the thermal corrections from frequency calculations to E0. The unit of all energies in Table S7 is Hartree.

**Table S8** The UωB97X-D/def2-TZVP/SMD(Benzene)//UωB97X-D/def2-SVP computed electronic energy, thermal energies, enthalpies and free energies of all stationary points at 298.15K discussed in Scheme 3 and Figure S24-S26.

| **species** | charge ◊ spin | E0 | E0+ZPE | U | H | G |
| --- | --- | --- | --- | --- | --- | --- |
| **2** | (0 ◊ 3) | -3485.762478 | -3484.821208 | -3484.766744 | -3484.765799 | -3484.91302 |
| **2** | (0 ◊ 5) | -3485.785966 | -3484.845452 | -3484.790549 | -3484.789605 | -3484.939753 |
| **5** | (0 ◊ 5) | -3218.837872 | -3217.960066 | -3217.909539 | -3217.908595 | -3218.046444 |
| **TS_2-5_** | (0 ◊ 5) | -3467.105603 | -3466.142895 | -3466.086797 | -3466.085853 | -3466.23663 |
| **TS_2-5_^，^** | (0 ◊ 5) | -3467.105603 | -3466.136584 | -3466.080975 | -3466.08003 | -3466.228768 |
| **TS_2-5_^’-2^** | (0 ◊ 5) | -3234.608327 | -3233.759853 | -3233.7102 | -3233.709256 | -3233.844975 |
| **TS_2-5_^’-3^** | (0 ◊ 5) | -3699.595516 | -3698.5076 | -3698.445623 | -3698.444679 | -3698.608523 |
| **TS_2-5_^’-4^** | (0 ◊ 5) | -3467.080726 | -3466.114394 | -3466.057944 | -3466.057 | -3466.210259 |
| **TS_2-5_^’-5^** | (0 ◊ 5) | -3699.570815 | -3698.484521 | -3698.422064 | -3698.42112 | -3698.586828 |
| **TS_2-5_^’-6^** | (0 ◊ 5) | -3234.58028 | -3233.733163 | -3233.683062 | -3233.682118 | -3233.819151 |
| **TS^α^_5-2_** | (0 ◊ 5) | -3467.105603 | -3466.140829 | -3466.084788 | -3466.083844 | -3466.234529 |
| **TS^β^_5-2_** | (0 ◊ 5) | -3467.089896 | -3466.125252 | -3466.069302 | -3466.068358 | -3466.219478 |
| **TS^γ^_5-2_** | (0 ◊ 5) | -3467.093937 | -3466.129801 | -3466.073593 | -3466.072648 | -3466.22411 |
| **Pyridine** | (0 ◊ 1) | -248.29077 | -248.201076 | -248.196842 | -248.195897 | -248.228456 |
| ***α*-deuterated pyridine** | (0 ◊ 1) | -248.29077 | -248.205228 | -248.200936 | -248.199992 | -248.232702 |
| **THF** | (0 ◊ 1) | -232.472653 | -232.354934 | -232.350101 | -232.349157 | -232.382967 |
| ***^t^*BuNHD** | (0 ◊ 1) | -213.829163 | -213.683311 | -213.676495 | -213.675551 | -213.712353 |
| ***^t^*BuND_2_** | (0 ◊ 1) | -213.829163 | -213.68675 | -213.679794 | -213.67885 | -213.715959 |

E0 is the electronic energy calculated using UωB97X-D/def2-TZVP/SMD(Benzene) method based on geometries obtained by UωB97X-D/def2-SVP method. U, H and G are the thermal energy, enthalpy and Gibbs free energy obtained by adding the thermal corrections in Table S7 to E0. The unit of all energies in Table S8 is Hartree.

**Table S9** The UωB97X-D/def2-SVP computed electronic energy, thermal energies, enthalpies and free energies of all stationary points at 323.15K discussed in Figure 3-5 and Figure S27-S30.

| **species** | charge ◊ spin | E0 | E0+ZPE | U | H | G |
| --- | --- | --- | --- | --- | --- | --- |
| **1^-^** | (-1 ◊ 1) | -3038.072791 | -3037.266922 | -3037.215392 | -3037.214369 | -3037.353194 |
| **1^-^** | (-1 ◊ 3) | -3038.101982 | -3037.296986 | -3037.245019 | -3037.243995 | -3037.386493 |
| **1^-^** | (-1 ◊ 5) | -3038.114735 | -3037.310766 | -3037.258443 | -3037.257420 | -3037.401528 |
| **2^-^** | (-1 ◊ 3) | -3286.134805 | -3285.238142 | -3285.179835 | -3285.178811 | -3285.334097 |
| **2^-^** | (-1 ◊ 5) | -3286.161039 | -3285.265997 | -3285.207175 | -3285.206151 | -3285.363790 |
| **3^-^** | (-1 ◊ 3) | -3286.161306 | -3285.264882 | -3285.206278 | -3285.205255 | -3285.361401 |
| **3^-^** | (-1 ◊ 5) | -3286.184964 | -3285.288427 | -3285.229655 | -3285.228631 | -3285.386859 |
| **3^γ-^** | (-1 ◊ 3) | -3286.163765 | -3285.266726 | -3285.208180 | -3285.207156 | -3285.362898 |
| **3^γ-^** | (-1 ◊ 5) | -3286.190795 | -3285.293885 | -3285.235115 | -3285.234091 | -3285.391801 |
| **1-K^’^** | (0 ◊ 5) | -3637.982349 | -3637.175307 | -3637.121133 | -3637.120110 | -3637.266749 |
| **2-K^α^** | (0 ◊ 5) | -3886.035131 | -3885.135660 | -3885.075032 | -3885.074009 | -3885.234964 |
| **2-K^β^** | (0 ◊ 5) | -3886.021244 | -3885.121083 | -3885.060739 | -3885.059716 | -3885.219561 |
| **2-K^γ^** | (0 ◊ 5) | -3886.020511 | -3885.120530 | -3885.060197 | -3885.059174 | -3885.218843 |
| **3-K** | (0 ◊ 5) | -3851.618082 | -3850.662028 | -3850.599233 | -3850.598210 | -3850.761201 |
| **TS^α^_1-3_** | (-1 ◊ 1) | -3286.092576 | -3285.199978 | -3285.142579 | -3285.141556 | -3285.293543 |
| **TS^α^_1-3_** | (-1 ◊ 3) | -3286.114582 | -3285.223168 | -3285.165016 | -3285.163993 | -3285.319552 |
| **TS^α^_1-3_** | (-1 ◊ 5) | -3286.141342 | -3285.250117 | -3285.191982 | -3285.190959 | -3285.345868 |
| **TS^γ^_1-3_** | (-1 ◊ 1) | -3286.063881 | -3285.172004 | -3285.113899 | -3285.112876 | -3285.267596 |
| **TS^γ^_1-3_** | (-1 ◊ 3) | -3286.096061 | -3285.204117 | -3285.145940 | -3285.144917 | -3285.301122 |
| **TS^γ^_1-3_** | (-1 ◊ 5) | -3286.115319 | -3285.225055 | -3285.166232 | -3285.165209 | -3285.326192 |
| **TS^α^_4_** | (-1 ◊ 5) | -3499.748949 | -3498.708451 | -3498.641719 | -3498.640696 | -3498.811810 |
| **TS^γ^_4_** | (-1 ◊ 3) | -3499.749613 | -3498.710368 | -3498.643398 | -3498.642374 | -3498.814855 |
| **TS^γ^_4_** | (-1 ◊ 5) | -3499.783963 | -3498.743892 | -3498.677257 | -3498.676234 | -3498.847782 |
| **TS_K_^α^_1-2_** | (0 ◊ 5) | -3885.995560 | -3885.101365 | -3885.040970 | -3885.039947 | -3885.201360 |
| **TS_K_^β^_1-2_** | (0 ◊ 5) | -3885.985672 | -3885.093014 | -3885.032018 | -3885.030994 | -3885.195012 |
| **TS_K_^γ^_1-2_** | (0 ◊ 5) | -3885.986135 | -3885.093506 | -3885.032648 | -3885.031625 | -3885.195038 |
| **TS_K_^α^_2-3_** | (0 ◊ 5) | -4099.629043 | -4098.587389 | -4098.518386 | -4098.517362 | -4098.696263 |
| **TS_K_^β^_2-3_** | (0 ◊ 5) | -4099.633549 | -4098.591271 | -4098.522360 | -4098.521336 | -4098.697431 |
| **TS_K_^γ^_2-3_** | (0 ◊ 5) | -4099.626752 | -4098.584697 | -4098.515818 | -4098.514794 | -4098.690529 |
| **TS_K(12-C-4)_^γ^_1-3_** | (0 ◊ 5) | -4500.691012 | -4499.547166 | -4499.471283 | -4499.470260 | -4499.665669 |
| **TS_K(12-C-4)_^γ^_4_** | (0 ◊ 5) | -4714.367640 | -4713.073495 | -4712.990005 | -4712.988982 | -4713.194705 |
| **Pyridine** | (0 ◊ 1) | -248.016503 | -247.926808 | -247.921908 | -247.920885 | -247.956949 |
| **α-deuterated pyridine** | (0 ◊ 1) | -248.015509 | -247.929967 | -247.924987 | -247.923964 | -247.960215 |
| **β-deuterated pyridine** | (0 ◊ 1) | -248.015509 | -247.929928 | -247.924936 | -247.923913 | -247.960181 |
| **γ-deuterated pyridine** | (0 ◊ 1) | -248.015509 | -247.929933 | -247.924945 | -247.923921 | -247.960186 |
| ***^t^*BuND_2_** | (0 ◊ 1) | -213.591127 | -213.448714 | -213.440669 | -213.439646 | -213.481082 |

E0 is the electronic energy calculated using UωB97X-D/def2-SVP method. ZPE is the zero-point vibrational energy. U, H and G are the thermal energy, enthalpy and Gibbs free energy obtained by adding the thermal corrections from frequency calculations to E0. The unit of all energies in Table S9 is Hartree.

**Table S10** The UωB97X-D/def2-TZVP/SMD(THF)//UωB97X-D/def2-SVP computed electronic energy, thermal energies, enthalpies and free energies of all stationary points at 323.15K discussed in Figure 3-5 and Figure S26-S30.

| **species** | charge ◊ spin | E0 | E0+ZPE | U | H | G |
| --- | --- | --- | --- | --- | --- | --- |
| **1^-^** | (-1 ◊ 1) | -3040.182181 | -3039.376312 | -3039.324782 | -3039.323759 | -3039.462584 |
| **1^-^** | (-1 ◊ 3) | -3040.213687 | -3039.408691 | -3039.356723 | -3039.3557 | -3039.498197 |
| **1^-^** | (-1 ◊ 5) | -3040.226865 | -3039.4229 | -3039.37057 | -3039.36955 | -3039.51366 |
| **2^-^** | (-1 ◊ 3) | -3288.505814 | -3287.609151 | -3287.550844 | -3287.54982 | -3287.705106 |
| **2^-^** | (-1 ◊ 5) | -3288.534347 | -3287.63931 | -3287.58048 | -3287.57946 | -3287.7371 |
| **3^-^** | (-1 ◊ 3) | -3288.526049 | -3287.629625 | -3287.571022 | -3287.569998 | -3287.726144 |
| **3^-^** | (-1 ◊ 5) | -3288.551854 | -3287.65532 | -3287.59655 | -3287.59552 | -3287.75375 |
| **3^γ-^** | (-1 ◊ 3) | -3288.530534 | -3287.633494 | -3287.574948 | -3287.573925 | -3287.729667 |
| **3^γ-^** | (-1 ◊ 5) | -3288.555809 | -3287.658899 | -3287.600128 | -3287.599105 | -3287.756814 |
| **1-K’** | (0 ◊ 5) | -3640.12781 | -3639.32077 | -3639.2666 | -3639.26557 | -3639.41221 |
| **2-K^α^** | (0 ◊ 5) | -3888.43966 | -3887.54019 | -3887.47956 | -3887.47854 | -3887.63949 |
| **2-K^β^** | (0 ◊ 5) | -3888.4334 | -3887.53324 | -3887.4729 | -3887.47188 | -3887.63172 |
| **2-K^γ^** | (0 ◊ 5) | -3888.43425 | -3887.53427 | -3887.47394 | -3887.47291 | -3887.63258 |
| **3-K** | (0 ◊ 5) | -3853.98726 | -3853.0312 | -3852.96841 | -3852.96738 | -3853.13038 |
| **TS^α^_1-3_** | (-1 ◊ 1) | -3288.455008 | -3287.56241 | -3287.505011 | -3287.503988 | -3287.655975 |
| **TS^α^_1-3_** | (-1 ◊ 3) | -3288.477302 | -3287.585888 | -3287.527737 | -3287.526713 | -3287.682273 |
| **TS^α^_1-3_** | (-1 ◊ 5) | -3288.508109 | -3287.61688 | -3287.55875 | -3287.55773 | -3287.71264 |
| **TS^γ^_1-3_** | (-1 ◊ 1) | -3288.435201 | -3287.543324 | -3287.48522 | -3287.484196 | -3287.638917 |
| **TS^γ^_1-3_** | (-1 ◊ 3) | -3288.466565 | -3287.574621 | -3287.516444 | -3287.515421 | -3287.671626 |
| **TS^γ^_1-3_** | (-1 ◊ 5) | -3288.496561 | -3287.6063 | -3287.54747 | -3287.54645 | -3287.70743 |
| **TS^α^_4_** | (-1 ◊ 5) | -3502.346202 | -3501.3057 | -3501.23897 | -3501.23795 | -3501.40906 |
| **TS^γ^_4_** | (-1 ◊ 3) | -3502.342690 | -3501.303444 | -3501.236474 | -3501.235451 | -3501.407931 |
| **TS^γ^_4_** | (-1 ◊ 5) | -3502.379695 | -3501.33962 | -3501.27299 | -3501.27197 | -3501.44351 |
| **TS_K_^α^_1-2_** | (0 ◊ 5) | -3888.40308 | -3887.50889 | -3887.44849 | -3887.44747 | -3887.60888 |
| **TS_K_^β^_1-2_** | (0 ◊ 5) | -3888.39834 | -3887.50568 | -3887.44469 | -3887.44366 | -3887.60768 |
| **TS_K_^γ^_1-2_** | (0 ◊ 5) | -3888.39805 | -3887.50542 | -3887.44456 | -3887.44354 | -3887.60695 |
| **TS_K_^α^_2-3_** | (0 ◊ 5) | -4102.25992 | -4101.21826 | -4101.14926 | -4101.14824 | -4101.32714 |
| **TS_K_^β^_2-3_** | (0 ◊ 5) | -4102.26419 | -4101.22191 | -4101.153 | -4101.15198 | -4101.32807 |
| **TS_K_^γ^_2-3_** | (0 ◊ 5) | -4102.26098 | -4101.21892 | -4101.15004 | -4101.14902 | -4101.32475 |
| **TS_K(12-C-4)_^γ^_1-3_** | (0 ◊ 5) | -4503.795203 | -4502.651357 | -4502.575474 | -4502.574451 | -4502.769859 |
| **TS_K(12-C-4)_^γ^_4_** | (0 ◊ 5) | -4717.68485 | -4716.390704 | -4716.307215 | -4716.306192 | -4716.511915 |
| **Pyridine** | (0 ◊ 1) | -248.291964 | -248.20227 | -248.19737 | -248.196346 | -248.23241 |
| **α-deuterated pyridine** | (0 ◊ 1) | -248.291964 | -248.206422 | -248.201442 | -248.200418 | -248.23667 |
| **β-deuterated pyridine** | (0 ◊ 1) | -248.291964 | -248.206382 | -248.201391 | -248.200368 | -248.236636 |
| **γ-deuterated pyridine** | (0 ◊ 1) | -248.291964 | -248.206387 | -248.201399 | -248.200376 | -248.23664 |
| ***^t^*BuND_2_** | (0 ◊ 1) | -213.830751 | -213.688338 | -213.680293 | -213.67927 | -213.720705 |

E0 is the electronic energy calculated using UωB97X-D/def2-TZVP/SMD(THF) method based on geometries obtained by UωB97X-D/def2-SVP method. U, H and G are the thermal energy, enthalpy and Gibbs free energy obtained by adding the thermal corrections in Table S9 to E0. The unit of all energies in Table S10 is Hartree.

**Cartesian Coordinates for the stationary points discussed in Scheme 3, Figure 3-5 and Figure S24-S30.** For transition state structurs, only one imaginary frequency was observed and given below. For all stable structures, no imaginary frequency was observed.

**The Cartesian Coordinates of the stationary points discussed in Scheme 3 and Figure S24-S26.**

**2** (triplet)

Atom X Y Z

Fe -0.074201 0.063243 0.001709

B -3.355496 -0.008492 0.127832

C -1.345509 -1.541144 -0.433193

Li 3.174894 0.183016 -0.145123

N -2.668123 -1.266253 -0.545986

N -1.196461 -2.817162 -0.888658

C -3.334124 -2.349856 -1.072768

H -4.407404 -2.356874 -1.231566

C -2.418358 -3.323555 -1.299249

H -2.550516 -4.321156 -1.701498

N -2.721969 1.328877 -0.429255

C 0.066142 -3.587998 -0.944464

N -1.306688 2.946846 -0.696498

C -0.240508 -5.074143 -0.709870

H -0.801609 -5.218708 0.225233

H 0.705115 -5.629979 -0.633704

H -0.811433 -5.522515 -1.535416

N 2.141564 0.250131 -1.837981

C 0.718748 -3.404061 -2.319633

H 0.032372 -3.709785 -3.123777

H 1.628335 -4.020960 -2.392591

H 0.990748 -2.353167 -2.483242

N 2.520787 0.583359 1.691749

C 1.009122 -3.112086 0.158204

H 1.357918 -2.084659 -0.008662

H 1.884946 -3.776118 0.183228

H 0.518249 -3.143222 1.141794

C -1.400868 1.626703 -0.367605

C -3.442446 2.444394 -0.792849

H -4.523517 2.438762 -0.883637

C -2.563153 3.459898 -0.971825

H -2.741460 4.489445 -1.258631

C -0.063905 3.750102 -0.777367

C -0.384073 5.212613 -0.438108

H -1.009296 5.696117 -1.202270

H 0.554710 5.781937 -0.383446

H -0.891287 5.291895 0.534881

C 0.504540 3.660704 -2.198755

H 0.775205 2.626216 -2.445441

H 1.403473 4.290214 -2.288693

H -0.233715 4.008612 -2.937226

C 0.953497 3.233274 0.237373

H 0.525145 3.192411 1.249217

H 1.821130 3.909219 0.251220

H 1.304245 2.225070 -0.012615

C -4.946789 -0.022539 -0.230357

C -5.943237 -0.089008 0.751875

H -5.651796 -0.134706 1.804649

C -7.301783 -0.097551 0.423830

H -8.052053 -0.150018 1.217103

C -7.701386 -0.038831 -0.908814

H -8.762827 -0.045535 -1.168903

C -6.730753 0.030299 -1.910237

H -7.030145 0.078696 -2.960578

C -5.380353 0.038648 -1.568260

H -4.633853 0.096397 -2.367590

C -3.064879 -0.091605 1.732219

C -3.069820 -1.326994 2.402013

H -3.241643 -2.243732 1.828939

C -2.853169 -1.423592 3.776105

H -2.861935 -2.402464 4.262901

C -2.620279 -0.271532 4.529014

H -2.446332 -0.340565 5.605923

C -2.612550 0.967580 3.891320

H -2.426741 1.877457 4.467793

C -2.835645 1.049142 2.515805

H -2.821130 2.033348 2.038325

C 0.802750 0.131931 -1.692495

C 0.009626 0.085982 -2.874080

H -1.074714 -0.004937 -2.776390

C 0.588496 0.157884 -4.126885

H -0.031497 0.122348 -5.027029

C 1.982405 0.280903 -4.231732

H 2.483616 0.344115 -5.198901

C 2.706864 0.323303 -3.054281

H 3.798638 0.424784 -3.065424

C 1.262956 0.081458 1.552034

C 0.584152 -0.314942 2.731926

H -0.420477 -0.744494 2.654537

C 1.151476 -0.156316 3.987552

H 0.598871 -0.450640 4.884210

C 2.435904 0.388203 4.083871

H 2.928258 0.538307 5.046473

C 3.075029 0.728578 2.901245

H 4.092501 1.140361 2.925095

O 4.011437 -1.564921 0.082655

C 4.249023 -2.271248 1.292660

H 3.292035 -2.640170 1.699051

H 4.676126 -1.574071 2.028100

C 5.188982 -3.406687 0.905216

H 5.120252 -4.264947 1.587355

H 6.232860 -3.054811 0.905793

C 4.733889 -3.724544 -0.521085

H 5.510781 -4.208327 -1.128537

H 3.859564 -4.393082 -0.504528

C 4.345896 -2.347489 -1.058656

H 5.183247 -1.858347 -1.584879

H 3.480432 -2.370828 -1.737615

O 4.816411 1.255458 -0.653061

C 5.817446 1.166487 0.356715

H 6.767146 0.830418 -0.090708

H 5.492397 0.402206 1.079184

C 5.930076 2.563827 0.986083

H 6.816182 3.090909 0.600495

H 6.020030 2.524011 2.080606

C 4.648650 3.257121 0.514471

H 3.803307 2.999168 1.169909

H 4.737403 4.351283 0.466678

C 4.441473 2.613120 -0.847372

H 3.401417 2.623306 -1.201353

H 5.082852 3.077403 -1.619970

**2** (quintet)

Atom X Y Z

Fe 0.049353 0.000272 -0.007112

B 3.462482 -0.023817 -0.082755

C 1.424389 1.572395 -0.417778

Li -3.154205 -0.122225 0.306739

N 2.740781 1.297856 -0.571940

N 1.295388 2.906534 -0.666609

C 3.423829 2.442169 -0.916574

H 4.497721 2.455238 -1.071474

C 2.524291 3.454464 -0.988932

H 2.672100 4.499624 -1.234155

N 2.712882 -1.313279 -0.604557

C 0.030308 3.674267 -0.658466

N 1.260546 -2.916938 -0.699123

C 0.320643 5.126372 -0.256124

H 0.847100 5.170543 0.708730

H -0.629807 5.669747 -0.154994

H 0.918140 5.660667 -1.008334

N -2.550804 -0.557893 -1.560102

C -0.577784 3.630789 -2.064856

H 0.115842 4.059269 -2.804171

H -1.514288 4.209470 -2.097918

H -0.793040 2.592323 -2.353627

N -2.171057 -0.202129 2.037492

C -0.937730 3.075905 0.363165

H -1.316386 2.093309 0.051213

H -1.801542 3.748156 0.470550

H -0.460524 2.957395 1.346028

C 1.404814 -1.597050 -0.387789

C 3.371053 -2.435056 -1.056789

H 4.432784 -2.439392 -1.279899

C 2.466872 -3.442435 -1.126810

H 2.597648 -4.472668 -1.436727

C 0.011885 -3.712216 -0.632827

C 0.355695 -5.149232 -0.214316

H 0.939782 -5.683692 -0.976770

H -0.575179 -5.715249 -0.066902

H 0.919062 -5.158998 0.730563

C -0.651050 -3.711230 -2.015444

H -0.889011 -2.686004 -2.327283

H -1.583563 -4.296010 -1.993123

H 0.016489 -4.155961 -2.769299

C -0.932735 -3.124639 0.415532

H -0.423292 -2.979217 1.378422

H -1.774576 -3.817405 0.563219

H -1.345053 -2.158969 0.100586

C 4.977547 -0.027284 -0.692697

C 6.121514 -0.081907 0.113450

H 6.006124 -0.121440 1.200038

C 7.407767 -0.087208 -0.433776

H 8.278890 -0.130511 0.225222

C 7.581653 -0.037268 -1.814516

H 8.585806 -0.041090 -2.245924

C 6.458810 0.017900 -2.643200

H 6.581406 0.057240 -3.728897

C 5.183109 0.022572 -2.084075

H 4.313173 0.065621 -2.747967

C 3.444439 -0.040280 1.549775

C 3.503795 1.145651 2.298785

H 3.518783 2.108681 1.779090

C 3.534701 1.138739 3.693783

H 3.575329 2.083208 4.242839

C 3.506252 -0.070647 4.387839

H 3.526687 -0.082471 5.480516

C 3.447858 -1.264572 3.669488

H 3.419057 -2.220146 4.199636

C 3.419120 -1.241998 2.274808

H 3.366896 -2.192693 1.735483

C -1.258030 -0.142426 -1.681128

C -0.740117 -0.022279 -2.993656

H 0.297270 0.304483 -3.116570

C -1.501891 -0.326707 -4.114069

H -1.081967 -0.233758 -5.119786

C -2.815681 -0.769288 -3.935960

H -3.454389 -1.032375 -4.781079

C -3.286969 -0.861511 -2.634292

H -4.314759 -1.193978 -2.440964

C -0.822940 -0.052597 1.908612

C -0.058575 0.015061 3.094815

H 1.024641 0.131381 3.019885

C -0.652770 -0.070952 4.348035

H -0.041915 -0.020132 5.254087

C -2.038445 -0.228357 4.431956

H -2.552514 -0.306182 5.392182

C -2.749301 -0.287031 3.241071

H -3.839435 -0.414755 3.241811

O -3.968652 1.632618 0.031838

C -4.464246 2.387603 1.128960

H -3.616041 2.778674 1.716820

H -5.046079 1.716299 1.777638

C -5.287812 3.505237 0.499157

H -5.360079 4.393150 1.141767

H -6.310724 3.155030 0.287252

C -4.528868 3.755356 -0.806066

H -5.141076 4.229592 -1.585104

H -3.657380 4.403243 -0.623749

C -4.076177 2.349651 -1.192560

H -4.816488 1.845223 -1.838799

H -3.105615 2.322040 -1.707597

O -4.829241 -1.142172 0.669717

C -6.064962 -0.833050 0.038743

H -5.892535 -0.053528 -0.718971

H -6.760455 -0.427568 0.794589

C -6.577278 -2.149016 -0.544435

H -6.200932 -2.289642 -1.570085

H -7.674134 -2.196739 -0.579983

C -5.946795 -3.180360 0.392771

H -6.529741 -3.264244 1.323521

H -5.865485 -4.182293 -0.049993

C -4.589964 -2.547083 0.669067

H -4.147810 -2.822796 1.637015

H -3.860235 -2.786362 -0.124714

**5**

Atom X Y Z

Fe -0.495841 0.350825 -0.016932

N 2.255328 0.578141 -1.208957

N 1.257507 2.400567 -1.829935

N 1.714942 -1.676186 0.036973

N 0.036827 -2.718013 0.928334

N -2.670141 1.574502 1.650502

N -2.094946 0.079926 -1.301898

H -2.256276 1.019183 -1.669680

C 0.438940 -1.500005 0.459417

C 2.102443 -2.982924 0.233829

H 3.092516 -3.343222 -0.025109

C 1.059259 -3.642008 0.792953

H 0.974820 -4.676917 1.106560

C -1.175831 -3.029616 1.719902

C -1.792299 -4.339974 1.212108

H -2.710866 -4.557955 1.776534

H -1.118797 -5.198561 1.342250

H -2.049244 -4.261195 0.145820

C -0.751313 -3.158783 3.189938

H -1.624494 -3.372107 3.824516

H -0.291144 -2.219058 3.529918

H -0.021057 -3.969762 3.327325

C -2.213407 -1.918891 1.598717

H -3.110518 -2.206353 2.166495

H -2.485666 -1.775770 0.544499

H -1.840744 -0.971360 2.007975

C 1.108371 1.282482 -1.063077

C 3.108465 1.240765 -2.063092

H 4.091209 0.858908 -2.319463

C 2.493608 2.383477 -2.455214

H 2.840271 3.161216 -3.124742

C 0.414553 3.618949 -1.730919

C 0.767221 4.318957 -0.411564

H 0.187961 5.248258 -0.300365

H 1.838540 4.569504 -0.381458

H 0.541180 3.659937 0.438855

C -1.069318 3.258971 -1.746448

H -1.665807 4.182912 -1.755101

H -1.348825 2.693155 -0.848629

H -1.320975 2.671925 -2.641911

C 0.704877 4.546076 -2.916231

H 0.012561 5.398837 -2.881443

H 0.559435 4.026752 -3.875556

H 1.724044 4.956576 -2.886212

C 4.153580 -1.134831 -0.704973

C 5.352278 -1.056783 0.014846

H 5.360790 -0.545356 0.981326

C 6.538033 -1.615399 -0.470174

H 7.456692 -1.536560 0.116998

C 6.551726 -2.271182 -1.698741

H 7.477197 -2.708767 -2.081328

C 5.370619 -2.363864 -2.438196

H 5.368602 -2.875704 -3.404197

C 4.195691 -1.802765 -1.942835

H 3.277999 -1.883567 -2.535213

C 2.904401 0.269283 1.278954

C 2.686714 -0.387882 2.500205

H 2.409796 -1.446799 2.500065

C 2.802203 0.268365 3.726346

H 2.615858 -0.276978 4.655374

C 3.150096 1.617756 3.768284

H 3.238015 2.137659 4.725483

C 3.384756 2.294453 2.571564

H 3.659208 3.352649 2.586128

C 3.262852 1.625230 1.353473

H 3.446127 2.184310 0.430640

C -1.310491 1.463768 1.579497

C -0.542177 2.123644 2.562742

H 0.547690 2.028492 2.532525

C -1.144290 2.879575 3.561808

H -0.538347 3.385036 4.319259

C -2.537145 2.982172 3.585995

H -3.056560 3.564971 4.349125

C -3.253199 2.304167 2.608359

H -4.348858 2.341115 2.596348

C -2.048326 -0.796356 -2.476582

B 2.756261 -0.498323 -0.161736

Li -3.275537 0.236595 0.274268

C -0.710349 -0.667564 -3.231544

H -0.529803 0.379838 -3.521684

H 0.127711 -0.976233 -2.587849

H -0.690069 -1.285823 -4.144360

C -3.189737 -0.436966 -3.442233

H -4.164162 -0.531814 -2.938322

H -3.090051 0.605717 -3.788426

H -3.199464 -1.084888 -4.333652

C -2.215244 -2.254217 -2.037130

H -2.190086 -2.940393 -2.898262

H -1.405166 -2.541095 -1.351224

H -3.177388 -2.394143 -1.516872

O -5.137829 -0.359421 0.203198

C -5.629839 -1.692464 0.142977

H -6.363660 -1.854051 0.953173

H -4.785133 -2.378406 0.296985

C -6.275238 -1.812064 -1.229097

H -7.030298 -2.608456 -1.275136

H -5.507658 -2.023540 -1.988910

C -6.861247 -0.411452 -1.422674

H -6.981711 -0.137253 -2.479499

H -7.849332 -0.340333 -0.942863

C -5.849766 0.486501 -0.704375

H -5.115566 0.924042 -1.401578

H -6.326118 1.303014 -0.140647

**^5^TS_2-5_**

Imaginary frequency: -798.81 cm^-1^

Atom X Y Z

Fe -0.194287 0.122047 0.084259

N 2.511849 0.950366 -0.963033

N 1.265985 2.710248 -1.188547

N 2.287376 -1.564193 -0.244255

N 0.768257 -2.941892 0.458291

N -2.264025 0.802588 2.347079

N -1.689694 -0.180439 -1.414499

H -2.124657 0.741309 -1.496551

C 1.016544 -1.623361 0.226047

C 2.818214 -2.833382 -0.320455

H 3.831793 -3.020601 -0.659251

C 1.870900 -3.701166 0.110139

H 1.899871 -4.782008 0.199747

C -0.418476 -3.551681 1.107666

C -1.117438 -4.482569 0.111583

H -1.968398 -4.981166 0.598178

H -0.436218 -5.259382 -0.266742

H -1.504018 -3.910112 -0.741044

C 0.061632 -4.334842 2.338499

H -0.806574 -4.725042 2.889258

H 0.632661 -3.676893 3.010264

H 0.696793 -5.190783 2.068582

C -1.383978 -2.469308 1.566792

H -2.279288 -2.939862 1.996897

H -1.715601 -1.865735 0.713263

H -0.930255 -1.812663 2.319818

C 1.297649 1.459041 -0.648088

C 3.227861 1.867277 -1.700562

H 4.233189 1.669377 -2.057311

C 2.458020 2.973113 -1.843121

H 2.671719 3.903025 -2.355465

C 0.251497 3.748158 -0.884273

C 0.499083 4.241872 0.546783

H -0.212034 5.040536 0.808229

H 1.520598 4.638901 0.646259

H 0.378077 3.415912 1.261310

C -1.157798 3.173479 -1.000355

H -1.887277 3.978383 -0.830993

H -1.338140 2.396497 -0.245932

H -1.334386 2.754115 -2.001102

C 0.385394 4.910739 -1.873552

H -0.427879 5.627806 -1.693794

H 0.309575 4.562660 -2.914819

H 1.330862 5.457483 -1.750111

C 4.594567 -0.614298 -0.983442

C 5.841052 -0.552404 -0.348542

H 5.889010 -0.257900 0.703481

C 7.025224 -0.858407 -1.025011

H 7.982306 -0.800688 -0.500140

C 6.988147 -1.236838 -2.364756

H 7.912053 -1.476830 -2.896791

C 5.758213 -1.307625 -3.022622

H 5.716362 -1.604245 -4.073944

C 4.585659 -1.000402 -2.336454

H 3.628989 -1.061780 -2.866228

C 3.390639 0.177731 1.341686

C 3.261716 -0.730629 2.403463

H 3.041223 -1.781128 2.191278

C 3.387588 -0.334369 3.735570

H 3.267167 -1.069581 4.535457

C 3.658305 0.996838 4.047940

H 3.753754 1.312739 5.089826

C 3.809178 1.919541 3.012735

H 4.024177 2.966977 3.240408

C 3.677625 1.509098 1.686103

H 3.787294 2.257321 0.894762

C -0.978381 0.831114 1.902215

C 0.019906 1.248185 2.815943

H 1.064422 1.274174 2.491864

C -0.294318 1.610830 4.117990

H 0.495811 1.916796 4.809366

C -1.630181 1.574771 4.528031

H -1.929997 1.852020 5.540289

C -2.573798 1.161923 3.600082

H -3.634416 1.108954 3.872870

C -1.300940 -0.583448 -2.789916

B 3.198250 -0.269764 -0.220991

Li -3.326207 0.069026 0.859093

C -3.862963 -1.775101 -0.732042

C -4.362027 -2.872312 -1.462167

C -5.413079 -3.648435 -0.984920

C -5.986376 -3.324636 0.245907

C -5.471780 -2.229042 0.927214

N -4.460594 -1.492048 0.458532

H -5.787331 -4.500555 -1.560057

H -2.658856 -0.914589 -1.119170

H -3.910748 -3.124428 -2.427446

H -6.810815 -3.903778 0.665709

H -5.888935 -1.930877 1.896048

C -0.104000 0.218279 -3.316892

H -0.285408 1.302102 -3.258903

H 0.805604 0.001769 -2.740342

H 0.092540 -0.035627 -4.370594

C -2.495866 -0.361895 -3.733200

H -3.381306 -0.914012 -3.384226

H -2.756349 0.709669 -3.779857

H -2.265366 -0.691118 -4.758328

C -0.921277 -2.066851 -2.781986

H -0.617903 -2.393078 -3.788293

H -0.082118 -2.250166 -2.095764

H -1.768020 -2.689159 -2.462390

O -4.024147 1.593537 -0.122252

C -4.256719 2.841219 0.518262

H -3.312161 3.154503 0.981878

H -5.002772 2.708979 1.320830

C -4.773585 3.792429 -0.581646

H -4.124975 4.670045 -0.709982

H -5.776628 4.164920 -0.328899

C -4.813086 2.912313 -1.843106

H -5.664740 3.135555 -2.499695

H -3.890296 3.031180 -2.431074

C -4.866452 1.507405 -1.259948

H -5.891603 1.228669 -0.951908

H -4.474996 0.715097 -1.910270

**^5^TS_2-5_^’^**

Imaginary frequency: -1056.35 cm^-1^

Atom X Y Z

Fe 0.194287 -0.122047 0.084259

N -2.511849 -0.950366 -0.963033

N -1.265985 -2.710248 -1.188547

N -2.287376 1.564193 -0.244255

N -0.768257 2.941892 0.458291

N 2.264025 -0.802588 2.347079

N 1.689694 0.180439 -1.414499

H 2.124657 -0.741309 -1.496551

C -1.016544 1.623361 0.226047

C -2.818214 2.833382 -0.320455

H -3.831793 3.020601 -0.659251

C -1.870900 3.701166 0.110139

H -1.899871 4.782008 0.199747

C 0.418476 3.551681 1.107666

C 1.117438 4.482569 0.111583

H 1.968398 4.981166 0.598178

H 0.436218 5.259382 -0.266742

H 1.504018 3.910112 -0.741044

C -0.061632 4.334842 2.338499

H 0.806574 4.725042 2.889258

H -0.632661 3.676893 3.010264

H -0.696793 5.190783 2.068582

C 1.383978 2.469308 1.566792

H 2.279288 2.939862 1.996897

H 1.715601 1.865735 0.713263

H 0.930255 1.812663 2.319818

C -1.297649 -1.459041 -0.648088

C -3.227861 -1.867277 -1.700562

H -4.233189 -1.669377 -2.057311

C -2.458020 -2.973113 -1.843121

H -2.671719 -3.903025 -2.355465

C -0.251497 -3.748158 -0.884273

C -0.499083 -4.241872 0.546783

H 0.212034 -5.040536 0.808229

H -1.520598 -4.638901 0.646259

H -0.378077 -3.415912 1.261310

C 1.157798 -3.173479 -1.000355

H 1.887277 -3.978383 -0.830993

H 1.338140 -2.396497 -0.245932

H 1.334386 -2.754115 -2.001102

C -0.385394 -4.910739 -1.873552

H 0.427879 -5.627806 -1.693794

H -0.309575 -4.562660 -2.914819

H -1.330862 -5.457483 -1.750111

C -4.594567 0.614298 -0.983442

C -5.841052 0.552404 -0.348542

H -5.889010 0.257900 0.703481

C -7.025224 0.858407 -1.025011

H -7.982306 0.800688 -0.500140

C -6.988147 1.236838 -2.364756

H -7.912053 1.476830 -2.896791

C -5.758213 1.307625 -3.022622

H -5.716362 1.604245 -4.073944

C -4.585659 1.000402 -2.336454

H -3.628989 1.061780 -2.866228

C -3.390639 -0.177731 1.341686

C -3.261716 0.730629 2.403463

H -3.041223 1.781128 2.191278

C -3.387588 0.334369 3.735570

H -3.267167 1.069581 4.535457

C -3.658305 -0.996838 4.047940

H -3.753754 -1.312739 5.089826

C -3.809178 -1.919541 3.012735

H -4.024177 -2.966977 3.240408

C -3.677625 -1.509098 1.686103

H -3.787294 -2.257321 0.894762

C 0.978381 -0.831114 1.902215

C -0.019906 -1.248185 2.815943

H -1.064422 -1.274174 2.491864

C 0.294318 -1.610830 4.117990

H -0.495811 -1.916796 4.809366

C 1.630181 -1.574771 4.528031

H 1.929997 -1.852020 5.540289

C 2.573798 -1.161923 3.600082

H 3.634416 -1.108954 3.872870

C 1.300940 0.583448 -2.789916

B -3.198250 0.269764 -0.220991

Li 3.326207 -0.069026 0.859093

C 3.862963 1.775101 -0.732042

C 4.362027 2.872312 -1.462167

C 5.413079 3.648435 -0.984920

C 5.986376 3.324636 0.245907

C 5.471780 2.229042 0.927214

N 4.460594 1.492048 0.458532

H 5.787331 4.500555 -1.560057

H 2.658856 0.914589 -1.119170

H 3.910748 3.124428 -2.427446

H 6.810815 3.903778 0.665709

H 5.888935 1.930877 1.896048

C 0.104000 -0.218279 -3.316892

H 0.285408 -1.302102 -3.258903

H -0.805604 -0.001769 -2.740342

H -0.092540 0.035627 -4.370594

C 2.495866 0.361895 -3.733200

H 3.381306 0.914012 -3.384226

H 2.756349 -0.709669 -3.779857

H 2.265366 0.691118 -4.758328

C 0.921277 2.066851 -2.781986

H 0.617903 2.393078 -3.788293

H 0.082118 2.250166 -2.095764

H 1.768020 2.689159 -2.462390

O 4.024147 -1.593537 -0.122252

C 4.256719 -2.841219 0.518262

H 3.312161 -3.154503 0.981878

H 5.002772 -2.708979 1.320830

C 4.773585 -3.792429 -0.581646

H 4.124975 -4.670045 -0.709982

H 5.776628 -4.164920 -0.328899

C 4.813086 -2.912313 -1.843106

H 5.664740 -3.135555 -2.499695

H 3.890296 -3.031180 -2.431074

C 4.866452 -1.507405 -1.259948

H 5.891603 -1.228669 -0.951908

H 4.474996 -0.715097 -1.910270

**^5^TS_2-5_^’-2^**

Imaginary frequency: -1002.11 cm^-1^

Atom X Y Z

Fe -0.623241 0.311098 -0.129948

N 2.185468 0.322591 -1.183489

N 1.225609 1.973761 -2.212276

N 1.567438 -1.621501 0.491975

N -0.163972 -2.399610 1.540417

N -2.379774 2.637040 0.980411

N -1.966568 -0.409468 -1.615600

H -2.055259 0.382713 -2.255181

C 0.283738 -1.335227 0.818805

C 1.913944 -2.853504 0.999578

H 2.902079 -3.281301 0.865993

C 0.836493 -3.345488 1.660307

H 0.715043 -4.277686 2.201178

C -1.441249 -2.506462 2.287067

C -2.041283 -3.900938 2.067540

H -3.008476 -3.968625 2.585632

H -1.400535 -4.701317 2.463907

H -2.211188 -4.087075 0.997607

C -1.127495 -2.273093 3.771254

H -2.049726 -2.322524 4.368886

H -0.673477 -1.280126 3.907697

H -0.427196 -3.028576 4.157791

C -2.441798 -1.460046 1.808039

H -3.393664 -1.600626 2.339547

H -2.645965 -1.562478 0.734433

H -2.085972 -0.440554 2.015134

C 1.039320 1.039843 -1.235899

C 3.073484 0.791154 -2.124956

H 4.061635 0.363239 -2.258001

C 2.483305 1.827596 -2.770507

H 2.863258 2.449894 -3.571412

C 0.358669 3.159510 -2.435428

C 0.575597 4.126764 -1.264329

H -0.031062 5.035025 -1.398129

H 1.634187 4.420631 -1.200282

H 0.293031 3.652302 -0.314563

C -1.109948 2.746069 -2.512601

H -1.726859 3.627185 -2.739860

H -1.458969 2.346154 -1.552195

H -1.264712 2.001215 -3.307640

C 0.740897 3.839209 -3.754396

H 0.039166 4.662381 -3.947716

H 0.684526 3.137639 -4.600359

H 1.749454 4.274819 -3.720848

C 3.999381 -1.368685 -0.395777

C 5.229931 -1.213237 0.254009

H 5.304758 -0.518933 1.095525

C 6.365414 -1.923319 -0.146043

H 7.311517 -1.779394 0.382086

C 6.293611 -2.812741 -1.215281

H 7.179336 -3.369652 -1.530769

C 5.078532 -2.986616 -1.881577

H 5.010346 -3.681693 -2.722467

C 3.954554 -2.272825 -1.472796

H 3.009433 -2.419848 -2.006216

C 2.890061 0.457212 1.308547

C 2.721321 0.035540 2.637459

H 2.431246 -0.999613 2.842076

C 2.895338 0.901988 3.717446

H 2.745915 0.537624 4.737165

C 3.252959 2.231826 3.497278

H 3.387063 2.915705 4.339118

C 3.440499 2.675557 2.188742

H 3.721052 3.714859 1.998609

C 3.262915 1.797643 1.119192

H 3.407099 2.178740 0.103806

C -1.248307 1.894435 1.132421

C -0.277800 2.347458 2.052903

H 0.633151 1.763239 2.204195

C -0.449646 3.538002 2.747859

H 0.318255 3.882803 3.445585

C -1.608965 4.287054 2.533410

H -1.784943 5.232272 3.050062

C -2.551735 3.783836 1.647772

H -3.490198 4.318759 1.466324

C -1.630391 -1.572458 -2.478201

B 2.658087 -0.559311 0.046536

Li -3.350867 1.139800 0.205887

C -4.595446 -0.684160 -0.759013

C -5.487106 -1.697085 -1.165918

C -6.771408 -1.786863 -0.641437

C -7.183178 -0.847058 0.306600

C -6.276391 0.135375 0.677670

N -5.041622 0.208712 0.167982

H -7.453573 -2.579738 -0.962006

H -3.139135 -0.561178 -1.247842

H -5.159502 -2.433089 -1.907273

H -8.181430 -0.876418 0.746834

H -6.548597 0.896744 1.416983

C -0.180932 -1.500282 -2.973540

H 0.016901 -0.543950 -3.483614

H 0.528782 -1.585484 -2.139082

H 0.026890 -2.315546 -3.684629

C -2.572357 -1.579163 -3.692976

H -3.625738 -1.594522 -3.377849

H -2.418265 -0.676278 -4.307449

H -2.388390 -2.453933 -4.335764

C -1.822454 -2.859907 -1.671281

H -1.614633 -3.741229 -2.296734

H -1.135758 -2.884376 -0.814106

H -2.852975 -2.941725 -1.293472

**^5^TS_2-5_^’-3^**

Imaginary frequency: -330.13 cm^-1^

Atom X Y Z

Fe -0.231753 -0.034812 -0.212309

N -3.048941 -1.030123 -0.703109

N -1.784794 -2.684781 -1.301279

N -2.796056 1.467332 0.083816

N -1.258538 2.901671 0.607475

N 2.085443 -1.563989 1.083346

N 0.689468 0.449573 -2.052090

H 1.165450 -0.410156 -2.333146

C -1.471155 1.581713 0.355336

C -3.392816 2.707501 0.134276

H -4.453645 2.851945 -0.042073

C -2.434782 3.611798 0.456881

H -2.503846 4.685671 0.595957

C -0.013726 3.539865 1.098273

C 0.498168 4.520427 0.039838

H 1.406300 5.025989 0.399725

H -0.253106 5.288892 -0.197053

H 0.757587 3.982496 -0.880452

C -0.340869 4.271945 2.408418

H 0.580855 4.692159 2.836663

H -0.778146 3.573511 3.137621

H -1.045900 5.102325 2.258870

C 1.045729 2.483382 1.384880

H 1.958130 2.982654 1.742797

H 1.321595 1.930324 0.478836

H 0.704903 1.768659 2.146632

C -1.760224 -1.443927 -0.733698

C -3.866540 -1.991403 -1.252628

H -4.942269 -1.870172 -1.325544

C -3.083887 -3.032508 -1.628357

H -3.358344 -3.968900 -2.098158

C -0.649527 -3.639597 -1.318416

C -0.432732 -4.145661 0.113445

H 0.404494 -4.859523 0.146941

H -1.337599 -4.651677 0.482750

H -0.208024 -3.308717 0.787646

C 0.615045 -2.959036 -1.838963

H 1.405368 -3.714360 -1.958311

H 0.989059 -2.202344 -1.137491

H 0.430095 -2.495998 -2.819070

C -0.977015 -4.817365 -2.243019

H -0.100415 -5.477556 -2.302479

H -1.216601 -4.476024 -3.261525

H -1.812651 -5.423660 -1.865936

C -5.157492 0.408154 -0.198086

C -6.229528 0.275057 0.692824

H -6.031677 -0.041219 1.720772

C -7.545780 0.535976 0.301793

H -8.360197 0.422362 1.022038

C -7.821542 0.939571 -1.002300

H -8.849507 1.144510 -1.311537

C -6.771321 1.079410 -1.912272

H -6.975254 1.394882 -2.938892

C -5.464236 0.815763 -1.509373

H -4.651905 0.929041 -2.235282

C -3.441360 -0.387093 1.777230

C -3.289045 0.526325 2.833550

H -3.295384 1.600034 2.620867

C -3.112827 0.110141 4.153411

H -2.989720 0.852345 4.946685

C -3.088998 -1.250349 4.461204

H -2.947800 -1.582230 5.492973

C -3.251736 -2.179842 3.435132

H -3.235262 -3.249875 3.658676

C -3.426577 -1.748162 2.119668

H -3.540145 -2.502512 1.335384

C 0.883503 -0.962087 1.293948

C 0.224434 -1.209300 2.523796

H -0.734099 -0.720039 2.720307

C 0.750083 -2.082427 3.465531

H 0.217229 -2.274187 4.401045

C 1.958436 -2.727720 3.183356

H 2.404650 -3.443775 3.876507

C 2.585352 -2.419237 1.985035

H 3.551965 -2.875480 1.737369

C -0.019631 0.952702 -3.253434

B -3.612661 0.118386 0.228306

Li 3.375855 -0.216418 0.089608

C 2.855853 2.132394 -1.593866

C 3.183171 3.375039 -2.178872

C 4.256955 4.141497 -1.739163

C 5.039938 3.655313 -0.690636

C 4.698737 2.419799 -0.155291

N 3.658632 1.693879 -0.582107

H 4.488871 5.106280 -2.201073

H 1.593036 1.189949 -1.868540

H 2.564688 3.752575 -3.001415

H 5.895126 4.212997 -0.303606

H 5.287668 1.979754 0.657709

C -1.235551 0.085021 -3.597020

H -0.954419 -0.973292 -3.713266

H -1.996907 0.142230 -2.807180

H -1.692472 0.419365 -4.541842

C 0.957056 0.952129 -4.441185

H 1.848973 1.553523 -4.209635

H 1.287920 -0.075659 -4.669369

H 0.485469 1.359074 -5.349275

C -0.492480 2.383330 -2.981037

H -1.025518 2.784716 -3.856248

H -1.176684 2.413764 -2.121043

H 0.360516 3.042026 -2.766551

O 4.125989 -1.277760 -1.381247

C 4.199574 -2.698658 -1.331988

H 3.321374 -3.054882 -0.773170

H 5.106667 -3.002503 -0.782980

C 4.217355 -3.179894 -2.789944

H 3.603667 -4.078948 -2.941620

H 5.243597 -3.422772 -3.105353

C 3.702906 -1.961281 -3.560616

H 4.045818 -1.927264 -4.603624

H 2.602339 -1.939170 -3.564646

C 4.241187 -0.813314 -2.722255

H 5.300830 -0.603641 -2.958147

H 3.672053 0.122883 -2.805533

O 4.586243 -0.070030 1.720915

C 5.790999 -0.744496 2.046697

H 6.569788 -0.451515 1.326132

H 5.634647 -1.835079 1.959223

C 6.107201 -0.345029 3.485496

H 6.685462 0.592305 3.504828

H 6.686222 -1.110982 4.019319

C 4.708102 -0.120542 4.061228

H 4.238150 -1.081150 4.323041

H 4.697441 0.520980 4.952677

C 3.981102 0.505554 2.877967

H 2.903208 0.288214 2.857115

H 4.116200 1.601374 2.843152

**^5^TS_2-5_^’-4^**

Imaginary frequency: -1136.51 cm^-1^

Atom X Y Z

Fe 0.184643 0.117140 0.362187

B -3.033881 -0.589938 -0.349267

C -0.662241 -0.530768 -1.453343

Li 3.504994 0.086750 0.360310

N -2.010608 -0.512452 -1.558921

N -0.192790 -0.786965 -2.705749

C -2.380536 -0.757402 -2.861937

H -3.418675 -0.799257 -3.174376

C -1.248397 -0.934830 -3.587249

H -1.121661 -1.159521 -4.640826

N -2.748707 0.516265 0.747178

C 1.209152 -1.043789 -3.100900

N -1.825827 1.453524 2.470635

C 1.374149 -2.557588 -3.294623

H 1.121585 -3.084157 -2.362075

H 2.413143 -2.798974 -3.565873

H 0.715895 -2.934202 -4.091593

N -0.185738 3.254446 0.385493

C 1.518677 -0.294859 -4.404059

H 0.896855 -0.637761 -5.242630

H 2.568672 -0.462243 -4.683711

H 1.367720 0.787756 -4.283198

N 2.686161 -1.346286 1.497221

C 2.174809 -0.569873 -2.020088

H 2.024832 0.485091 -1.756844

H 3.203378 -0.666803 -2.393240

H 2.078574 -1.179630 -1.111123

C -1.561942 0.704211 1.370384

C -3.747119 1.148104 1.453512

H -4.786716 1.120995 1.144212

C -3.175484 1.738022 2.533637

H -3.623973 2.322402 3.330267

C -0.885670 1.862725 3.542675

C -1.285018 1.111115 4.820007

H -2.301875 1.374303 5.147684

H -0.591043 1.358238 5.636987

H -1.248439 0.025025 4.646926

C -0.979362 3.380529 3.739534

H -0.660126 3.886269 2.818030

H -0.311458 3.687058 4.557914

H -1.996194 3.706930 4.001855

C 0.549905 1.508276 3.170579

H 0.695353 0.424401 3.079285

H 1.220491 1.873457 3.962315

H 0.826737 2.008919 2.232271

C -4.528687 -0.337141 -0.943761

C -5.546834 -1.296052 -0.883734

H -5.342975 -2.261548 -0.412265

C -6.817896 -1.050396 -1.411258

H -7.591617 -1.819958 -1.348047

C -7.099994 0.171889 -2.015994

H -8.092708 0.367590 -2.428963

C -6.102048 1.146552 -2.089531

H -6.312465 2.109966 -2.561486

C -4.839761 0.889257 -1.560365

H -4.066330 1.662619 -1.623760

C -2.847765 -2.048029 0.366851

C -2.334918 -3.160047 -0.318737

H -2.045152 -3.058376 -1.369041

C -2.165680 -4.398679 0.301526

H -1.753342 -5.238795 -0.263469

C -2.516206 -4.564890 1.640476

H -2.383687 -5.532211 2.131564

C -3.041118 -3.480811 2.344286

H -3.322357 -3.594812 3.394524

C -3.201129 -2.247695 1.711794

H -3.602359 -1.410526 2.291411

C 0.388959 2.427060 -0.516505

C -0.014815 2.485871 -1.860877

H 0.465580 1.840002 -2.597086

C -1.049822 3.326030 -2.259421

H -1.381811 3.350489 -3.300565

C -1.661008 4.124336 -1.297229

H -2.487204 4.792581 -1.547312

C -1.175650 4.055919 0.009143

H -1.620936 4.685371 0.789735

C 1.335313 -1.337115 1.349702

C 0.593058 -2.371910 1.961976

H -0.493951 -2.391969 1.845612

C 1.217612 -3.362935 2.709123

H 0.628452 -4.159190 3.172165

C 2.605465 -3.321775 2.857927

H 3.144052 -4.069727 3.442850

C 3.289603 -2.291677 2.227469

H 4.381489 -2.220638 2.305687

N 2.950610 1.948444 0.335494

H 2.783351 2.248403 1.300277

H 1.588305 2.025121 -0.139517

C 3.806935 2.959799 -0.284943

C 3.145226 4.351798 -0.299082

H 3.821069 5.120717 -0.708690

H 2.226783 4.346481 -0.903473

H 2.863887 4.651619 0.723055

C 4.082544 2.523628 -1.729733

H 4.750442 3.229818 -2.248097

H 4.549579 1.524935 -1.750028

H 3.141371 2.468484 -2.299744

C 5.143025 3.069874 0.471000

H 5.650736 2.093375 0.500057

H 5.827416 3.800615 0.007781

H 4.968099 3.384912 1.512632

O 5.062510 -0.845515 -0.477897

C 6.424667 -0.482505 -0.243791

H 6.531590 -0.194934 0.815626

H 6.676705 0.391829 -0.862436

C 7.253472 -1.721337 -0.583439

H 8.153195 -1.806430 0.040603

H 7.575643 -1.692963 -1.635875

C 6.249431 -2.854540 -0.369348

H 6.503454 -3.774972 -0.912089

H 6.160658 -3.102130 0.700192

C 4.963782 -2.213740 -0.866785

H 4.884346 -2.281240 -1.967162

H 4.049422 -2.631355 -0.423878

**^5^TS_2-5_^’-5^**

Imaginary frequency: -1070.42 cm^-1^

Atom X Y Z

Fe -0.250315 0.142298 0.080948

B -3.554792 -0.573546 0.149328

C -1.394929 -1.007141 -1.261069

Li 3.301375 0.035818 -0.252406

N -2.742751 -0.958948 -1.154497

N -1.135682 -1.688751 -2.410669

C -3.319716 -1.607228 -2.223139

H -4.394709 -1.700008 -2.336720

C -2.320135 -2.071679 -3.013225

H -2.366181 -2.641158 -3.935147

N -3.088366 0.812932 0.756885

C 0.186770 -2.126442 -2.910232

N -1.895338 2.204825 1.912924

C 0.339741 -3.619010 -2.586805

H 0.259930 -3.775705 -1.500580

H 1.321245 -3.985536 -2.924038

H -0.438901 -4.219832 -3.080309

N -0.637188 3.143065 -0.860095

C 0.261743 -1.889806 -4.424619

H -0.478419 -2.481116 -4.981730

H 1.256796 -2.179348 -4.791681

H 0.107265 -0.827644 -4.665091

N 2.353244 -1.114991 1.177757

C 1.309472 -1.347668 -2.235473

H 1.194475 -0.262265 -2.337631

H 2.264468 -1.612273 -2.708338

H 1.386209 -1.587736 -1.167337

C -1.815292 1.123457 1.095619

C -3.956572 1.697994 1.355195

H -5.032532 1.632861 1.231992

C -3.215726 2.573223 2.080279

H -3.527065 3.410579 2.695961

C -0.795257 2.882201 2.639054

C -0.979503 2.591296 4.134968

H -1.932553 2.990386 4.513071

H -0.165454 3.051819 4.714395

H -0.965920 1.505477 4.313249

C -0.855748 4.387747 2.354005

H -0.696945 4.560190 1.280664

H -0.060138 4.900927 2.914091

H -1.814132 4.833217 2.657507

C 0.561792 2.358567 2.181505

H 0.685618 1.290679 2.406332

H 1.348388 2.914973 2.713107

H 0.686418 2.534959 1.103721

C -5.124983 -0.439985 -0.262938

C -6.127080 -1.261729 0.266402

H -5.856601 -2.028499 0.997834

C -7.464723 -1.127099 -0.116950

H -8.223684 -1.784695 0.314908

C -7.831737 -0.157725 -1.046950

H -8.876557 -0.049356 -1.348596

C -6.851571 0.675723 -1.591002

H -7.128206 1.440470 -2.321662

C -5.522420 0.530935 -1.201219

H -4.763464 1.191208 -1.635184

C -3.265897 -1.721093 1.278077

C -2.859723 -3.021158 0.940317

H -2.727440 -3.287546 -0.112660

C -2.599902 -3.990897 1.909795

H -2.274609 -4.989662 1.606986

C -2.748234 -3.686796 3.262011

H -2.544254 -4.442111 4.025188

C -3.163334 -2.406123 3.627941

H -3.286126 -2.151679 4.684075

C -3.415803 -1.446194 2.647707

H -3.727221 -0.445562 2.963408

C -0.225333 2.040220 -1.524608

C -0.857985 1.679747 -2.725747

H -0.508453 0.806707 -3.278484

C -1.953005 2.397192 -3.196657

H -2.463307 2.099787 -4.116416

C -2.391678 3.494206 -2.460942

H -3.254158 4.086120 -2.773170

C -1.683718 3.828622 -1.306259

H -1.989726 4.699006 -0.712381

C 1.005650 -1.007383 1.300690

C 0.370598 -1.726035 2.339708

H -0.715254 -1.658247 2.449190

C 1.096340 -2.519518 3.218713

H 0.587610 -3.075899 4.010667

C 2.482996 -2.593616 3.068404

H 3.103258 -3.198831 3.732528

C 3.055620 -1.869136 2.032087

H 4.140039 -1.892895 1.871884

N 2.443546 1.711454 -0.952222

H 2.414615 2.339799 -0.143161

H 1.001934 1.703698 -1.227623

C 3.177085 2.429670 -1.996747

C 2.509950 3.770978 -2.363750

H 3.107249 4.336061 -3.098853

H 1.507942 3.613489 -2.787629

H 2.391135 4.397411 -1.464898

C 3.216545 1.540779 -3.246230

H 3.795752 2.006530 -4.059627

H 3.666502 0.563658 -3.010046

H 2.196122 1.358747 -3.618861

C 4.620199 2.729185 -1.548124

H 5.134759 1.803970 -1.250671

H 5.208494 3.225700 -2.338395

H 4.613728 3.392120 -0.666741

O 4.415691 -1.339410 -1.254004

C 5.688919 -1.109653 -1.829790

H 6.012278 -0.102198 -1.538132

H 5.605347 -1.135624 -2.931273

C 6.598847 -2.242134 -1.311523

H 7.341456 -1.869309 -0.592806

H 7.152769 -2.708132 -2.138347

C 5.615459 -3.229309 -0.649869

H 5.808136 -4.276756 -0.918536

H 5.661304 -3.154163 0.446978

C 4.256125 -2.740343 -1.141719

H 4.011956 -3.171595 -2.132144

H 3.420745 -2.935493 -0.458393

O 4.821171 0.655038 1.002259

C 6.103226 0.198685 1.394028

H 6.856550 0.532957 0.660769

H 6.100458 -0.905707 1.394715

C 6.355151 0.771299 2.793013

H 6.924521 1.711074 2.724961

H 6.921881 0.082001 3.433806

C 4.936801 1.048392 3.295940

H 4.473472 0.130312 3.690073

H 4.894350 1.818275 4.078305

C 4.237716 1.468749 2.012169

H 3.155733 1.281015 2.011528

H 4.411557 2.535008 1.778477

**^5^TS_2-5_^’-6^**

Imaginary frequency: -1179.15 cm^-1^

Atom X Y Z

Fe -0.791775 0.419042 0.153379

B 2.493690 -0.166446 -0.203373

C 0.291913 0.051906 -1.607979

Li -3.626008 1.242209 -0.119898

N 1.553817 -0.411915 -1.460768

N -0.009070 -0.086167 -2.928213

C 2.037097 -0.837140 -2.676959

H 3.038076 -1.237813 -2.797605

C 1.066051 -0.634740 -3.602413

H 1.067592 -0.825264 -4.670466

N 1.805248 -0.600892 1.156545

C -1.221248 0.386556 -3.635087

N 0.548875 -0.460566 2.918933

C -0.824449 1.601839 -4.484822

H -0.401506 2.388329 -3.842371

H -1.703683 2.008707 -5.005846

H -0.072837 1.334337 -5.241970

N -1.400671 -2.429886 1.385095

C -1.767509 -0.739278 -4.522970

H -1.041233 -1.060646 -5.282435

H -2.665363 -0.388979 -5.051957

H -2.049397 -1.616276 -3.922558

N -2.729399 2.946210 0.137602

C -2.300600 0.804586 -2.640598

H -2.559272 -0.018493 -1.957078

H -3.210067 1.070824 -3.199345

H -1.985944 1.681069 -2.060169

C 0.602477 -0.163763 1.596579

C 2.498553 -1.169125 2.201000

H 3.496719 -1.577834 2.084066

C 1.717256 -1.084066 3.307642

H 1.908281 -1.409662 4.324794

C -0.527063 -0.126071 3.883426

C 0.024238 0.937914 4.841968

H 0.887741 0.563038 5.411607

H -0.751629 1.239400 5.561128

H 0.343115 1.827424 4.278067

C -0.936100 -1.396496 4.638437

H -1.340622 -2.128041 3.925402

H -1.717066 -1.151731 5.373152

H -0.096135 -1.846603 5.186943

C -1.752261 0.422027 3.158592

H -1.537956 1.371102 2.650172

H -2.546319 0.606500 3.896944

H -2.121460 -0.321765 2.437876

C 3.831560 -1.071361 -0.400482

C 5.113392 -0.522490 -0.522735

H 5.235156 0.563447 -0.478357

C 6.241860 -1.328347 -0.700318

H 7.229600 -0.869414 -0.792480

C 6.109949 -2.713393 -0.759656

H 6.990022 -3.346303 -0.897951

C 4.841952 -3.287145 -0.640000

H 4.727086 -4.373403 -0.684261

C 3.725594 -2.473492 -0.463694

H 2.736654 -2.935806 -0.369790

C 2.783946 1.439322 -0.110026

C 2.715295 2.279808 -1.231937

H 2.466392 1.855187 -2.209254

C 2.938100 3.654403 -1.141538

H 2.864797 4.277925 -2.036359

C 3.248217 4.233887 0.087899

H 3.421844 5.310209 0.164458

C 3.339090 3.421176 1.218076

H 3.584306 3.859601 2.188971

C 3.110610 2.048969 1.112697

H 3.174441 1.437241 2.017921

C -1.646154 -1.908270 0.160707

C -1.178793 -2.581104 -0.980234

H -1.402431 -2.181433 -1.970280

C -0.394549 -3.725183 -0.864692

H -0.005410 -4.229288 -1.752968

C -0.106670 -4.201991 0.410191

H 0.515599 -5.085502 0.564724

C -0.652999 -3.521404 1.499548

H -0.465916 -3.883534 2.518133

C -1.486625 2.415445 0.300794

C -0.417533 3.298636 0.563352

H 0.593662 2.902185 0.692424

C -0.625296 4.670231 0.653386

H 0.215853 5.340863 0.847695

C -1.917032 5.171252 0.488123

H -2.131453 6.239439 0.552215

C -2.936430 4.264039 0.233055

H -3.967043 4.608464 0.095558

N -4.019515 -0.564649 0.337783

H -4.081307 -0.600201 1.360002

H -2.723400 -1.127086 0.163604

C -5.073923 -1.437818 -0.180552

C -4.991212 -2.841099 0.447920

H -5.792440 -3.502153 0.079463

H -4.023125 -3.313117 0.224676

H -5.083290 -2.776055 1.544261

C -4.898632 -1.561277 -1.698868

H -5.668042 -2.213660 -2.140149

H -4.976226 -0.574339 -2.183611

H -3.912672 -1.987430 -1.941827

C -6.460950 -0.841915 0.115028

H -6.558290 0.154750 -0.346273

H -7.276744 -1.477896 -0.267536

H -6.604634 -0.724919 1.201342

**^5^TS^α^_5-2_**

Imaginary frequency: -1109.43 cm^-1^

Atom X Y Z

Fe -0.194287 0.122047 0.084259

N 2.511849 0.950366 -0.963033

N 1.265985 2.710248 -1.188547

N 2.287376 -1.564193 -0.244255

N 0.768257 -2.941892 0.458291

N -2.264025 0.802588 2.347079

N -1.689694 -0.180439 -1.414499

H -2.124657 0.741309 -1.496551

C 1.016544 -1.623361 0.226047

C 2.818214 -2.833382 -0.320455

H 3.831793 -3.020601 -0.659251

C 1.870900 -3.701166 0.110139

H 1.899871 -4.782008 0.199747

C -0.418476 -3.551681 1.107666

C -1.117438 -4.482569 0.111583

H -1.968398 -4.981166 0.598178

H -0.436218 -5.259382 -0.266742

H -1.504018 -3.910112 -0.741044

C 0.061632 -4.334842 2.338499

H -0.806574 -4.725042 2.889258

H 0.632661 -3.676893 3.010264

H 0.696793 -5.190783 2.068582

C -1.383978 -2.469308 1.566792

H -2.279288 -2.939862 1.996897

H -1.715601 -1.865735 0.713263

H -0.930255 -1.812663 2.319818

C 1.297649 1.459041 -0.648088

C 3.227861 1.867277 -1.700562

H 4.233189 1.669377 -2.057311

C 2.458020 2.973113 -1.843121

H 2.671719 3.903025 -2.355465

C 0.251497 3.748158 -0.884273

C 0.499083 4.241872 0.546783

H -0.212034 5.040536 0.808229

H 1.520598 4.638901 0.646259

H 0.378077 3.415912 1.261310

C -1.157798 3.173479 -1.000355

H -1.887277 3.978383 -0.830993

H -1.338140 2.396497 -0.245932

H -1.334386 2.754115 -2.001102

C 0.385394 4.910739 -1.873552

H -0.427879 5.627806 -1.693794

H 0.309575 4.562660 -2.914819

H 1.330862 5.457483 -1.750111

C 4.594567 -0.614298 -0.983442

C 5.841052 -0.552404 -0.348542

H 5.889010 -0.257900 0.703481

C 7.025224 -0.858407 -1.025011

H 7.982306 -0.800688 -0.500140

C 6.988147 -1.236838 -2.364756

H 7.912053 -1.476830 -2.896791

C 5.758213 -1.307625 -3.022622

H 5.716362 -1.604245 -4.073944

C 4.585659 -1.000402 -2.336454

H 3.628989 -1.061780 -2.866228

C 3.390639 0.177731 1.341686

C 3.261716 -0.730629 2.403463

H 3.041223 -1.781128 2.191278

C 3.387588 -0.334369 3.735570

H 3.267167 -1.069581 4.535457

C 3.658305 0.996838 4.047940

H 3.753754 1.312739 5.089826

C 3.809178 1.919541 3.012735

H 4.024177 2.966977 3.240408

C 3.677625 1.509098 1.686103

H 3.787294 2.257321 0.894762

C -0.978381 0.831114 1.902215

C 0.019906 1.248185 2.815943

H 1.064422 1.274174 2.491864

C -0.294318 1.610830 4.117990

H 0.495811 1.916796 4.809366

C -1.630181 1.574771 4.528031

H -1.929997 1.852020 5.540289

C -2.573798 1.161923 3.600082

H -3.634416 1.108954 3.872870

C -1.300940 -0.583448 -2.789916

B 3.198250 -0.269764 -0.220991

Li -3.326207 0.069026 0.859093

C -3.862963 -1.775101 -0.732042

C -4.362027 -2.872312 -1.462167

C -5.413079 -3.648435 -0.984920

C -5.986376 -3.324636 0.245907

C -5.471780 -2.229042 0.927214

N -4.460594 -1.492048 0.458532

H -5.787331 -4.500555 -1.560057

H -2.658856 -0.914589 -1.119170

H -3.910748 -3.124428 -2.427446

H -6.810815 -3.903778 0.665709

H -5.888935 -1.930877 1.896048

C -0.104000 0.218279 -3.316892

H -0.285408 1.302102 -3.258903

H 0.805604 0.001769 -2.740342

H 0.092540 -0.035627 -4.370594

C -2.495866 -0.361895 -3.733200

H -3.381306 -0.914012 -3.384226

H -2.756349 0.709669 -3.779857

H -2.265366 -0.691118 -4.758328

C -0.921277 -2.066851 -2.781986

H -0.617903 -2.393078 -3.788293

H -0.082118 -2.250166 -2.095764

H -1.768020 -2.689159 -2.462390

O -4.024147 1.593537 -0.122252

C -4.256719 2.841219 0.518262

H -3.312161 3.154503 0.981878

H -5.002772 2.708979 1.320830

C -4.773585 3.792429 -0.581646

H -4.124975 4.670045 -0.709982

H -5.776628 4.164920 -0.328899

C -4.813086 2.912313 -1.843106

H -5.664740 3.135555 -2.499695

H -3.890296 3.031180 -2.431074

C -4.866452 1.507405 -1.259948

H -5.891603 1.228669 -0.951908

H -4.474996 0.715097 -1.910270

**^5^TS^β^_5-2_**

Imaginary frequency: -1258.51 cm^-1^

Atom X Y Z

Fe -0.005454 -0.095184 -0.099576

N -2.745255 -0.398062 -1.310769

N -1.479410 -1.701362 -2.493954

N -2.534422 1.357679 0.644697

N -0.993392 2.275313 1.860472

N 2.136800 -1.694856 1.405090

N 1.261044 1.014841 -1.359629

H 1.531218 0.325334 -2.063604

C -1.224255 1.261912 0.977386

C -3.111959 2.417536 1.307863

H -4.160789 2.672179 1.196189

C -2.154196 2.996926 2.072404

H -2.214818 3.848128 2.742259

C 0.227542 2.515360 2.664368

C 0.606438 3.999858 2.583492

H 1.516207 4.180724 3.174580

H -0.180416 4.654069 2.984708

H 0.804358 4.294927 1.543538

C -0.081929 2.110033 4.112568

H 0.808841 2.241375 4.744834

H -0.385737 1.053228 4.149253

H -0.894492 2.717726 4.537281

C 1.393157 1.675726 2.150466

H 2.294445 1.936569 2.725811

H 1.587384 1.875653 1.088376

H 1.202098 0.603138 2.282414

C -1.498208 -0.918785 -1.376464

C -3.494006 -0.835956 -2.379675

H -4.528077 -0.542047 -2.527009

C -2.711638 -1.656803 -3.123571

H -2.942425 -2.192156 -4.036551

C -0.434507 -2.707025 -2.810605

C -0.615320 -3.885301 -1.844665

H 0.113305 -4.680590 -2.064098

H -1.628200 -4.305643 -1.936240

H -0.472240 -3.552893 -0.807002

C 0.963044 -2.109711 -2.650596

H 1.713160 -2.865089 -2.927078

H 1.154237 -1.816722 -1.608881

H 1.092010 -1.238512 -3.309930

C -0.595342 -3.182541 -4.258580

H 0.238204 -3.852982 -4.510526

H -0.578185 -2.336780 -4.962401

H -1.524032 -3.750623 -4.409789

C -4.855017 0.822423 -0.397195

C -6.057702 0.365022 0.155069

H -6.033621 -0.448607 0.885329

C -7.288490 0.922420 -0.203037

H -8.209528 0.542498 0.246663

C -7.343478 1.959842 -1.130516

H -8.303997 2.397880 -1.413049

C -6.158780 2.435947 -1.696613

H -6.189292 3.250072 -2.425483

C -4.939007 1.871902 -1.330630

H -4.019029 2.256494 -1.783556

C -3.489600 -1.020281 1.089174

C -3.323827 -0.811135 2.466951

H -3.132412 0.199503 2.839965

C -3.379969 -1.858855 3.387364

H -3.235136 -1.656121 4.451745

C -3.613717 -3.162188 2.951914

H -3.656195 -3.986478 3.668263

C -3.795573 -3.399332 1.589283

H -3.981205 -4.415340 1.230842

C -3.733986 -2.342317 0.681077

H -3.868955 -2.559894 -0.383010

C 0.809483 -1.614252 1.111853

C -0.053126 -2.559410 1.710329

H -1.124074 -2.507873 1.497341

C 0.429236 -3.540269 2.567718

H -0.256159 -4.256815 3.028835

C 1.799643 -3.590641 2.834577

H 2.228607 -4.340607 3.501805

C 2.609534 -2.642512 2.226670

H 3.691574 -2.626728 2.402305

C 0.792806 2.194690 -2.126251

B -3.403494 0.199871 0.000891

Li 3.242117 -0.274089 0.588420

C 3.803159 1.600608 -0.570678

C 4.255824 2.670766 0.214798

C 5.547259 3.178044 0.056843

C 6.379215 2.582757 -0.890409

H 5.909189 4.017699 0.654932

H 2.482110 1.286863 -0.843776

H 3.594289 3.130393 0.958462

H 7.403831 2.944338 -1.034450

C -0.591909 1.950876 -2.744048

H -0.590413 1.044524 -3.370592

H -1.353531 1.812977 -1.963890

H -0.896368 2.801508 -3.374913

C 1.788885 2.505587 -3.255890

H 2.791453 2.718551 -2.857486

H 1.871409 1.648903 -3.946305

H 1.462443 3.376254 -3.845901

C 0.709755 3.397981 -1.182364

H 0.382836 4.297838 -1.725525

H -0.013763 3.205967 -0.378110

H 1.691359 3.611971 -0.731740

C 4.743683 1.121000 -1.496986

H 4.457924 0.302111 -2.175153

N 5.992654 1.563931 -1.659275

O 5.003125 -1.144694 0.694563

C 6.243377 -0.540435 1.072976

H 6.132426 0.551933 1.015813

C 7.275382 -1.097248 0.101222

H 8.288381 -1.111121 0.525678

H 7.286622 -0.486831 -0.814822

C 6.715943 -2.491411 -0.184987

H 7.082179 -2.924667 -1.125412

H 6.965994 -3.187635 0.631284

C 5.215912 -2.225701 -0.215248

H 4.885608 -1.916216 -1.222545

H 4.601124 -3.079535 0.105391

H 6.467647 -0.820582 2.117104

**^5^TS^γ^_5-2_**

Imaginary frequency: -1238.38 cm^-1^

Atom X Y Z

Fe -0.061602 0.173682 0.348755

N -2.961686 0.061841 1.140527

N -2.019531 1.227306 2.707307

N -2.301744 -1.312201 -1.010022

N -0.519860 -1.924815 -2.078728

N 2.099374 2.121136 -0.686001

N 1.113327 -1.021459 1.606100

H 1.229216 -0.421748 2.424596

C -0.969311 -1.084161 -1.101658

C -2.678185 -2.280327 -1.914158

H -3.706082 -2.611484 -2.018533

C -1.568701 -2.669261 -2.588164

H -1.452809 -3.401720 -3.380028

C 0.825991 -1.968023 -2.693722

C 1.315462 -3.421089 -2.748777

H 2.317339 -3.457221 -3.201377

H 0.657839 -4.058533 -3.356196

H 1.375106 -3.849825 -1.738760

C 0.716659 -1.375155 -4.105743

H 1.704947 -1.354047 -4.588943

H 0.329476 -0.346735 -4.052465

H 0.040030 -1.965752 -4.740418

C 1.819736 -1.141128 -1.884149

H 2.820456 -1.263963 -2.326023

H 1.852707 -1.482001 -0.840520

H 1.555228 -0.075110 -1.911154

C -1.797079 0.658110 1.487498

C -3.896649 0.242784 2.134503

H -4.905140 -0.151558 2.065638

C -3.317382 0.975586 3.117432

H -3.730084 1.321128 4.057475

C -1.144127 2.237996 3.352769

C -1.332323 3.562840 2.602289

H -0.738371 4.361077 3.072762

H -2.390769 3.863981 2.611642

H -1.012178 3.456917 1.556508

C 0.320481 1.808268 3.294960

H 0.939247 2.565736 3.797578

H 0.674266 1.725949 2.257844

H 0.464504 0.848380 3.812680

C -1.542604 2.403825 4.823507

H -0.823657 3.073387 5.316018

H -1.529238 1.439267 5.353051

H -2.537071 2.857599 4.938476

C -4.791447 -1.113142 -0.291388

C -5.931845 -0.645869 -0.956076

H -5.870464 0.281178 -1.532807

C -7.146841 -1.334776 -0.901890

H -8.018646 -0.943120 -1.432385

C -7.248669 -2.517923 -0.174438

H -8.197082 -3.059003 -0.129652

C -6.126408 -3.007360 0.497623

H -6.193836 -3.935238 1.071598

C -4.921637 -2.311353 0.434784

H -4.050820 -2.708602 0.967110

C -3.390323 1.043709 -1.213673

C -2.968062 1.102916 -2.550257

H -2.605255 0.195394 -3.042146

C -2.979835 2.293658 -3.278467

H -2.631691 2.301047 -4.314669

C -3.428339 3.472796 -2.686254

H -3.437676 4.407945 -3.251791

C -3.868065 3.442340 -1.362516

H -4.224930 4.357857 -0.883269

C -3.846523 2.245668 -0.646365

H -4.185557 2.253438 0.394313

C 0.765314 1.925017 -0.487467

C -0.120992 2.920045 -0.956211

H -1.196880 2.772345 -0.829781

C 0.341742 4.066416 -1.589600

H -0.362669 4.819742 -1.952950

C 1.717129 4.229573 -1.766911

H 2.133766 5.107144 -2.264706

C 2.549911 3.221957 -1.302751

H 3.633898 3.286563 -1.446571

C 0.674787 -2.334045 2.131885

B -3.358505 -0.339761 -0.339737

Li 3.185250 0.520767 -0.263840

C 4.591017 -0.597694 2.067175

C 3.778198 -1.210116 1.100282

C 4.416176 -2.213800 0.354428

C 5.759725 -2.521397 0.569855

H 3.869799 -2.774966 -0.413206

H 2.403394 -1.088731 1.233683

H 6.252551 -3.303951 -0.020773

C -0.790231 -2.293943 2.591064

H -0.949674 -1.493833 3.331489

H -1.462697 -2.098607 1.743620

H -1.085486 -3.250217 3.052208

C 1.560879 -2.730398 3.324538

H 2.620039 -2.783050 3.031954

H 1.469182 -1.989882 4.137196

H 1.266988 -3.709951 3.733198

C 0.817327 -3.384199 1.026355

H 0.505062 -4.376333 1.386507

H 0.187511 -3.122049 0.164739

H 1.863127 -3.460598 0.690866

C 5.932043 -0.959720 2.211640

H 6.565015 -0.465033 2.959129

N 6.521359 -1.897626 1.470369

H 4.181962 0.176559 2.728263

O 4.948567 0.907690 -1.033574

C 5.661945 0.080137 -1.952274

H 5.254532 -0.939402 -1.891836

C 7.120100 0.166765 -1.523270

H 7.815230 -0.026951 -2.351250

H 7.323038 -0.557283 -0.719443

C 7.198745 1.593908 -0.981582

H 8.035911 1.750603 -0.288402

H 7.297000 2.317708 -1.806048

C 5.846248 1.743427 -0.295967

H 5.880238 1.389043 0.746806

H 5.457986 2.772642 -0.304531

H 5.509178 0.466665 -2.975180

**Pyridine**

Atom X Y Z

C -1.139841 -0.723003 -0.000221

C -1.197103 0.670781 -0.000117

C 0.000062 1.382625 0.000096

C 1.197163 0.670683 0.000218

C 1.139776 -0.723094 0.000130

N -0.000062 -1.411984 -0.000104

H 0.000083 2.475004 0.000143

H -2.063754 -1.311820 -0.000380

H -2.161156 1.182486 -0.000219

H 2.161284 1.182264 0.000366

H 2.063631 -1.311998 0.000182

**THF**

Atom X Y Z

O 0.000118 -1.235550 -0.000104

C 1.155621 -0.432728 -0.132483

H 1.529726 -0.479209 -1.173426

H 1.948954 -0.823779 0.524454

C 0.729049 0.989816 0.228474

H 1.345425 1.759348 -0.257062

H 0.787789 1.145181 1.317729

C -0.729290 0.989634 -0.228533

H -1.345833 1.759090 0.256911

H -0.788079 1.144815 -1.317811

C -1.155496 -0.432957 0.132615

H -1.529304 -0.479425 1.173671

H -1.948933 -0.824213 -0.524069

***^t^*BuNH_2_**

Atom X Y Z

N -0.000456 0.182026 1.473258

H 0.813585 0.727531 1.754048

H -0.814964 0.727083 1.753565

C 0.000006 -0.004965 0.020375

C 0.000378 1.331864 -0.741256

H 0.000604 1.182697 -1.832781

H -0.890578 1.926939 -0.482523

H 0.891417 1.926643 -0.482135

C -1.253284 -0.806331 -0.342913

H -1.292895 -1.015007 -1.422808

H -1.267116 -1.760555 0.203471

H -2.165892 -0.248453 -0.075863

C 1.253368 -0.806583 -0.342133

H 1.266647 -1.760810 0.204253

H 1.293657 -1.015250 -1.422009

H 2.165918 -0.248909 -0.074461

**The Cartesian Coordinates of the stationary points discussed in Figure 3-5 and Figure S27-S30.**

**1^-^** (singlet)

Atom X Y Z

Fe -0.212902 0.170239 -0.318352

N 0.257704 -0.626331 2.632036

N -1.872794 -0.112240 -0.599039

N 1.986208 -0.456787 1.334333

N 1.083582 2.978507 -0.762516

N 2.404381 1.310673 -0.372871

C -3.151293 -0.510625 -0.421057

C -4.202785 0.388005 -0.042594

C -3.926823 1.879301 -0.038960

H -2.834148 1.975637 -0.020348

C 0.634409 -0.295416 1.358084

C -1.143741 -0.726095 3.102431

C 2.093759 -1.134257 -1.151123

C 1.092883 1.643862 -0.473618

C -3.500876 -1.898105 -0.582830

C -5.482345 -0.097025 0.223908

H -6.268413 0.608296 0.514943

C -1.882854 0.568472 2.761489

H -2.926930 0.497573 3.100229

H -1.876045 0.723606 1.674520

H -1.402243 1.429888 3.251846

C 2.443192 -0.907601 2.548670

H 3.492209 -1.115814 2.738285

C 5.095727 -1.280593 -0.586504

H 4.576345 -1.887177 -1.333836

C -0.152799 3.778420 -0.891332

C -1.174819 -0.928662 4.620516

H -0.660230 -0.112187 5.151027

H -0.728564 -1.887935 4.923981

H -2.224365 -0.937543 4.947989

C 4.355510 -0.368974 0.179525

C -2.444568 -2.845572 -1.120032

H -1.474812 -2.462026 -0.764321

C -4.416244 2.508506 -1.348586

H -3.947496 2.013669 -2.212068

H -4.175801 3.585282 -1.394352

H -5.509640 2.400859 -1.449163

C 1.368963 -1.015331 3.369903

H 1.316989 -1.325986 4.406450

C -0.882070 3.733438 0.455985

H -0.272082 4.214015 1.236871

H -1.055096 2.688330 0.744707

H -1.851111 4.251162 0.391653

C 5.071649 0.386454 1.126417

H 4.531548 1.109847 1.745950

C 1.990133 -0.724259 -2.490024

H 2.293236 0.292536 -2.756804

C -4.793585 -2.330925 -0.304763

H -5.037889 -3.391321 -0.419746

C -5.792780 -1.450932 0.117105

H -6.799792 -1.813905 0.338679

C 2.384275 3.457302 -0.855731

H 2.628374 4.487776 -1.085687

C -1.791865 -1.925701 2.404404

H -2.859760 -2.000890 2.658396

H -1.284992 -2.859206 2.697424

H -1.706855 -1.796920 1.319102

C -4.494435 2.626987 1.167865

H -5.597471 2.623121 1.174819

H -4.174963 3.682834 1.156895

H -4.151462 2.179624 2.111807

C 6.474130 -1.435300 -0.421836

H 7.017990 -2.157048 -1.037804

C 1.660347 -2.437787 -0.852324

H 1.693896 -2.780323 0.186137

C 7.157225 -0.673429 0.524208

H 8.236193 -0.791701 0.656308

C 3.203617 2.400689 -0.616435

H 4.288473 2.345121 -0.605411

C 6.447569 0.242854 1.301945

H 6.969934 0.847651 2.048764

C 1.512068 -1.575884 -3.486891

H 1.438823 -1.223076 -4.518955

C -1.010403 3.185509 -2.013569

H -0.449167 3.193456 -2.961083

H -1.926806 3.779784 -2.143035

H -1.294469 2.147532 -1.782143

B 2.750051 -0.173123 -0.003432

C -2.403367 -2.756280 -2.650160

H -1.630504 -3.420549 -3.068819

H -2.167373 -1.726358 -2.955964

H -3.377693 -3.039630 -3.083973

C 1.117302 -2.871855 -3.163292

H 0.735735 -3.540583 -3.939183

C 1.184817 -3.300251 -1.837742

H 0.847777 -4.305004 -1.569669

C -2.589846 -4.291699 -0.651072

H -3.482400 -4.783392 -1.074533

H -2.661682 -4.350641 0.446471

H -1.715102 -4.883199 -0.967804

C 0.191542 5.229923 -1.234876

H 0.799674 5.707004 -0.451018

H -0.744278 5.800363 -1.324243

H 0.724331 5.307112 -2.195211

**1^-^** (triplet)

Atom X Y Z

Fe 0.504830 0.489151 -0.086997

N -0.274222 -1.104227 -2.581549

N 2.130726 0.121733 0.353610

N -1.921861 -0.679615 -1.234678

N -1.049447 3.078553 0.556867

N -2.292326 1.327370 0.258849

C 3.335252 -0.470365 0.452168

C 4.545702 0.200071 0.066866

C 4.437604 1.641737 -0.390630

H 3.480775 1.707087 -0.934103

C -0.589280 -0.491045 -1.399789

C 1.093798 -1.221428 -3.149751

C -2.310155 -1.074521 1.317375

C -1.008855 1.757895 0.211283

C 3.448134 -1.828432 0.918308

C 5.770407 -0.461372 0.144284

H 6.685188 0.061089 -0.152464

C 1.769215 0.154244 -3.153563

H 2.746840 0.084741 -3.654425

H 1.939493 0.492965 -2.120835

H 1.148193 0.892226 -3.684855

C -2.427053 -1.434131 -2.267381

H -3.474579 -1.713181 -2.326206

C -5.218260 -0.988918 0.452123

H -4.842775 -1.580413 1.291816

C 0.152813 3.922889 0.755012

C 1.008986 -1.733141 -4.591851

H 0.389776 -1.075989 -5.222327

H 0.612574 -2.758186 -4.647952

H 2.023412 -1.752874 -5.014801

C -4.314466 -0.223807 -0.296820

C 2.190618 -2.515128 1.422189

H 1.363663 -2.169054 0.782025

C 4.317612 2.578741 0.816471

H 3.471855 2.265555 1.443557

H 4.151371 3.624864 0.504538

H 5.235649 2.544349 1.427140

C -1.404358 -1.702286 -3.117045

H -1.406859 -2.253410 -4.049447

C 1.075935 3.792379 -0.461185

H 0.535030 4.034288 -1.388743

H 1.468409 2.767381 -0.529705

H 1.929338 4.479068 -0.355693

C -4.849185 0.519009 -1.365778

H -4.173589 1.129168 -1.974132

C -2.342371 -0.555488 2.619890

H -2.558436 0.507885 2.764633

C 4.693946 -2.448245 0.962064

H 4.767385 -3.481180 1.315760

C 5.860944 -1.783037 0.579547

H 6.829343 -2.288708 0.624385

C -2.351386 3.451967 0.852024

H -2.636776 4.454687 1.147435

C 1.893172 -2.208046 -2.293894

H 2.919511 -2.309942 -2.677119

H 1.412192 -3.198839 -2.295274

H 1.950031 -1.837129 -1.263924

C 5.552594 2.097990 -1.330509

H 6.528119 2.156040 -0.818616

H 5.335060 3.104288 -1.725343

H 5.663652 1.411867 -2.184820

C -6.584125 -1.016511 0.155562

H -7.260086 -1.625695 0.762280

C -2.001070 -2.437331 1.186477

H -1.940621 -2.876069 0.185754

C -7.085336 -0.271017 -0.908963

H -8.153072 -0.289818 -1.144085

C -3.119937 2.348749 0.664446

H -4.192447 2.218572 0.773845

C -6.207983 0.501482 -1.673276

H -6.587210 1.091836 -2.512323

C -2.099161 -1.353984 3.737939

H -2.124621 -0.914162 4.738826

C 0.869878 3.447890 2.024681

H 0.200638 3.527229 2.895571

H 1.766939 4.058271 2.209580

H 1.180054 2.397901 1.907844

B -2.709905 -0.171533 0.021889

C 1.858226 -2.022707 2.836031

H 0.899448 -2.436925 3.185286

H 1.770492 -0.926780 2.831410

H 2.650886 -2.314054 3.546701

C -1.814606 -2.709548 3.580046

H -1.615933 -3.338373 4.451864

C -1.759711 -3.248305 2.294768

H -1.516094 -4.305132 2.154927

C 2.226499 -4.040252 1.361700

H 2.954757 -4.469993 2.070925

H 2.489184 -4.397714 0.352962

H 1.237605 -4.449844 1.624184

C -0.266390 5.387985 0.908897

H -0.840756 5.737808 0.037034

H 0.638022 6.007506 0.991261

H -0.863060 5.555932 1.818134

**1^-^** (quintet)

Atom X Y Z

Fe 0.483371 0.383804 -0.164186

N -0.329464 -2.004056 -2.135932

N 2.228939 0.512553 0.186777

N -1.990132 -0.991993 -1.174153

N -1.112160 3.140827 0.030680

N -2.349934 1.369304 -0.148469

C 3.378333 -0.113565 0.420051

C 4.569340 0.191029 -0.345326

C 4.464429 1.307861 -1.366488

H 3.467127 1.204367 -1.827144

C -0.639081 -1.009217 -1.267233

C 1.067691 -2.405527 -2.453836

C -2.321523 -0.727563 1.392982

C -1.066072 1.788283 -0.048467

C 3.506941 -1.145266 1.432676

C 5.757323 -0.492056 -0.109648

H 6.644594 -0.245818 -0.702618

C 1.837322 -1.188138 -2.976467

H 2.856113 -1.488387 -3.263297

H 1.928916 -0.424045 -2.189315

H 1.332467 -0.753169 -3.853167

C -2.520639 -1.987449 -1.964820

H -3.588217 -2.171704 -2.035459

C -5.262618 -0.814183 0.587675

H -4.874775 -1.163077 1.548746

C 0.103240 3.987762 0.147901

C 1.053166 -3.500673 -3.522493

H 0.565381 -3.161008 -4.449544

H 0.551758 -4.414898 -3.169484

H 2.091424 -3.766405 -3.765804

C -4.369187 -0.281801 -0.351189

C 2.287108 -1.459638 2.280633

H 1.413130 -1.433325 1.603034

C 4.473467 2.674215 -0.668104

H 3.680237 2.697443 0.093129

H 4.298462 3.497715 -1.383542

H 5.443656 2.848885 -0.172757

C -1.486197 -2.626100 -2.573490

H -1.497316 -3.453170 -3.273754

C 0.980226 3.771209 -1.091225

H 0.408652 3.964453 -2.012305

H 1.370803 2.742166 -1.104541

H 1.844960 4.450953 -1.060748

C -4.918611 0.150813 -1.572444

H -4.252489 0.571494 -2.332922

C -2.359476 0.086424 2.536446

H -2.662120 1.133514 2.435587

C 4.723203 -1.792875 1.623318

H 4.800803 -2.570336 2.390252

C 5.857667 -1.488791 0.865059

H 6.800500 -2.016274 1.032294

C -2.427386 3.572701 0.002753

H -2.722008 4.614128 0.059184

C 1.725204 -2.932690 -1.174068

H 2.757676 -3.252651 -1.377150

H 1.158558 -3.784748 -0.767033

H 1.773226 -2.139877 -0.416712

C 5.509145 1.257254 -2.480056

H 6.526934 1.448063 -2.099180

H 5.299866 2.025705 -3.243022

H 5.519376 0.274309 -2.977423

C -6.632606 -0.911463 0.329155

H -7.300213 -1.332828 1.085910

C -1.906652 -2.055529 1.578146

H -1.848445 -2.724353 0.714116

C -7.148968 -0.474233 -0.888530

H -8.220160 -0.548816 -1.094541

C -3.195313 2.455819 -0.105782

H -4.275643 2.358448 -0.151071

C -6.282428 0.060132 -1.844258

H -6.673350 0.406752 -2.805056

C -2.002788 -0.392905 3.796369

H -2.030266 0.274573 4.661937

C 0.872281 3.584605 1.412060

H 0.222334 3.647991 2.298496

H 1.729717 4.260462 1.554094

H 1.265894 2.559673 1.318347

B -2.765486 -0.158057 -0.072280

C 2.044021 -0.362985 3.325731

H 1.089408 -0.521832 3.854796

H 2.004697 0.617650 2.831009

H 2.861228 -0.349921 4.067083

C -1.591212 -1.716469 3.949731

H -1.292384 -2.093052 4.931352

C -1.544376 -2.547797 2.832062

H -1.210301 -3.583661 2.934422

C 2.301600 -2.838368 2.937358

H 3.089058 -2.920988 3.705792

H 2.471941 -3.635616 2.196173

H 1.337409 -3.029944 3.434903

C -0.309740 5.458602 0.238205

H -0.853692 5.788419 -0.660573

H 0.596384 6.074500 0.324943

H -0.933285 5.654618 1.124150

**2^-^** (triplet)

Atom X Y Z

Fe -0.603380 0.382790 0.104895

N 1.844172 -0.498210 -1.389208

N 0.315965 -0.023034 -2.847655

N 1.929278 -0.704638 1.175028

N 0.518584 -0.474164 2.809854

N -2.328555 -0.117910 0.254141

C 0.688313 -0.227743 1.466755

C 2.506942 -1.260669 2.293818

H 3.499170 -1.700413 2.279999

C 1.629551 -1.128619 3.315909

H 1.717699 -1.451694 4.345542

C -0.676081 -0.167305 3.640715

C -1.710563 -1.282620 3.470287

H -2.579961 -1.095358 4.119440

H -1.276959 -2.258138 3.738581

H -2.058943 -1.310159 2.429750

C -0.251917 -0.072457 5.114068

H -1.115715 0.260552 5.706792

H 0.561303 0.656763 5.252919

H 0.065763 -1.041296 5.527109

C -1.286005 1.173889 3.228467

H -2.047623 1.463567 3.969025

H -1.776473 1.076434 2.251478

H -0.519415 1.962057 3.181010

C 0.609768 0.054004 -1.508714

C 2.301912 -0.931352 -2.611336

H 3.270563 -1.404722 -2.737772

C 1.346552 -0.644533 -3.529572

H 1.331643 -0.838395 -4.595119

C -0.937055 0.449658 -3.491322

C -2.061423 -0.550950 -3.211105

H -3.001290 -0.208751 -3.669965

H -2.219150 -0.640626 -2.127677

H -1.813308 -1.541574 -3.621348

C -0.723120 0.577853 -5.004136

H -1.627927 1.017556 -5.447661

H -0.566764 -0.397714 -5.488156

H 0.128092 1.234763 -5.241501

C -1.292901 1.828301 -2.932277

H -2.190140 2.215157 -3.437953

H -0.463198 2.537398 -3.076830

H -1.502879 1.754912 -1.860140

C 3.981888 -1.435087 -0.247639

C 5.327714 -1.053653 -0.316857

H 5.581665 0.009535 -0.274112

C 6.355128 -1.994226 -0.439243

H 7.395618 -1.660900 -0.490622

C 6.055956 -3.353416 -0.495379

H 6.855918 -4.092719 -0.591120

C 4.721932 -3.762315 -0.427487

H 4.472041 -4.826046 -0.469962

C 3.709048 -2.814075 -0.305603

H 2.668048 -3.148053 -0.252394

C 3.268919 1.182108 -0.012430

C 3.542587 1.787399 1.224737

H 3.401505 1.210264 2.143574

C 3.958110 3.116070 1.323374

H 4.146856 3.556032 2.306680

C 4.118575 3.887325 0.171999

H 4.437158 4.930957 0.243537

C 3.863832 3.309064 -1.071906

H 3.978623 3.901590 -1.983987

C 3.449019 1.979166 -1.154552

H 3.233903 1.554519 -2.139915

C 0.327371 3.363912 0.318559

H 1.299102 2.874406 0.224726

C 0.224227 4.749659 0.411926

H 1.131783 5.355463 0.393173

C -1.041323 5.320649 0.526734

H -1.160018 6.404610 0.601468

C -2.153548 4.480037 0.542434

H -3.166614 4.877586 0.626958

C -3.161989 -1.160550 0.091572

C -2.749802 -2.550261 0.070774

C -3.678849 -3.559167 -0.177712

H -3.332590 -4.598541 -0.209992

C -5.029223 -3.295290 -0.401420

H -5.734137 -4.106492 -0.601976

C -5.460077 -1.967926 -0.349386

H -6.522520 -1.752905 -0.503053

C -4.579474 -0.921431 -0.097501

C -1.288573 -2.919438 0.270397

H -0.806171 -2.033768 0.707888

C -0.576392 -3.196518 -1.057748

H 0.498000 -3.389970 -0.901542

H -0.659273 -2.337277 -1.733804

H -1.015361 -4.073706 -1.563634

C -1.074460 -4.087055 1.237943

H -0.004951 -4.189557 1.487664

H -1.403642 -5.047013 0.804569

H -1.633632 -3.938558 2.173499

C -5.058220 0.515795 -0.016835

H -4.427023 0.975796 0.764073

C -4.767811 1.275666 -1.315998

H -5.025928 2.346648 -1.229588

H -5.346659 0.850622 -2.153752

H -3.699380 1.193272 -1.552838

C -6.523114 0.678358 0.388824

H -6.758019 1.741205 0.567443

H -6.751348 0.117306 1.308511

H -7.211559 0.324991 -0.397584

B 2.748045 -0.369157 -0.117992

C -1.956706 3.106376 0.442682

N -0.739945 2.561186 0.335882

H -2.777043 2.379232 0.440053

**2^-^** (quintet)

Atom X Y Z

Fe -0.589792 0.404051 -0.244878

N 2.155036 -0.414105 -1.252935

N 0.969387 0.022747 -3.015476

N 1.716977 -0.767900 1.308385

N 0.037205 -0.706178 2.679125

N -2.311817 -0.102324 -0.618593

C 0.471283 -0.247195 1.472567

C 2.044748 -1.549643 2.395439

H 2.997880 -2.060342 2.483505

C 0.995332 -1.522258 3.253144

H 0.862039 -2.019633 4.207389

C -1.284023 -0.455443 3.310420

C -2.175320 -1.684813 3.104749

H -3.148917 -1.534167 3.594610

H -1.707785 -2.587113 3.529731

H -2.365792 -1.857819 2.036296

C -1.062072 -0.196512 4.808624

H -2.018971 0.085352 5.271445

H -0.346450 0.625997 4.961499

H -0.692434 -1.084798 5.342044

C -1.943650 0.777618 2.697324

H -2.884607 0.975969 3.232307

H -2.187483 0.617457 1.635894

H -1.293664 1.661246 2.784160

C 0.962359 0.095166 -1.654834

C 2.896598 -0.793401 -2.351927

H 3.893149 -1.214408 -2.267988

C 2.157976 -0.531103 -3.458531

H 2.393205 -0.697274 -4.502914

C -0.154902 0.437860 -3.894595

C -1.277081 -0.600607 -3.804193

H -2.077669 -0.350795 -4.518193

H -1.706483 -0.603424 -2.786920

H -0.894250 -1.603936 -4.045629

C 0.344432 0.547688 -5.339365

H -0.472695 0.934657 -5.964616

H 0.635243 -0.429985 -5.752347

H 1.196886 1.239561 -5.425848

C -0.669117 1.806373 -3.439943

H -1.453881 2.151924 -4.129778

H 0.143898 2.548585 -3.420672

H -1.103823 1.733013 -2.437140

C 4.052422 -1.302721 0.283488

C 5.346097 -0.852330 0.574225

H 5.508541 0.212459 0.763990

C 6.436587 -1.725901 0.627701

H 7.433320 -1.338744 0.857525

C 6.256170 -3.085948 0.388761

H 7.105918 -3.772800 0.429801

C 4.976534 -3.563408 0.095248

H 4.819238 -4.628673 -0.094837

C 3.899810 -2.681462 0.044427

H 2.902786 -3.070015 -0.186567

C 3.144236 1.249941 0.497215

C 3.233426 1.742782 1.809687

H 3.018221 1.070398 2.646038

C 3.561754 3.071437 2.080741

H 3.610492 3.420919 3.115888

C 3.815344 3.957084 1.032258

H 4.065600 5.001551 1.237516

C 3.742363 3.492597 -0.280715

H 3.931512 4.175863 -1.113285

C 3.415286 2.159794 -0.536101

H 3.347207 1.823265 -1.574814

C 0.123167 3.351586 0.520185

H 1.071012 2.872765 0.775847

C -0.066191 4.718946 0.701150

H 0.751341 5.324252 1.096191

C -1.301069 5.273405 0.371151

H -1.485636 6.342572 0.503083

C -2.297319 4.435526 -0.127612

H -3.283464 4.818687 -0.395852

C -3.141082 -1.098128 -0.321555

C -2.778601 -2.505481 -0.417887

C -3.669747 -3.500418 -0.028677

H -3.351607 -4.548371 -0.098478

C -4.947547 -3.217195 0.457572

H -5.625436 -4.017637 0.764939

C -5.335566 -1.875286 0.534338

H -6.337827 -1.642509 0.909360

C -4.492003 -0.837477 0.157192

C -1.398076 -2.892615 -0.922752

H -0.927916 -1.956721 -1.259733

C -1.454606 -3.837223 -2.127176

H -0.448478 -3.998729 -2.551956

H -2.101388 -3.425652 -2.916866

H -1.859819 -4.825539 -1.849786

C -0.500818 -3.470814 0.176955

H 0.513769 -3.679030 -0.204537

H -0.913829 -4.413982 0.574428

H -0.401520 -2.769581 1.016684

C -4.925427 0.615029 0.226561

H -4.057493 1.165798 0.626602

C -5.181413 1.171134 -1.180495

H -5.373574 2.259642 -1.161097

H -6.055697 0.678551 -1.638372

H -4.299497 0.977911 -1.809509

C -6.112811 0.893442 1.146245

H -6.291437 1.978842 1.228666

H -5.937890 0.497810 2.159442

H -7.044127 0.439307 0.767197

B 2.752769 -0.311364 0.205679

N -0.831589 2.552588 0.035235

C -2.020448 3.080573 -0.280977

H -2.747569 2.351444 -0.656279

**3^-^** (triplet)

Atom X Y Z

Fe -0.558367 0.442184 0.158073

N 1.935563 -0.047080 -1.337131

N 0.519046 0.873773 -2.700793

N 1.885807 -1.110060 1.034188

N 0.426864 -1.367332 2.613428

N -2.269423 2.787911 0.328271

N -2.425086 -0.172007 0.248108

H -3.003323 0.632378 0.468895

C 0.639669 -0.739803 1.418641

C 2.431706 -1.975826 1.954657

H 3.427673 -2.393184 1.845042

C 1.520686 -2.147838 2.944244

H 1.577266 -2.756113 3.838767

C -0.769911 -1.198968 3.479474

C -1.902866 -2.091950 2.969134

H -2.794799 -1.970145 3.603045

H -1.605914 -3.152118 2.991449

H -2.171093 -1.806023 1.944935

C -0.412075 -1.591375 4.918890

H -1.274110 -1.374394 5.565647

H 0.449297 -1.016828 5.292790

H -0.194532 -2.665372 5.019366

C -1.196418 0.272106 3.470397

H -2.005959 0.421671 4.201126

H -1.571200 0.568621 2.483956

H -0.352909 0.927915 3.733125

C 0.725755 0.570229 -1.380203

C 2.480334 -0.114181 -2.599241

H 3.448685 -0.563189 -2.793683

C 1.601210 0.457435 -3.456281

H 1.666790 0.576989 -4.530155

C -0.606649 1.675215 -3.253916

C -1.951527 1.151692 -2.749112

H -2.762559 1.726834 -3.220935

H -2.058683 1.253620 -1.664265

H -2.088683 0.090351 -3.002242

C -0.602915 1.574203 -4.784899

H -1.481263 2.111161 -5.169803

H -0.673942 0.528088 -5.119989

H 0.286738 2.036739 -5.238162

C -0.395274 3.137521 -2.843525

H -1.206363 3.765747 -3.242415

H 0.564433 3.510828 -3.234873

H -0.387041 3.235223 -1.751337

C 4.032904 -1.282167 -0.413827

C 5.363612 -0.885546 -0.231938

H 5.569372 0.104195 0.185735

C 6.433480 -1.720156 -0.569124

H 7.460688 -1.378328 -0.413559

C 6.194070 -2.984870 -1.101426

H 7.027862 -3.640735 -1.366363

C 4.876309 -3.407098 -1.292525

H 4.673121 -4.397648 -1.708805

C 3.820527 -2.564885 -0.951549

H 2.792877 -2.909243 -1.107125

C 3.191653 1.103141 0.614092

C 3.245812 1.325291 1.998416

H 2.990678 0.510734 2.682825

C 3.588531 2.567364 2.535037

H 3.602273 2.706206 3.619667

C 3.896077 3.634240 1.692114

H 4.153435 4.612330 2.107294

C 3.860354 3.439467 0.311328

H 4.088144 4.268757 -0.364094

C 3.514505 2.193333 -0.210191

H 3.469439 2.072796 -1.297125

C -1.005502 2.322275 0.458925

C 0.001434 3.209533 0.920000

H 1.025946 2.844668 1.028593

C -0.295116 4.531597 1.214873

H 0.490673 5.209164 1.563921

C -1.609522 4.983441 1.051500

H -1.894822 6.016957 1.262375

C -2.551243 4.060638 0.609433

H -3.597777 4.366327 0.473256

C -3.145840 -1.208532 -0.250707

C -2.518871 -2.343677 -0.867319

C -3.296076 -3.389752 -1.361395

H -2.789101 -4.241147 -1.828757

C -4.685716 -3.388310 -1.285732

H -5.273170 -4.220496 -1.680703

C -5.307474 -2.291578 -0.688195

H -6.398660 -2.280147 -0.620345

C -4.584976 -1.220278 -0.171923

C -1.008210 -2.432887 -1.016404

H -0.583443 -1.493564 -0.640963

C -0.545063 -2.541240 -2.471783

H 0.553678 -2.472326 -2.533197

H -0.962044 -1.725793 -3.079843

H -0.854173 -3.496419 -2.928292

C -0.389847 -3.543147 -0.163980

H 0.710347 -3.514640 -0.221117

H -0.727325 -4.538464 -0.498944

H -0.668881 -3.428710 0.891621

C -5.296780 -0.034102 0.462981

H -4.679318 0.287884 1.321006

C -5.387656 1.155374 -0.506731

H -5.879901 2.018496 -0.027488

H -5.975096 0.871982 -1.395782

H -4.392276 1.488065 -0.833879

C -6.680045 -0.361881 1.028731

H -7.068753 0.494883 1.602438

H -6.646899 -1.236754 1.696289

H -7.410883 -0.573791 0.230982

B 2.755059 -0.338317 -0.027593

**3^-^** (quintet)

Atom X Y Z

Fe -0.548752 0.660173 0.021604

N 1.905208 -0.575478 -1.305345

N 0.495040 -0.224322 -2.911926

N 1.886242 -0.644402 1.311773

N 0.449009 -0.381015 2.911557

N -2.319617 2.979624 0.226478

N -2.351244 -0.208107 0.146756

H -2.956722 0.604946 0.236909

C 0.669885 -0.115143 1.592885

C 2.413480 -1.239611 2.436165

H 3.388460 -1.715749 2.444825

C 1.514339 -1.089004 3.440194

H 1.561897 -1.427350 4.468231

C -0.753691 0.017093 3.689914

C -1.856645 -1.026232 3.490742

H -2.757633 -0.733734 4.051809

H -1.533893 -2.015834 3.849791

H -2.119890 -1.091482 2.426204

C -0.380480 0.109095 5.175540

H -1.245483 0.494141 5.734071

H 0.464628 0.796370 5.334452

H -0.127620 -0.870384 5.607977

C -1.245237 1.390201 3.225454

H -2.016195 1.752036 3.922696

H -1.697877 1.343416 2.229088

H -0.424009 2.120847 3.191611

C 0.690402 -0.039373 -1.574690

C 2.460098 -1.088521 -2.456355

H 3.437348 -1.559634 -2.475691

C 1.580843 -0.875221 -3.467657

H 1.654106 -1.143537 -4.514477

C -0.674215 0.295196 -3.671156

C -1.949455 -0.414527 -3.212422

H -2.821914 0.015327 -3.727751

H -2.095514 -0.298194 -2.129321

H -1.910512 -1.489187 -3.443941

C -0.469636 0.046091 -5.168589

H -1.337704 0.449206 -5.709337

H -0.405504 -1.027621 -5.401743

H 0.430076 0.551076 -5.552472

C -0.777162 1.804664 -3.426747

H -1.599733 2.226598 -4.024079

H 0.160607 2.307618 -3.709456

H -0.971425 2.023634 -2.368542

C 4.041592 -1.277114 0.003627

C 5.353247 -0.788221 0.043635

H 5.515157 0.292696 0.083448

C 6.458976 -1.643751 0.034179

H 7.469668 -1.227095 0.066570

C 6.276575 -3.023708 -0.016252

H 7.138701 -3.696177 -0.023894

C 4.979147 -3.539691 -0.056856

H 4.820065 -4.620853 -0.096376

C 3.887045 -2.675078 -0.046572

H 2.876060 -3.094654 -0.078229

C 3.107180 1.282711 0.054700

C 3.234917 1.987703 1.261376

H 3.044397 1.469256 2.206038

C 3.578721 3.339509 1.295666

H 3.653961 3.857913 2.255507

C 3.810746 4.033440 0.108871

H 4.070154 5.095266 0.129515

C 3.692793 3.358054 -1.105641

H 3.859691 3.890693 -2.046060

C 3.346432 2.006807 -1.124222

H 3.245314 1.503198 -2.090737

C -0.997470 2.704710 0.047560

C -0.092932 3.785386 -0.055500

H 0.970875 3.571910 -0.197291

C -0.530075 5.103442 0.024083

H 0.179779 5.933782 -0.052302

C -1.892316 5.346679 0.206183

H -2.292096 6.361784 0.274961

C -2.737193 4.242514 0.300665

H -3.816224 4.391608 0.445060

C -3.056401 -1.363990 0.028816

C -2.402434 -2.632970 -0.112350

C -3.161072 -3.795464 -0.235793

H -2.637412 -4.752040 -0.340242

C -4.553120 -3.783543 -0.228056

H -5.124745 -4.709563 -0.326003

C -5.199300 -2.555812 -0.086820

H -6.292262 -2.535924 -0.076726

C -4.495593 -1.361245 0.041220

C -0.885254 -2.745291 -0.118686

H -0.484208 -1.728452 -0.068173

C -0.333018 -3.363145 -1.405808

H 0.768759 -3.331283 -1.408680

H -0.679555 -2.810555 -2.289501

H -0.642989 -4.415675 -1.520103

C -0.341428 -3.484198 1.107289

H 0.760845 -3.465276 1.116271

H -0.667245 -4.538299 1.121019

H -0.686682 -3.011852 2.035980

C -5.224844 -0.029915 0.163874

H -4.680629 0.574939 0.911694

C -5.189771 0.751395 -1.159137

H -5.647671 1.747577 -1.045338

H -5.741442 0.199564 -1.938236

H -4.158502 0.899250 -1.508028

C -6.662498 -0.141742 0.673144

H -7.075154 0.861192 0.867545

H -6.716847 -0.722381 1.606951

H -7.323328 -0.626019 -0.064600

B 2.724419 -0.307738 0.018703

**3^γ-^** (triplet)

Atom X Y Z

Fe -0.559505 0.514246 0.128367

N 1.929874 -0.137151 -1.329903

N 0.557468 0.770832 -2.744186

N 1.812948 -1.112046 1.065247

N 0.322135 -1.260845 2.627750

N -2.455856 -0.111912 0.178080

H -3.064288 0.635631 0.487430

C 0.575400 -0.685283 1.413663

C 2.312122 -1.964707 2.022261

H 3.293941 -2.420396 1.941893

C 1.380919 -2.068870 3.002039

H 1.401527 -2.646153 3.918245

C -0.878371 -1.028083 3.472750

C -2.018633 -1.930736 2.996908

H -2.917488 -1.763093 3.610246

H -1.737138 -2.992120 3.079824

H -2.267446 -1.702668 1.954330

C -0.540276 -1.352226 4.934162

H -1.401809 -1.081952 5.561161

H 0.331091 -0.777907 5.284000

H -0.348210 -2.423759 5.093621

C -1.276944 0.448031 3.390439

H -2.096390 0.645239 4.098262

H -1.628457 0.712134 2.387006

H -0.427226 1.100892 3.638873

C 0.738902 0.510741 -1.409435

C 2.488598 -0.265074 -2.580700

H 3.446994 -0.746092 -2.744748

C 1.636931 0.298770 -3.469210

H 1.719369 0.380078 -4.545412

C -0.526318 1.594007 -3.344835

C -1.895100 1.127395 -2.850388

H -2.677609 1.767214 -3.285744

H -1.980605 1.172059 -1.759900

H -2.091883 0.087092 -3.149034

C -0.504184 1.444269 -4.871674

H -1.362535 1.992709 -5.284843

H -0.598935 0.390772 -5.176345

H 0.403426 1.869414 -5.325502

C -0.263340 3.059417 -2.978621

H -1.042173 3.707473 -3.409071

H 0.715309 3.378859 -3.370286

H -0.264433 3.193426 -1.890543

C 3.976979 -1.409788 -0.339750

C 5.316412 -1.053163 -0.138554

H 5.546541 -0.059224 0.255564

C 6.364193 -1.932643 -0.427111

H 7.398704 -1.620989 -0.257558

C 6.093563 -3.203524 -0.928924

H 6.910034 -3.894507 -1.155690

C 4.766797 -3.586279 -1.139098

H 4.538960 -4.580863 -1.532336

C 3.733246 -2.699338 -0.846667

H 2.698669 -3.013426 -1.018237

C 3.209625 1.033125 0.597065

C 3.301149 1.276124 1.976017

H 3.040532 0.479850 2.679846

C 3.687573 2.517296 2.483646

H 3.729018 2.674229 3.565044

C 4.001725 3.561848 1.615586

H 4.289911 4.540621 2.007764

C 3.929550 3.345423 0.239786

H 4.159670 4.158417 -0.454117

C 3.541478 2.100076 -0.252902

H 3.468984 1.963421 -1.336362

C -0.927591 2.451330 0.352751

C 0.114168 3.237625 0.892512

H 1.101399 2.801784 1.075573

C -0.077525 4.584162 1.190255

H 0.753758 5.171772 1.602613

C -2.229905 4.514529 0.500617

H -3.180019 5.044671 0.344393

C -3.181138 -1.187316 -0.235460

C -2.559824 -2.342108 -0.816968

C -3.337329 -3.424376 -1.224800

H -2.832851 -4.290966 -1.665545

C -4.722139 -3.444686 -1.089785

H -5.308439 -4.307240 -1.414618

C -5.339439 -2.329679 -0.525157

H -6.426979 -2.331193 -0.414000

C -4.615540 -1.219577 -0.099873

C -1.053697 -2.428011 -0.998989

H -0.629394 -1.468353 -0.681753

C -0.624865 -2.606422 -2.457672

H 0.472078 -2.540626 -2.547504

H -1.057312 -1.820774 -3.093973

H -0.942862 -3.582157 -2.861741

C -0.418141 -3.494925 -0.104871

H 0.680138 -3.469180 -0.182479

H -0.760301 -4.505727 -0.383750

H -0.677702 -3.328618 0.949389

C -5.330483 0.001197 0.459459

H -4.697416 0.413874 1.265578

C -5.475946 1.082910 -0.622728

H -5.850301 2.032034 -0.204597

H -6.180970 0.743086 -1.398756

H -4.513954 1.280714 -1.117653

C -6.685638 -0.291736 1.104992

H -7.074886 0.612184 1.600299

H -6.606832 -1.090530 1.858185

H -7.437444 -0.601635 0.361194

B 2.727620 -0.409325 -0.004949

C -2.129647 3.161968 0.172907

H -3.014234 2.671182 -0.247460

N -1.228910 5.237608 1.002237

**3^γ-^** (quintet)

Atom X Y Z

Fe -0.582886 0.705402 0.020803

N 1.810058 -0.648103 -1.318504

N 0.388418 -0.279921 -2.910996

N 1.827802 -0.669251 1.301137

N 0.444969 -0.281660 2.922834

N -2.415514 -0.133158 0.221039

H -3.037649 0.654378 0.369217

C 0.649099 -0.065819 1.591887

C 2.346521 -1.261219 2.430982

H 3.293457 -1.790949 2.431899

C 1.481231 -1.031837 3.449484

H 1.534253 -1.342057 4.486042

C -0.719887 0.193939 3.713742

C -1.873744 -0.801560 3.561635

H -2.748948 -0.456481 4.133739

H -1.587241 -1.795623 3.938536

H -2.159105 -0.882045 2.503584

C -0.314418 0.305603 5.189424

H -1.146671 0.751578 5.752374

H 0.568917 0.950283 5.314123

H -0.105988 -0.674395 5.643614

C -1.148825 1.578997 3.224895

H -1.914037 1.982770 3.904955

H -1.581845 1.537408 2.219707

H -0.298204 2.274876 3.191030

C 0.605497 -0.083613 -1.578050

C 2.337436 -1.185249 -2.471497

H 3.302239 -1.680813 -2.497452

C 1.451571 -0.960675 -3.473722

H 1.505597 -1.240800 -4.518494

C -0.774533 0.260050 -3.664945

C -0.630130 -0.077028 -5.152141

H -1.500193 0.330634 -5.686179

H -0.613324 -1.163531 -5.327084

H 0.273043 0.371848 -5.592863

C -0.792223 1.783178 -3.497609

H -1.620698 2.217310 -4.077890

H 0.153659 2.221471 -3.851565

H -0.920928 2.067666 -2.445238

C -2.068328 -0.361831 -3.136543

H -2.932918 0.075764 -3.658708

H -2.183651 -0.179954 -2.059264

H -2.082793 -1.449242 -3.302166

C 3.906213 -1.488524 -0.030533

C 5.252081 -1.101290 -0.032814

H 5.497127 -0.035518 -0.021486

C 6.288679 -2.039437 -0.049569

H 7.328626 -1.700930 -0.051045

C 6.000398 -3.402045 -0.064719

H 6.808225 -4.138753 -0.077798

C 4.666773 -3.817324 -0.063608

H 4.424327 -4.883527 -0.075978

C 3.644780 -2.871093 -0.047137

H 2.604782 -3.212981 -0.047466

C 3.179136 1.133467 0.000946

C 3.495537 1.787432 1.202509

H 3.363600 1.257142 2.150851

C 3.955464 3.104001 1.229345

H 4.178298 3.584135 2.186185

C 4.113556 3.814186 0.039512

H 4.459227 4.851043 0.055017

C 3.808074 3.189517 -1.168587

H 3.911856 3.737886 -2.108723

C 3.351325 1.871187 -1.180102

H 3.105033 1.409668 -2.141133

C -0.827512 2.793981 -0.017946

C 0.243421 3.691781 0.165320

H 1.263191 3.318229 0.302450

C 0.038789 5.071084 0.176549

H 0.891511 5.748879 0.319718

C -2.186731 4.824287 -0.156963

H -3.169821 5.298419 -0.287386

C -3.139449 -1.284929 0.108960

C -2.504408 -2.559376 -0.045871

C -3.279189 -3.711848 -0.164753

H -2.768724 -4.673974 -0.278814

C -4.670225 -3.683735 -0.137058

H -5.254049 -4.602295 -0.231273

C -5.298976 -2.450214 0.019326

H -6.391051 -2.416860 0.044398

C -4.577596 -1.264972 0.141563

C -0.990304 -2.697131 -0.056300

H -0.568505 -1.687855 -0.042740

C -0.455707 -3.369248 -1.323276

H 0.645749 -3.351099 -1.335219

H -0.801299 -2.844402 -2.224695

H -0.777789 -4.421474 -1.397945

C -0.464017 -3.402266 1.196899

H 0.637824 -3.413470 1.206543

H -0.819959 -4.445093 1.251600

H -0.795790 -2.883554 2.105994

C -5.296471 0.071923 0.267230

H -4.743561 0.675149 1.011154

C -5.271878 0.840351 -1.063784

H -5.694877 1.852561 -0.953260

H -5.863114 0.299010 -1.820154

H -4.247683 0.938660 -1.450827

C -6.728548 -0.023024 0.795293

H -7.130440 0.984733 0.986352

H -6.775249 -0.595576 1.734162

H -7.401630 -0.507522 0.069498

B 2.667440 -0.418814 -0.012117

C -2.071743 3.433578 -0.183735

N -1.154785 5.650061 0.020205

H -2.986043 2.847593 -0.345635

**1-K^’^**

Atom X Y Z

Fe -0.542814 0.534474 -0.445911

K -0.391257 0.563203 2.728453

N 1.870924 -1.068983 -1.230833

N 0.210992 -1.895768 -2.358219

N 2.370157 1.250649 -0.153255

N 1.294813 3.128176 -0.001595

N -1.999016 0.414970 0.621702

B 2.697300 -0.303365 -0.120964

C 0.555215 -0.904965 -1.500424

C 2.341237 -2.175481 -1.904647

H 3.373848 -2.503184 -1.838969

C 1.305972 -2.698168 -2.613094

H 1.281320 -3.555385 -3.275698

C -1.188355 -2.173623 -2.782864

C -1.178162 -3.123322 -3.982823

H -0.782937 -4.116155 -3.722744

H -2.210559 -3.266791 -4.330110

H -0.590542 -2.713189 -4.818248

C -1.917370 -2.814513 -1.597973

H -1.906643 -2.134883 -0.735430

H -2.970564 -3.011434 -1.846108

H -1.435532 -3.763130 -1.313780

C -1.875565 -0.866114 -3.184684

H -1.307565 -0.345152 -3.969895

H -2.884966 -1.085666 -3.560206

H -1.997431 -0.190024 -2.326590

C 1.125853 1.781098 -0.079126

C 3.306679 2.257728 -0.103833

H 4.374083 2.063839 -0.137114

C 2.641185 3.439361 -0.013618

H 3.028825 4.449707 0.041254

C 0.168992 4.098193 0.015643

C -0.580084 4.002082 -1.318272

H -1.027175 3.005485 -1.447737

H -1.396826 4.737872 -1.350170

H 0.100355 4.192271 -2.161365

C -0.768976 3.757835 1.178873

H -0.212266 3.768521 2.131084

H -1.569653 4.508544 1.245756

H -1.250207 2.777775 1.023997

C 0.712249 5.517219 0.195234

H 1.277356 5.621864 1.133669

H 1.355207 5.821491 -0.643710

H -0.133186 6.217782 0.233916

C 4.280528 -0.518813 -0.442781

C 4.805492 -0.074811 -1.671155

H 4.140993 0.422503 -2.385276

C 6.144317 -0.253061 -2.009581

H 6.517727 0.105847 -2.972028

C 7.008005 -0.895394 -1.119864

H 8.059372 -1.040293 -1.379696

C 6.514154 -1.353874 0.098188

H 7.178257 -1.864861 0.799868

C 5.168329 -1.165800 0.426092

H 4.804956 -1.542669 1.385821

C 2.267256 -0.848346 1.361054

C 1.434749 -1.958139 1.570917

H 1.031465 -2.498916 0.710529

C 1.086859 -2.389940 2.855199

H 0.427672 -3.252681 2.976143

C 1.577166 -1.722432 3.978348

H 1.318622 -2.066089 4.982902

C 2.416718 -0.617148 3.800463

H 2.822408 -0.092855 4.670101

C 2.744958 -0.191383 2.511602

H 3.400385 0.677719 2.393634

C -3.219678 -0.147164 0.434111

C -4.193764 0.426413 -0.461663

C -5.422545 -0.196500 -0.663452

H -6.145810 0.255498 -1.347842

C -5.765732 -1.383044 -0.015514

H -6.736113 -1.853266 -0.188272

C -4.844114 -1.950662 0.861015

H -5.107363 -2.880906 1.372914

C -3.599305 -1.370234 1.093804

C -3.864810 1.745267 -1.134324

H -2.780708 1.717405 -1.338855

C -4.095225 2.908620 -0.162659

H -3.567886 2.719018 0.782870

H -3.735924 3.865336 -0.579521

H -5.169353 3.015749 0.059845

C -4.576869 1.989555 -2.462981

H -4.436034 1.145919 -3.156072

H -5.660970 2.137604 -2.331223

H -4.184580 2.898387 -2.947248

C -2.615100 -2.002633 2.060657

H -1.615123 -1.715229 1.691589

C -2.794562 -1.417925 3.470776

H -3.725936 -1.791774 3.924337

H -1.964681 -1.695297 4.147986

H -2.895612 -0.320393 3.435441

C -2.653431 -3.529790 2.111121

H -1.844549 -3.919780 2.750778

H -3.599964 -3.907519 2.529119

H -2.534345 -3.962829 1.107042

**2-K^α^**

Atom X Y Z

Fe -0.404385 0.546772 0.054093

N 2.066132 -0.365087 -1.441307

N 0.736895 0.405800 -2.968711

N 2.035774 -0.937556 1.116963

N 0.624644 -1.003590 2.756577

N -2.211368 -0.228097 -0.553992

H -2.708270 0.522143 -1.027811

C 0.814329 -0.499201 1.499403

C 2.597832 -1.704092 2.113121

H 3.583922 -2.146497 2.022313

C 1.719704 -1.753817 3.143792

H 1.802456 -2.257069 4.099125

C -0.564934 -0.750816 3.599373

C -1.773886 -1.426687 2.947945

H -2.696087 -1.208857 3.512844

H -1.655436 -2.518224 2.932102

H -1.876106 -1.115116 1.900595

C -0.364935 -1.336704 4.999561

H -1.253164 -1.111200 5.607054

H 0.506515 -0.896247 5.505293

H -0.250687 -2.430073 4.976337

C -0.761449 0.764370 3.724134

H -1.650799 0.986519 4.337203

H -0.854172 1.247160 2.741230

H 0.107801 1.225142 4.215366

C 0.888589 0.280299 -1.619522

C 2.630482 -0.660803 -2.661790

H 3.586505 -1.164425 -2.757055

C 1.798998 -0.191486 -3.623377

H 1.894181 -0.231473 -4.701669

C -0.379719 1.078335 -3.679632

C 0.130705 1.625084 -5.020167

H -0.663859 2.224558 -5.486361

H 0.389046 0.826154 -5.729625

H 1.009843 2.271032 -4.878364

C -0.886947 2.260338 -2.851899

H -1.659846 2.797509 -3.421147

H -0.070332 2.958563 -2.617978

H -1.325018 1.947132 -1.898126

C -1.499174 0.063420 -3.927432

H -2.323341 0.533394 -4.485753

H -1.882513 -0.311640 -2.968861

H -1.128815 -0.790497 -4.514483

C 4.175992 -1.362447 -0.294417

C 5.498936 -0.921829 -0.168702

H 5.690673 0.127543 0.071734

C 6.581466 -1.789095 -0.342424

H 7.602314 -1.412641 -0.237420

C 6.362487 -3.129774 -0.648645

H 7.206499 -3.810350 -0.785523

C 5.052710 -3.596954 -0.779464

H 4.867253 -4.647070 -1.019829

C 3.983715 -2.721937 -0.603406

H 2.962676 -3.104235 -0.709808

C 3.304060 1.163583 0.252346

C 3.393721 1.639312 1.569285

H 3.168631 0.965887 2.401873

C 3.749755 2.957699 1.857008

H 3.798831 3.294290 2.895855

C 4.035787 3.847058 0.822602

H 4.312587 4.881222 1.042341

C 3.964346 3.399661 -0.496887

H 4.185282 4.085087 -1.319366

C 3.604324 2.079902 -0.769170

H 3.544689 1.757671 -1.813456

C -3.017348 -1.339172 -0.445385

C -2.440324 -2.622202 -0.168931

C -3.269613 -3.710885 0.078210

H -2.818247 -4.681581 0.295510

C -4.660747 -3.609065 0.036323

H -5.289467 -4.479294 0.233936

C -5.227844 -2.387632 -0.299073

H -6.315256 -2.316080 -0.380997

C -4.444954 -1.254172 -0.548143

C -0.931681 -2.792337 -0.239218

H -0.484414 -1.880618 0.175542

C -0.460805 -2.885587 -1.695200

H 0.639353 -2.893196 -1.749419

H -0.812409 -2.024596 -2.275908

H -0.842827 -3.803995 -2.169839

C -0.366787 -3.954802 0.575593

H 0.731703 -3.885514 0.604244

H -0.623532 -4.933441 0.138338

H -0.726104 -3.947480 1.615616

C -5.120722 0.023229 -1.041905

H -4.484526 0.893443 -0.794870

C -5.224984 0.015772 -2.573296

H -5.654123 0.956798 -2.953428

H -5.865207 -0.816559 -2.904328

H -4.236077 -0.123554 -3.031289

C -6.487821 0.301905 -0.408014

H -6.851057 1.300333 -0.696347

H -6.455918 0.251063 0.693764

H -7.246462 -0.423871 -0.736595

B 2.890971 -0.379044 -0.093713

C -2.175762 4.353041 1.270138

H -3.179317 4.680351 1.571481

C -1.150586 5.284024 1.167131

H -1.334623 6.338383 1.383005

C 0.108675 4.817889 0.786543

H 0.956768 5.502614 0.697491

C 0.266720 3.463596 0.521171

H 1.247132 3.088553 0.216383

N -2.021658 3.045151 1.024347

C -0.809014 2.553658 0.634070

K -3.525800 0.923240 1.660746

**2-K^β^**

Atom X Y Z

Fe -0.393812 0.468653 -0.158730

K -2.816631 2.741553 1.057806

N 2.217685 -0.154716 1.204376

N 1.139966 0.953398 2.721374

N 1.819453 -1.339189 -1.093047

N 0.126180 -1.928646 -2.308664

N -2.321701 0.011367 0.510293

B 2.817233 -0.473073 -0.234323

C 1.086626 0.555840 1.413111

C 2.956959 -0.220266 2.362738

H 3.909443 -0.736050 2.419420

C 2.289199 0.469196 3.320254

H 2.552700 0.644153 4.356043

C 0.194977 1.898580 3.359154

C -1.182585 1.243295 3.490982

H -1.905673 1.962964 3.913611

H -1.549325 0.848149 2.530144

H -1.133443 0.392367 4.182585

C 0.687273 2.285191 4.756947

H -0.020438 3.003854 5.194693

H 0.731935 1.416435 5.429450

H 1.675887 2.765484 4.726289

C 0.144412 3.164428 2.494211

H -0.562928 3.897345 2.918638

H 1.133827 3.642417 2.451257

H -0.121589 2.926678 1.455511

C 0.511204 -1.065266 -1.323913

C 2.241504 -2.357837 -1.915481

H 3.260965 -2.727349 -1.913091

C 1.183667 -2.741284 -2.669216

H 1.113149 -3.515187 -3.423987

C -1.226070 -2.084343 -2.902635

C -1.975247 -3.174473 -2.129317

H -2.984300 -3.316430 -2.544219

H -1.437588 -4.132995 -2.190609

H -2.080432 -2.902767 -1.070545

C -1.976412 -0.757791 -2.838513

H -1.405595 0.049909 -3.319036

H -2.945564 -0.862559 -3.347979

H -2.169925 -0.475124 -1.800315

C -1.086523 -2.482681 -4.378780

H -0.449187 -1.769954 -4.923060

H -0.675626 -3.493757 -4.508380

H -2.082148 -2.479219 -4.844407

C 4.193533 -1.327744 -0.032763

C 5.443586 -0.871966 -0.469461

H 5.513696 0.097572 -0.969640

C 6.606896 -1.625014 -0.285636

H 7.565849 -1.238760 -0.640612

C 6.546743 -2.864366 0.345907

H 7.454262 -3.455486 0.491604

C 5.312973 -3.346573 0.788301

H 5.250204 -4.320048 1.281571

C 4.161783 -2.586715 0.596552

H 3.202188 -2.984826 0.942334

C 3.064256 0.963276 -0.970050

C 2.782930 1.181725 -2.325502

H 2.341220 0.376111 -2.919002

C 3.038119 2.407377 -2.944138

H 2.794032 2.544253 -4.000547

C 3.597837 3.456080 -2.217879

H 3.801199 4.415679 -2.699585

C 3.892210 3.266838 -0.866499

H 4.332965 4.080095 -0.283431

C 3.622393 2.040837 -0.259781

H 3.858467 1.916317 0.802421

C -2.973356 -1.197522 0.728097

C -4.273073 -1.435679 0.171388

C -4.892048 -2.672910 0.342722

H -5.876648 -2.846914 -0.095719

C -4.286761 -3.699698 1.060128

H -4.783546 -4.664231 1.180259

C -3.037774 -3.465736 1.621899

H -2.551994 -4.264818 2.189371

C -2.370505 -2.248603 1.475774

C -4.999656 -0.315332 -0.562713

H -4.261787 0.235572 -1.171772

C -5.610208 0.672971 0.445294

H -5.935781 1.610140 -0.038302

H -6.488511 0.222924 0.933078

H -4.898251 0.899167 1.255950

C -6.059271 -0.778782 -1.562176

H -6.919685 -1.251172 -1.063627

H -6.445303 0.079359 -2.133683

H -5.640393 -1.504025 -2.275388

C -0.996526 -2.101997 2.107757

H -0.653411 -1.078714 1.917146

C -1.040511 -2.291037 3.628348

H -1.248819 -3.339136 3.897350

H -0.072781 -2.019489 4.081284

H -1.828802 -1.676009 4.088158

C 0.037172 -3.044519 1.481982

H -0.198985 -4.098170 1.702660

H 0.076077 -2.927149 0.390896

H 1.042206 -2.829336 1.878347

H -2.874693 0.495548 -0.191986

C -0.763184 2.328011 -1.104796

C -1.981910 2.661306 -1.728352

C 0.163527 3.392008 -1.097311

H -2.763927 1.891853 -1.844952

C -0.157835 4.641735 -1.625622

H 1.159071 3.241735 -0.665956

H 0.568533 5.458275 -1.626603

C -1.438169 4.836031 -2.146552

H -1.741734 5.814913 -2.535828

N -2.346306 3.862323 -2.202087

**2-K^γ^**

Atom X Y Z

Fe -0.380673 0.463196 -0.138750

K -2.705486 2.770458 1.080261

N 2.222879 -0.192021 1.201723

N 1.155725 0.877968 2.753874

N 1.809523 -1.337561 -1.112983

N 0.108660 -1.927360 -2.318396

N -2.316276 0.025123 0.508803

B 2.809907 -0.476230 -0.250405

C 1.097366 0.519245 1.434734

C 2.965209 -0.294976 2.355325

H 3.914992 -0.817104 2.394941

C 2.304534 0.370839 3.334096

H 2.572833 0.515520 4.373286

C 0.225548 1.819290 3.418946

C -1.169091 1.194035 3.509341

H -1.882304 1.919772 3.937979

H -1.529108 0.829948 2.534022

H -1.152475 0.325095 4.179472

C 0.708076 2.136477 4.837433

H 0.005710 2.845476 5.298860

H 0.732676 1.237936 5.470718

H 1.703370 2.603624 4.839295

C 0.218499 3.119904 2.605623

H -0.479483 3.852006 3.046236

H 1.219165 3.575816 2.598684

H -0.032830 2.931928 1.553283

C 0.498244 -1.067891 -1.331729

C 2.229528 -2.348152 -1.946409

H 3.250857 -2.712073 -1.956155

C 1.167414 -2.732030 -2.693332

H 1.094765 -3.500780 -3.453030

C -1.247931 -2.088711 -2.900753

C -1.992320 -3.173093 -2.115125

H -3.001201 -3.322888 -2.527683

H -1.451152 -4.130152 -2.167103

H -2.098094 -2.891096 -1.059284

C -1.995515 -0.761159 -2.839424

H -1.429721 0.038787 -3.338553

H -2.973219 -0.869752 -3.331737

H -2.169913 -0.467174 -1.800892

C -1.120861 -2.497600 -4.375104

H -0.484758 -1.791641 -4.929534

H -0.715707 -3.511497 -4.500621

H -2.120117 -2.493161 -4.832907

C 4.196002 -1.321152 -0.080010

C 5.436142 -0.844829 -0.522736

H 5.490542 0.134394 -1.005675

C 6.608851 -1.589443 -0.366220

H 7.559677 -1.187123 -0.725154

C 6.568497 -2.840608 0.243164

H 7.483503 -3.425021 0.367403

C 5.344927 -3.343294 0.691113

H 5.297681 -4.326105 1.167297

C 4.184179 -2.591662 0.526623

H 3.232679 -3.005755 0.876579

C 3.031541 0.976451 -0.959996

C 2.732108 1.218703 -2.307366

H 2.293222 0.419940 -2.912192

C 2.964803 2.459870 -2.903292

H 2.705440 2.616175 -3.953300

C 3.520200 3.500037 -2.162084

H 3.703873 4.472346 -2.625646

C 3.834210 3.286556 -0.818797

H 4.272378 4.093159 -0.224642

C 3.586265 2.045496 -0.234292

H 3.837173 1.901684 0.822151

C -2.987148 -1.173175 0.725290

C -4.293083 -1.386214 0.172926

C -4.933307 -2.612965 0.341774

H -5.922621 -2.768070 -0.093057

C -4.343524 -3.653037 1.052830

H -4.856968 -4.608972 1.171434

C -3.088217 -3.443631 1.610085

H -2.614231 -4.253786 2.171741

C -2.399475 -2.238239 1.465930

C -5.001523 -0.250132 -0.554910

H -4.255791 0.282913 -1.170687

C -5.579774 0.752729 0.457609

H -5.896317 1.693565 -0.025047

H -6.458704 0.320149 0.959779

H -4.851660 0.970953 1.255761

C -6.080980 -0.690547 -1.543469

H -6.946539 -1.143392 -1.035875

H -6.454541 0.175490 -2.111582

H -5.685836 -1.425280 -2.260499

C -1.018447 -2.122034 2.089574

H -0.662737 -1.100475 1.914372

C -1.051362 -2.338591 3.606854

H -1.262939 -3.390261 3.858669

H -0.078284 -2.080867 4.056311

H -1.832390 -1.728300 4.084941

C -0.002389 -3.067534 1.439048

H -0.250702 -4.121319 1.645344

H 0.028274 -2.933960 0.349736

H 1.009305 -2.871619 1.828739

H -2.863008 0.523640 -0.188623

C -0.766693 2.320039 -1.085109

C -1.940383 2.620850 -1.815536

C 0.118615 3.414059 -0.998768

C -2.177639 3.894287 -2.340207

H -2.702597 1.851441 -2.000221

C -0.195496 4.657283 -1.551817

H 1.085394 3.301981 -0.497238

N -1.333603 4.916827 -2.199222

H -3.099229 4.098687 -2.901248

H 0.511893 5.490990 -1.461872

**3-K**

Atom X Y Z

Fe -0.440517 0.681872 -0.017545

N 1.989429 -0.428120 -1.466983

N 0.654259 0.219318 -3.047265

N 1.925679 -0.871154 1.122163

N 0.517076 -0.833301 2.765889

N -2.282799 -0.119603 -0.524869

H -2.777854 0.601123 -1.045091

C 0.734041 -0.349502 1.501148

C 2.440516 -1.660866 2.125783

H 3.394678 -2.168423 2.039032

C 1.564379 -1.644901 3.157507

H 1.619587 -2.143124 4.117178

C -0.656713 -0.530081 3.615927

C -1.899963 -1.155883 2.980924

H -2.809035 -0.882295 3.543153

H -1.839189 -2.251768 2.983990

H -1.990347 -0.860124 1.928954

C -0.472778 -1.115046 5.019031

H -1.346785 -0.845540 5.629326

H 0.420498 -0.710909 5.516989

H -0.409221 -2.212555 5.003787

C -0.788829 0.990305 3.734401

H -1.665398 1.252249 4.351712

H -0.861875 1.467842 2.746912

H 0.100107 1.413675 4.223743

C 0.815014 0.208276 -1.688896

C 2.549266 -0.818850 -2.662471

H 3.502784 -1.331661 -2.722652

C 1.714543 -0.427776 -3.654191

H 1.807666 -0.555953 -4.725447

C -0.478538 0.797912 -3.816083

C -0.010716 1.145695 -5.236060

H -0.814171 1.693376 -5.748591

H 0.204337 0.251852 -5.838637

H 0.883140 1.786707 -5.219183

C -0.945688 2.090454 -3.150568

H -1.788682 2.512254 -3.717773

H -0.133815 2.830058 -3.123776

H -1.269786 1.928240 -2.117529

C -1.617721 -0.221916 -3.890114

H -2.444719 0.177414 -4.497128

H -1.987322 -0.446266 -2.880714

H -1.272846 -1.157687 -4.354597

C 4.017720 -1.519132 -0.284449

C 5.373543 -1.173555 -0.237773

H 5.652809 -0.129533 -0.072113

C 6.381630 -2.128953 -0.399422

H 7.430642 -1.824468 -0.357860

C 6.052962 -3.464770 -0.614065

H 6.838437 -4.213959 -0.740563

C 4.707944 -3.837953 -0.668141

H 4.435649 -4.882799 -0.838526

C 3.714785 -2.875762 -0.506471

H 2.665244 -3.185408 -0.554837

C 3.353998 1.080865 0.176666

C 3.832272 1.430189 1.451068

H 3.812858 0.685850 2.253450

C 4.324598 2.702762 1.733256

H 4.684071 2.938631 2.738344

C 4.350544 3.678470 0.736313

H 4.728338 4.680737 0.952853

C 3.887270 3.359304 -0.537720

H 3.898839 4.113979 -1.328489

C 3.402479 2.077802 -0.807573

H 3.044090 1.855973 -1.817216

C -3.114171 -1.201238 -0.327273

C -2.567308 -2.487451 -0.008903

C -3.421912 -3.541033 0.298832

H -2.992893 -4.515319 0.543941

C -4.809699 -3.400174 0.287607

H -5.457645 -4.244119 0.531727

C -5.350258 -2.172493 -0.068748

H -6.437074 -2.068454 -0.114380

C -4.540824 -1.074197 -0.379121

C -1.064515 -2.707259 -0.082519

H -0.587630 -1.780181 0.256945

C -0.610221 -2.927309 -1.529291

H 0.488715 -2.971756 -1.591433

H -0.942699 -2.105470 -2.171984

H -1.022696 -3.868198 -1.928572

C -0.524163 -3.824478 0.810073

H 0.575871 -3.783047 0.827341

H -0.810325 -4.823925 0.443977

H -0.873133 -3.736064 1.849618

C -5.189339 0.215277 -0.873722

H -4.522056 1.065904 -0.637462

C -5.312347 0.206268 -2.403868

H -5.720018 1.157218 -2.783399

H -5.980027 -0.608936 -2.722832

H -4.334257 0.035588 -2.875358

C -6.536522 0.538969 -0.220911

H -6.877174 1.544171 -0.514340

H -6.482350 0.497141 0.879878

H -7.320989 -0.167628 -0.530095

B 2.812050 -0.428538 -0.119531

N -1.172249 2.514931 0.508455

C -0.402170 3.746470 0.723072

H -1.843575 2.749781 -0.233238

K -3.331668 1.367204 1.698134

C -1.318317 4.838528 1.305055

H -2.155233 5.050572 0.617126

H -1.741268 4.506783 2.268814

H -0.782167 5.785747 1.480251

C 0.744079 3.484931 1.703716

H 1.364666 2.640402 1.369403

H 1.400424 4.364525 1.784378

H 0.366433 3.258164 2.710757

C 0.201964 4.258554 -0.595651

H 0.784795 5.182646 -0.450527

H 0.872235 3.495614 -1.019034

H -0.592533 4.471272 -1.330904

**^1^TS^α^_1-3_**

Imaginary frequency: -1346.51 cm^-1^

Atom X Y Z

Fe -0.636782 0.181198 0.055327

N 1.855865 -0.810014 -1.205709

N 0.299609 -0.675198 -2.711778

N 2.031072 -0.324839 1.298524

N 0.662832 0.376671 2.827990

N -2.499866 0.041186 0.024294

C 0.797167 0.219926 1.461916

C 2.647442 -0.519489 2.512388

H 3.641337 -0.947508 2.598980

C 1.799989 -0.082289 3.472612

H 1.926307 -0.078430 4.547405

C -0.453738 1.059043 3.540850

C -1.778333 0.334357 3.296224

H -2.590600 0.864840 3.816370

H -1.733708 -0.692446 3.687268

H -2.026534 0.293381 2.224402

C -0.181530 1.063559 5.050966

H -1.027966 1.557038 5.549137

H 0.729605 1.623634 5.311124

H -0.106741 0.044388 5.459405

C -0.523458 2.512707 3.061573

H -1.276134 3.065906 3.643839

H -0.806188 2.562351 2.006726

H 0.453049 3.005971 3.185471

C 0.600275 -0.327305 -1.408868

C 2.312282 -1.475130 -2.318609

H 3.293248 -1.938291 -2.358727

C 1.344348 -1.400327 -3.260665

H 1.329457 -1.803462 -4.265068

C -0.944497 -0.387679 -3.481655

C -2.020526 -1.423362 -3.141901

H -2.932598 -1.220828 -3.723979

H -2.281613 -1.363721 -2.077937

H -1.678181 -2.442441 -3.380829

C -0.633790 -0.448206 -4.985091

H -1.526571 -0.122033 -5.537133

H -0.396703 -1.465150 -5.331238

H 0.197379 0.222284 -5.252417

C -1.455849 1.014158 -3.156741

H -2.269228 1.275053 -3.850581

H -0.654975 1.763150 -3.247828

H -1.852066 1.037848 -2.138851

C 4.039086 -1.423618 0.065549

C 5.380395 -1.081807 -0.148946

H 5.629721 -0.047477 -0.403257

C 6.406817 -2.025729 -0.047297

H 7.443617 -1.724194 -0.221711

C 6.111533 -3.348151 0.275810

H 6.910837 -4.089930 0.356423

C 4.782427 -3.716747 0.496645

H 4.536091 -4.751239 0.751593

C 3.770530 -2.765208 0.391604

H 2.733654 -3.067823 0.569591

C 3.319575 1.170786 -0.385407

C 3.606187 2.092324 0.634455

H 3.502372 1.783680 1.679021

C 3.984778 3.406896 0.360281

H 4.181815 4.099564 1.183401

C 4.095488 3.843270 -0.959704

H 4.381044 4.875330 -1.180591

C 3.830203 2.945986 -1.993863

H 3.904786 3.273736 -3.034574

C 3.452191 1.634504 -1.704128

H 3.224199 0.958455 -2.533748

C 0.133744 2.966122 -0.485077

H 1.133156 2.528136 -0.519844

C -0.068760 4.331501 -0.644805

H 0.788012 4.985930 -0.814469

C -1.374979 4.817606 -0.577790

H -1.571961 5.888585 -0.691567

C -2.418213 3.917138 -0.368843

H -3.450420 4.278081 -0.322315

C -3.255628 -1.070696 0.192506

C -2.696532 -2.345626 0.557399

C -3.514026 -3.465636 0.703714

H -3.059459 -4.424703 0.976549

C -4.890931 -3.401880 0.505861

H -5.515021 -4.291895 0.619032

C -5.455742 -2.172290 0.156097

H -6.537205 -2.117017 -0.000550

C -4.683082 -1.026434 -0.000252

C -1.198029 -2.493689 0.772644

H -0.761745 -1.477657 0.874398

C -0.488684 -3.238977 -0.359841

H 0.603297 -3.223894 -0.220673

H -0.703053 -2.791522 -1.335732

H -0.824178 -4.288907 -0.385328

C -0.833772 -3.131491 2.119523

H 0.250203 -3.049122 2.303331

H -1.099157 -4.200994 2.137448

H -1.363736 -2.641471 2.947786

C -5.299212 0.316008 -0.352404

H -4.587828 0.809399 -1.036258

C -6.647334 0.228485 -1.067793

H -6.968372 1.228128 -1.403552

H -7.440980 -0.160729 -0.407836

H -6.593834 -0.428020 -1.950464

C -5.405804 1.205767 0.893901

H -5.759394 2.218525 0.635290

H -4.425371 1.302536 1.381099

H -6.112969 0.767242 1.618436

B 2.808812 -0.353191 -0.055164

C -2.167884 2.547411 -0.217003

N -0.879864 2.121407 -0.268845

H -2.778721 1.353322 -0.085504

**^3^TS^α^_1-3_**

Imaginary frequency: -1411.35 cm^-1^

Atom X Y Z

Fe 0.481216 -0.341010 0.177838

N -1.817594 0.901626 -1.244854

N -0.124261 0.970992 -2.597590

N -2.227691 0.331229 1.231243

N -0.995163 -0.278319 2.907350

N 2.315614 0.017691 0.377010

C -1.004237 -0.160759 1.540254

C -2.969103 0.529893 2.372406

H -3.982836 0.917032 2.348943

C -2.203412 0.155376 3.426194

H -2.430004 0.176471 4.484697

C 0.092830 -0.886676 3.721721

C 1.354967 -0.025856 3.644065

H 2.141378 -0.464095 4.277771

H 1.144316 0.991432 4.003736

H 1.734925 0.028438 2.611540

C -0.351463 -0.984976 5.185503

H 0.457184 -1.458957 5.759612

H -1.254015 -1.603790 5.304823

H -0.533484 0.005677 5.629169

C 0.362566 -2.298750 3.194777

H 1.132889 -2.788895 3.809068

H 0.719949 -2.266243 2.160615

H -0.555575 -2.905948 3.223722

C -0.517743 0.525503 -1.360622

C -2.218319 1.597668 -2.360520

H -3.224417 1.988005 -2.474539

C -1.162423 1.654572 -3.206109

H -1.080641 2.123223 -4.178709

C 1.202305 0.797596 -3.252871

C 2.129072 1.936612 -2.822014

H 3.111880 1.833151 -3.306288

H 2.290246 1.919317 -1.737909

H 1.702657 2.913315 -3.100463

C 1.016719 0.833693 -4.777829

H 1.977772 0.587428 -5.251065

H 0.721554 1.826359 -5.148881

H 0.271779 0.094059 -5.109225

C 1.800139 -0.558668 -2.880736

H 2.712190 -0.726845 -3.473158

H 1.089415 -1.374432 -3.081180

H 2.074339 -0.580223 -1.819982

C -4.192899 1.207389 -0.223679

C -5.457761 0.693284 -0.535507

H -5.551971 -0.366488 -0.789392

C -6.601244 1.497755 -0.531145

H -7.573671 1.063392 -0.779949

C -6.504278 2.849956 -0.211283

H -7.395874 3.482890 -0.206917

C -5.254837 3.389583 0.103351

H -5.163983 4.449810 0.355390

C -4.124048 2.576102 0.095138

H -3.150475 3.012148 0.341427

C -3.108410 -1.264734 -0.613421

C -3.457013 -2.220851 0.354200

H -3.529548 -1.917130 1.403200

C -3.693377 -3.556036 0.024882

H -3.950846 -4.273238 0.809271

C -3.586647 -3.979802 -1.299794

H -3.761070 -5.026549 -1.562697

C -3.242725 -3.051784 -2.282307

H -3.142753 -3.370076 -3.323678

C -3.010341 -1.719453 -1.937851

H -2.725563 -1.015046 -2.725366

C 0.143629 -3.414274 -0.492406

H -0.923405 -3.181882 -0.570155

C 0.622823 -4.698250 -0.727530

H -0.067723 -5.498837 -1.000032

C 1.996371 -4.917008 -0.602985

H 2.412171 -5.914622 -0.779554

C 2.824427 -3.851826 -0.255145

H 3.903056 -4.009568 -0.155660

C 3.143323 1.091321 0.370669

C 2.718670 2.416474 0.756036

C 3.595553 3.497171 0.659262

H 3.236954 4.493479 0.941118

C 4.904085 3.356091 0.207364

H 5.569673 4.219826 0.132336

C 5.344453 2.079688 -0.146896

H 6.372942 1.958490 -0.498809

C 4.513465 0.966450 -0.070270

C 1.302637 2.676768 1.245233

H 0.841826 1.691810 1.406862

C 0.441467 3.410387 0.211030

H -0.600485 3.500810 0.561227

H 0.421569 2.873740 -0.745777

H 0.830768 4.425727 0.022882

C 1.266545 3.422664 2.583605

H 0.244385 3.428262 2.998315

H 1.582828 4.473778 2.473276

H 1.937128 2.953662 3.318923

C 5.029185 -0.416398 -0.432962

H 4.204362 -0.942754 -0.937506

C 6.217891 -0.424670 -1.394810

H 6.442898 -1.456236 -1.711443

H 7.133848 -0.021956 -0.930647

H 6.009285 0.171510 -2.297216

C 5.356241 -1.214860 0.835725

H 5.652471 -2.250063 0.594528

H 4.480668 -1.257855 1.498903

H 6.185300 -0.737385 1.385119

B -2.833568 0.300281 -0.212166

C 2.296636 -2.569988 -0.033226

N 0.955195 -2.405929 -0.163122

H 2.672942 -1.268562 0.229528

**^5^TS^α^_1-3_**

Imaginary frequency: -1424.07 cm^-1^

Atom X Y Z

Fe 0.509121 0.390137 0.155981

N -2.270672 -0.065162 1.260216

N -1.149163 0.846181 2.878025

N -1.771530 -1.128919 -1.083931

N -0.059947 -1.514907 -2.358742

N 2.351690 0.128986 0.633518

C -0.494119 -0.739211 -1.329255

C -2.120783 -2.150053 -1.940011

H -3.102235 -2.612277 -1.931155

C -1.051451 -2.402763 -2.734898

H -0.927638 -3.134712 -3.525079

C 1.275940 -1.476899 -3.010191

C 2.125307 -2.631523 -2.469957

H 3.110342 -2.639568 -2.959812

H 1.636235 -3.601264 -2.654742

H 2.295087 -2.522670 -1.389801

C 1.077718 -1.620509 -4.526954

H 2.047787 -1.493521 -5.028755

H 0.388213 -0.850730 -4.905981

H 0.690833 -2.609629 -4.813656

C 1.960967 -0.140163 -2.736140

H 2.906225 -0.104632 -3.297828

H 2.202691 -0.019775 -1.671481

H 1.329972 0.704817 -3.048826

C -1.070705 0.487999 1.566534

C -3.088726 -0.050143 2.369502

H -4.104107 -0.432394 2.351910

C -2.392443 0.514190 3.387600

H -2.690755 0.692019 4.413562

C -0.080767 1.567313 3.621498

C 1.143585 0.662130 3.774394

H 1.904781 1.170561 4.386400

H 1.595042 0.430838 2.794179

H 0.864780 -0.276481 4.275843

C -0.599105 1.959959 5.008591

H 0.190114 2.521337 5.528274

H -0.836726 1.078803 5.623976

H -1.488097 2.606795 4.949857

C 0.280358 2.836997 2.844449

H 1.045304 3.406804 3.393052

H -0.606659 3.474489 2.705146

H 0.684308 2.586770 1.857208

C -4.185247 -1.176157 -0.106475

C -5.410223 -0.723122 -0.611794

H -5.454840 0.255538 -1.097941

C -6.576676 -1.487235 -0.511200

H -7.516247 -1.101899 -0.917112

C -6.544731 -2.736191 0.104307

H -7.454632 -3.337045 0.184920

C -5.336633 -3.213190 0.618193

H -5.296257 -4.192010 1.104026

C -4.182357 -2.440795 0.511225

H -3.242577 -2.828203 0.918001

C -3.020513 1.147346 -0.917328

C -2.933569 1.306996 -2.309683

H -2.683542 0.441592 -2.931213

C -3.137520 2.541260 -2.927397

H -3.049441 2.627913 -4.013877

C -3.439548 3.666513 -2.160186

H -3.590956 4.638216 -2.637672

C -3.534490 3.537126 -0.774938

H -3.759351 4.411685 -0.158316

C -3.328115 2.295917 -0.171288

H -3.394225 2.224231 0.918572

C 0.258780 3.253140 -1.171320

H -0.787269 2.975030 -1.335539

C 0.749583 4.487465 -1.580709

H 0.089385 5.200917 -2.077782

C 2.095028 4.771099 -1.335542

H 2.519156 5.732308 -1.644361

C 2.883552 3.816849 -0.697173

H 3.939711 4.025170 -0.499225

C 3.120017 -0.977102 0.620376

C 2.665830 -2.258425 1.121157

C 3.469804 -3.391533 1.017956

H 3.082391 -4.346325 1.393134

C 4.739323 -3.357934 0.445505

H 5.348140 -4.261993 0.366225

C 5.212343 -2.128574 -0.019171

H 6.210538 -2.087549 -0.465040

C 4.457130 -0.963051 0.060413

C 1.292532 -2.400304 1.758347

H 0.852821 -1.393071 1.785755

C 1.376170 -2.887448 3.208729

H 0.384733 -2.861763 3.693133

H 2.064926 -2.259766 3.793681

H 1.746958 -3.925071 3.263457

C 0.341337 -3.287928 0.947560

H -0.673947 -3.276599 1.379021

H 0.691227 -4.334154 0.929398

H 0.264183 -2.945409 -0.093294

C 5.011377 0.364947 -0.426494

H 4.188506 0.884825 -0.942557

C 5.413253 1.252091 0.759417

H 5.724733 2.256216 0.423548

H 6.253239 0.796484 1.310742

H 4.567301 1.369819 1.451551

C 6.161816 0.252970 -1.426748

H 6.418364 1.248616 -1.824336

H 5.894864 -0.395231 -2.276587

H 7.075979 -0.157785 -0.965890

B -2.801653 -0.311458 -0.208654

N 1.033362 2.349364 -0.564104

C 2.346488 2.580858 -0.301708

H 2.702483 1.367197 0.251027

**^1^TS^γ^_1-3_**

Imaginary frequency: -1507.74 cm^-1^

Atom X Y Z

Fe 0.383972 0.406785 -0.267067

N -2.061498 0.180668 1.271241

N -0.637395 1.250505 2.511596

N -1.905276 -1.266921 -0.810409

N -0.303676 -1.972135 -2.092227

N 2.013509 -0.232990 0.146354

C -0.610365 -1.031188 -1.139464

C -2.391894 -2.348766 -1.499554

H -3.409929 -2.705738 -1.381441

C -1.394620 -2.804692 -2.292835

H -1.380847 -3.647768 -2.971843

C 0.954244 -2.144809 -2.870189

C 1.868961 -3.128133 -2.136023

H 2.801136 -3.277061 -2.702167

H 1.373737 -4.105267 -2.021551

H 2.131072 -2.754010 -1.139755

C 0.609085 -2.705906 -4.260254

H 1.520546 -2.702699 -4.874819

H -0.147859 -2.086132 -4.764447

H 0.250070 -3.744694 -4.225670

C 1.642759 -0.796927 -3.079949

H 2.520236 -0.935198 -3.728857

H 1.988587 -0.378697 -2.128057

H 0.960780 -0.081189 -3.564086

C -0.802882 0.679714 1.274966

C -2.673164 0.408207 2.479808

H -3.688103 0.085759 2.689611

C -1.789204 1.073217 3.262179

H -1.893312 1.412211 4.284828

C 0.455351 2.188757 2.896283

C 1.812628 1.486895 2.864647

H 2.610233 2.221309 3.052795

H 1.997016 1.006512 1.895514

H 1.864874 0.710205 3.642313

C 0.218104 2.709393 4.318483

H 1.053814 3.372812 4.582405

H 0.197761 1.891447 5.054861

H -0.710004 3.294518 4.403171

C 0.401639 3.374867 1.925545

H 1.187065 4.106528 2.163934

H -0.578769 3.872417 1.988082

H 0.554293 3.051392 0.888946

C -4.252501 -0.795046 0.240028

C -5.427707 -0.209032 -0.247577

H -5.354118 0.690014 -0.866146

C -6.688206 -0.741867 0.035496

H -7.585306 -0.258608 -0.361513

C -6.804275 -1.886099 0.821788

H -7.788623 -2.306137 1.045442

C -5.649503 -2.491446 1.321856

H -5.725694 -3.389963 1.940414

C -4.399094 -1.948976 1.031651

H -3.502425 -2.434620 1.430605

C -2.781058 1.150321 -1.012421

C -2.726647 1.064412 -2.413470

H -2.697373 0.077655 -2.887085

C -2.685135 2.200135 -3.222777

H -2.630348 2.093573 -4.309752

C -2.697288 3.469953 -2.646536

H -2.647449 4.363521 -3.274071

C -2.756922 3.586611 -1.258565

H -2.749502 4.575383 -0.792720

C -2.802183 2.442275 -0.462299

H -2.833165 2.559324 0.625594

C 1.071071 3.959643 -1.872456

H 0.220206 4.202335 -2.522163

C 2.745038 4.681331 -0.530312

H 3.276080 5.532572 -0.080852

C 3.147525 3.377158 -0.246119

H 3.990443 3.240202 0.441510

C 2.693076 -1.301832 0.649612

C 2.081654 -2.310135 1.469862

C 2.835707 -3.388482 1.932988

H 2.340796 -4.142400 2.555052

C 4.183193 -3.543018 1.623964

H 4.751680 -4.399035 1.995837

C 4.787938 -2.576306 0.821725

H 5.845892 -2.686331 0.567205

C 4.085466 -1.476644 0.336693

C 0.606330 -2.252170 1.820940

H 0.218461 -1.352785 1.336857

C 0.352925 -2.103875 3.322785

H -0.721809 -1.953664 3.521226

H 0.892309 -1.235357 3.727418

H 0.681877 -2.995185 3.884830

C -0.177431 -3.433990 1.244586

H -1.258640 -3.303653 1.416175

H 0.125729 -4.391066 1.703064

H -0.023305 -3.517295 0.159015

C 4.792736 -0.419356 -0.492512

H 4.039661 0.044005 -1.147431

C 5.325554 0.690270 0.420695

H 5.738906 1.529079 -0.162877

H 6.116144 0.297216 1.082353

H 4.517352 1.083422 1.054094

C 5.899099 -0.961383 -1.399366

H 6.268053 -0.165185 -2.065840

H 5.531599 -1.789592 -2.025538

H 6.766459 -1.331739 -0.828002

B -2.772372 -0.192875 -0.071110

C 2.484381 2.263492 -0.795232

H 2.499341 0.853378 -0.297534

C 1.424089 2.625618 -1.650853

H 0.827257 1.858446 -2.169964

N 1.713596 4.989533 -1.321777

**^3^TS^γ^_1-3_**

Imaginary frequency: -1378.67 cm^-1^

Atom X Y Z

Fe 0.472323 0.184624 0.019551

N -2.217890 0.024021 1.252749

N -0.962102 1.212201 2.565231

N -1.884467 -1.339057 -0.903880

N -0.213515 -1.916947 -2.162270

N 2.290162 0.002671 0.230892

C -0.579829 -1.065843 -1.149659

C -2.323895 -2.351549 -1.721196

H -3.343895 -2.720744 -1.693228

C -1.283369 -2.724396 -2.503805

H -1.234220 -3.491261 -3.266031

C 1.119855 -2.032520 -2.819918

C 2.040447 -2.871153 -1.931907

H 3.032847 -2.973563 -2.395926

H 1.619722 -3.876862 -1.776370

H 2.179105 -2.392122 -0.957435

C 0.957757 -2.724397 -4.180894

H 1.929945 -2.708742 -4.693142

H 0.228768 -2.200868 -4.818088

H 0.659945 -3.779296 -4.088116

C 1.704968 -0.641502 -3.065558

H 2.640704 -0.735428 -3.636452

H 1.942813 -0.140096 -2.121484

H 1.004582 -0.015245 -3.638099

C -0.988322 0.579449 1.350080

C -2.948912 0.283415 2.387778

H -3.967101 -0.069528 2.517139

C -2.169687 1.023484 3.214212

H -2.386921 1.411729 4.201093

C 0.090037 2.163666 3.028699

C 1.433763 1.450142 3.181472

H 2.186906 2.164881 3.545809

H 1.787251 1.041698 2.223715

H 1.352377 0.628897 3.908447

C -0.307466 2.747576 4.388945

H 0.485851 3.436712 4.710567

H -0.403140 1.967843 5.160256

H -1.244404 3.322370 4.338460

C 0.178135 3.301556 2.007267

H 0.921233 4.046200 2.328144

H -0.797178 3.799119 1.895122

H 0.484867 2.928493 1.023541

C -4.241094 -1.220050 0.197366

C -5.497800 -0.798880 -0.255341

H -5.567440 0.118154 -0.847366

C -6.661527 -1.518824 0.030161

H -7.627051 -1.161737 -0.338857

C -6.593631 -2.688778 0.783264

H -7.501448 -3.254817 1.008899

C -5.353127 -3.131824 1.247781

H -5.285959 -4.048728 1.839926

C -4.201384 -2.404350 0.955704

H -3.235148 -2.763463 1.324905

C -3.097685 0.952002 -1.003264

C -3.054729 0.928497 -2.406085

H -2.845039 -0.014493 -2.920865

C -3.253483 2.078035 -3.171735

H -3.199948 2.021829 -4.262509

C -3.505622 3.299312 -2.548400

H -3.647896 4.205886 -3.142006

C -3.552893 3.352744 -1.156056

H -3.730188 4.306374 -0.651913

C -3.352076 2.195673 -0.403818

H -3.379538 2.269980 0.687709

C 0.304337 3.730009 -1.685203

H -0.702410 3.756460 -2.122074

C 2.056644 4.851502 -0.790444

H 2.492900 5.819133 -0.502851

C 2.764415 3.671361 -0.564433

H 3.748178 3.746780 -0.084548

C 3.183619 -0.952229 0.629140

C 2.831687 -1.976653 1.571946

C 3.750742 -2.976207 1.895410

H 3.457570 -3.756674 2.605029

C 5.021874 -3.018887 1.332248

H 5.722834 -3.815601 1.593852

C 5.382775 -2.022230 0.426014

H 6.381824 -2.048560 -0.017576

C 4.505393 -1.000547 0.071690

C 1.447882 -2.019583 2.199933

H 0.988564 -1.040050 2.008683

C 1.482999 -2.207088 3.719542

H 0.480204 -2.049024 4.149697

H 2.178155 -1.496732 4.192164

H 1.804249 -3.223480 4.002419

C 0.542295 -3.073676 1.551708

H -0.477991 -3.021277 1.966510

H 0.935312 -4.089857 1.725155

H 0.463859 -2.922913 0.466764

C 4.936992 0.098302 -0.883970

H 4.042330 0.423444 -1.435129

C 5.427265 1.317064 -0.093951

H 5.647447 2.164338 -0.763706

H 6.341037 1.068807 0.472497

H 4.657543 1.643320 0.620088

C 5.973502 -0.334363 -1.921711

H 6.128942 0.469492 -2.658931

H 5.646956 -1.236215 -2.463540

H 6.955506 -0.551680 -1.469167

B -2.863324 -0.401461 -0.112795

C 2.231848 2.415988 -0.912863

H 2.558040 1.091758 -0.354899

C 0.957975 2.508276 -1.506304

H 0.417870 1.606716 -1.840794

N 0.836789 4.901475 -1.334569

**^5^TS^γ^_1-3_**

Imaginary frequency: -484.88 cm^-1^

Atom X Y Z

Fe -0.420664 0.153337 0.181875

N 2.262800 1.012008 -0.868727

N 0.848728 2.492150 -1.592262

N 2.202568 -1.253851 0.463982

N 0.741988 -2.557515 1.399329

N -2.208659 -0.173619 -0.290787

C 0.920066 -1.303606 0.906922

C 2.806085 -2.475808 0.656722

H 3.838844 -2.664953 0.383380

C 1.895894 -3.299432 1.233426

H 1.985329 -4.337091 1.534112

C -0.484807 -3.109876 2.032038

C -1.207260 -3.995608 1.012883

H -2.126289 -4.410907 1.452146

H -0.564715 -4.832136 0.695844

H -1.494328 -3.417867 0.124183

C -0.065370 -3.936095 3.257685

H -0.967343 -4.272741 3.788532

H 0.539007 -3.330737 3.950171

H 0.506642 -4.835656 2.987560

C -1.391116 -1.975715 2.509366

H -2.233527 -2.407331 3.068568

H -1.810893 -1.396781 1.674759

H -0.845548 -1.292345 3.177820

C 0.979706 1.430953 -0.754987

C 2.927054 1.808481 -1.774296

H 3.975325 1.666416 -2.015815

C 2.046374 2.735840 -2.232174

H 2.190279 3.532883 -2.951488

C -0.392685 3.307504 -1.728573

C -1.478816 2.467252 -2.404362

H -2.386204 3.074308 -2.535885

H -1.756344 1.594648 -1.793813

H -1.134743 2.119916 -3.391388

C -0.100606 4.542210 -2.585381

H -1.014371 5.150444 -2.633859

H 0.175040 4.274978 -3.617209

H 0.694996 5.166511 -2.150461

C -0.818786 3.760274 -0.330286

H -1.733747 4.367578 -0.383264

H -0.018013 4.348007 0.144687

H -1.049767 2.901167 0.309898

C 4.436360 -0.372905 -0.547584

C 5.684869 -0.059344 0.004283

H 5.725242 0.495773 0.945605

C 6.879287 -0.436093 -0.616780

H 7.836896 -0.174314 -0.158279

C 6.851997 -1.142755 -1.816671

H 7.783853 -1.439310 -2.305455

C 5.620633 -1.468640 -2.389653

H 5.583927 -2.023213 -3.331244

C 4.438347 -1.086151 -1.760336

H 3.481040 -1.350330 -2.220371

C 3.231733 0.874283 1.548628

C 3.417450 0.191524 2.761797

H 3.380338 -0.902457 2.772410

C 3.639642 0.864274 3.963020

H 3.773835 0.297226 4.888446

C 3.681603 2.258064 3.986768

H 3.847572 2.791393 4.926401

C 3.498995 2.962146 2.797915

H 3.519577 4.055349 2.801004

C 3.279604 2.275577 1.602832

H 3.132963 2.855076 0.686379

C -3.599031 3.760852 2.292312

H -3.295997 4.105002 3.292178

C -4.628255 4.226243 0.330915

H -5.175988 4.955415 -0.284876

C -4.350991 2.951837 -0.165427

H -4.694347 2.714831 -1.183009

C -2.709309 -1.311573 -0.861835

C -2.046216 -1.959208 -1.960372

C -2.541413 -3.154311 -2.482294

H -2.010335 -3.628419 -3.314900

C -3.688634 -3.759345 -1.978198

H -4.059520 -4.697569 -2.397921

C -4.358019 -3.129847 -0.930510

H -5.267191 -3.589804 -0.531185

C -3.904071 -1.938430 -0.368158

C -0.807563 -1.352589 -2.601556

H -0.578823 -0.424400 -2.057327

C -1.069921 -0.928205 -4.050189

H -0.192897 -0.410646 -4.474024

H -1.932484 -0.247345 -4.103922

H -1.289723 -1.799081 -4.690015

C 0.422386 -2.259699 -2.503897

H 1.322217 -1.736926 -2.867492

H 0.296031 -3.172420 -3.109547

H 0.609811 -2.571470 -1.466184

C -4.690561 -1.296785 0.762776

H -4.021456 -0.572382 1.250391

C -5.876733 -0.496285 0.215468

H -6.395208 0.045922 1.023008

H -6.602195 -1.163606 -0.282146

H -5.530848 0.250690 -0.511376

C -5.153482 -2.295813 1.827690

H -5.581981 -1.763421 2.692220

H -4.317014 -2.915191 2.186793

H -5.932621 -2.978067 1.448683

B 3.022033 0.062848 0.145778

C -3.642173 1.996240 0.588087

H -2.947089 0.669282 0.074093

C -3.288510 2.468024 1.866036

H -2.729134 1.821170 2.559842

N -4.256506 4.649278 1.542954

**^5^TS^α^_4_**

Imaginary frequency: -128.36 cm^-1^

Atom X Y Z

Fe -0.637853 0.446397 0.256596

N 1.750801 -0.816196 -1.171427

N 0.344027 -0.263346 -2.722871

N 1.678158 -1.166156 1.424813

N 0.288952 -0.931079 3.071552

N -2.482452 -0.453095 0.302108

H -3.073269 0.295741 0.649065

C 0.533324 -0.534309 1.784877

C 2.135354 -1.942504 2.465948

H 3.048168 -2.524103 2.402427

C 1.266945 -1.808667 3.496010

H 1.283340 -2.264731 4.477983

C -0.840914 -0.483389 3.924045

C -2.119623 -1.226854 3.530376

H -2.964666 -0.869969 4.139806

H -2.007000 -2.308610 3.700170

H -2.347528 -1.056588 2.466768

C -0.520655 -0.758276 5.398233

H -1.323885 -0.330129 6.014958

H 0.428691 -0.291934 5.702606

H -0.477729 -1.834271 5.622663

C -1.011116 1.021223 3.739001

H -1.812552 1.395581 4.393934

H -1.270292 1.260856 2.701611

H -0.077142 1.549226 3.984200

C 0.573215 -0.176176 -1.383007

C 2.251095 -1.286435 -2.367507

H 3.189457 -1.826215 -2.435291

C 1.374353 -0.945623 -3.340109

H 1.403721 -1.139961 -4.406563

C -0.807745 0.251526 -3.505129

C -1.675117 1.163658 -2.650318

H -2.507040 1.537502 -3.266810

H -1.072286 2.018330 -2.307697

H -2.106237 0.605298 -1.807390

C -1.637948 -0.940180 -3.996335

H -2.466258 -0.583806 -4.627298

H -2.070551 -1.485482 -3.143570

H -1.032808 -1.641210 -4.592442

C -0.261121 1.081281 -4.675306

H -1.101103 1.518875 -5.235442

H 0.331872 0.481707 -5.381943

H 0.356227 1.895663 -4.268777

C 3.629132 -2.155185 0.022392

C 5.022572 -2.021000 0.081871

H 5.456277 -1.027732 0.223881

C 5.876189 -3.122549 -0.036188

H 6.958994 -2.977103 0.014351

C 5.353329 -4.400417 -0.216984

H 6.018067 -5.263545 -0.310442

C 3.967693 -4.565658 -0.275670

H 3.538031 -5.561572 -0.414690

C 3.131411 -3.458754 -0.156383

H 2.049395 -3.608523 -0.197907

C 3.421583 0.497662 0.382780

C 3.699361 1.004722 1.659851

H 3.228205 0.545641 2.534417

C 4.574008 2.075296 1.854689

H 4.766974 2.443823 2.866449

C 5.205114 2.669119 0.764146

H 5.894393 3.505323 0.910363

C 4.925293 2.201528 -0.519617

H 5.382867 2.681203 -1.388545

C 4.040738 1.141657 -0.700265

H 3.827336 0.802915 -1.718130

C -3.254472 -1.446328 -0.231576

C -2.672321 -2.662919 -0.716142

C -3.481426 -3.645252 -1.282661

H -3.014662 -4.563277 -1.649527

C -4.862921 -3.502801 -1.389144

H -5.475614 -4.287589 -1.838718

C -5.442715 -2.335709 -0.900849

H -6.526698 -2.216334 -0.975049

C -4.682624 -1.316432 -0.329297

C -1.184069 -2.911843 -0.549572

H -0.691766 -1.933226 -0.567521

C -0.554175 -3.736478 -1.673832

H 0.542883 -3.697543 -1.607543

H -0.833885 -3.348297 -2.663484

H -0.850823 -4.797835 -1.627652

C -0.887759 -3.534669 0.818468

H 0.195976 -3.629068 0.992763

H -1.343143 -4.536476 0.896260

H -1.297990 -2.909793 1.620872

C -5.354846 -0.031981 0.136519

H -4.825196 0.314607 1.042045

C -5.226425 1.066995 -0.928383

H -5.635811 2.028790 -0.575960

H -5.774152 0.773822 -1.838377

H -4.177286 1.222659 -1.215127

C -6.817273 -0.194692 0.555334

H -7.185959 0.735382 1.016951

H -6.938101 -1.011872 1.282546

H -7.470673 -0.409006 -0.305678

B 2.589306 -0.883894 0.159527

N 0.888074 3.169210 -2.056117

C 0.792345 3.045772 -0.701037

C 1.741637 3.747418 0.079159

H 1.711413 3.641923 1.169815

C 2.712897 4.563461 -0.488509

H 3.432434 5.101276 0.137263

C 2.767190 4.664245 -1.878742

H 3.512598 5.282897 -2.385660

C 1.835664 3.928941 -2.606297

H 1.854025 3.967520 -3.706019

N -1.724327 2.437921 0.363371

H -2.354435 2.259767 -0.418608

H -0.740790 2.733049 -0.082728

C -2.311985 3.540779 1.135396

C -2.776127 4.640629 0.163545

H -3.195756 5.507604 0.700114

H -1.928325 4.982187 -0.449780

H -3.554162 4.253645 -0.515807

C -1.256978 4.136172 2.072686

H -0.429806 4.569067 1.492963

H -1.701166 4.929259 2.695033

H -0.833631 3.372607 2.736508

C -3.521121 3.034311 1.932764

H -3.228092 2.234837 2.626788

H -3.987952 3.846816 2.513756

H -4.283869 2.624832 1.252301

**^3^TS^γ^_4_**

Imaginary frequency: -512.10 cm^-1^

Atom X Y Z

Fe -0.250613 -0.262848 -0.024749

N 2.429526 -0.140803 1.289986

N 0.955579 -0.670438 2.790846

N 2.378922 0.644653 -1.149733

N 0.830534 1.378050 -2.480681

N -1.868718 0.765088 0.588684

C 1.033813 0.637167 -1.339768

C 3.000338 1.367083 -2.139730

H 4.076385 1.499199 -2.176116

C 2.040222 1.835972 -2.967418

H 2.128535 2.460831 -3.847522

C -0.438072 1.831667 -3.117624

C -0.750856 3.245383 -2.618227

H -1.664446 3.626532 -3.097964

H 0.077506 3.934025 -2.846781

H -0.918676 3.247337 -1.533510

C -0.254069 1.835226 -4.644434

H -1.228560 2.023075 -5.117290

H 0.119436 0.862130 -4.997636

H 0.428827 2.622739 -4.993656

C -1.590829 0.891188 -2.798465

H -2.507109 1.277783 -3.266777

H -1.774100 0.827369 -1.719482

H -1.390966 -0.111339 -3.202852

C 1.124105 -0.461555 1.447615

C 3.063778 -0.115701 2.508862

H 4.118570 0.117301 2.610989

C 2.146307 -0.438009 3.452831

H 2.259147 -0.520923 4.526481

C -0.287544 -1.114007 3.481690

C -1.190491 0.089014 3.757744

H -2.079848 -0.236313 4.319071

H -1.522566 0.535069 2.808213

H -0.656856 0.842854 4.355649

C 0.090599 -1.788408 4.808198

H -0.818394 -2.219344 5.250566

H 0.498684 -1.076644 5.540959

H 0.816211 -2.602369 4.657643

C -1.006612 -2.146205 2.617195

H -1.862168 -2.552231 3.174852

H -0.334083 -2.975302 2.359843

H -1.409420 -1.721259 1.689746

C 4.649250 0.596045 0.166697

C 5.863107 -0.105317 0.186157

H 5.859398 -1.180757 -0.010668

C 7.079296 0.529406 0.453902

H 8.005488 -0.051958 0.461258

C 7.113623 1.898016 0.710101

H 8.062702 2.398903 0.919115

C 5.920619 2.622999 0.695740

H 5.929644 3.698178 0.894425

C 4.715719 1.976190 0.429378

H 3.793012 2.561385 0.418730

C 3.416171 -1.686147 -0.575771

C 3.850237 -1.953644 -1.884910

H 4.010391 -1.120714 -2.576392

C 4.064666 -3.251511 -2.342321

H 4.390397 -3.420775 -3.372340

C 3.851055 -4.336332 -1.490548

H 4.007054 -5.358230 -1.845856

C 3.431790 -4.100179 -0.183794

H 3.253207 -4.939510 0.493674

C 3.223760 -2.792784 0.260978

H 2.884642 -2.636239 1.289388

C -6.072063 -2.360237 -0.718953

H -6.745539 -2.297572 -1.585762

C -5.831727 -2.663347 1.510120

H -6.309413 -2.850397 2.482548

C -4.446171 -2.510072 1.427408

H -3.875115 -2.579184 2.361603

C -2.244309 2.080142 0.483551

C -1.383420 3.134846 0.917890

C -1.747082 4.466504 0.711302

H -1.066079 5.255137 1.047965

C -2.940934 4.820279 0.093095

H -3.201510 5.868834 -0.068729

C -3.808197 3.799914 -0.296238

H -4.760745 4.064917 -0.762380

C -3.498807 2.455317 -0.109301

C -0.082582 2.841609 1.646363

H 0.009071 1.751330 1.715782

C -0.105965 3.389999 3.077956

H 0.780554 3.052327 3.640729

H -1.005778 3.054982 3.614089

H -0.107656 4.492926 3.084589

C 1.150379 3.355179 0.900168

H 2.065479 3.075157 1.445173

H 1.136547 4.453995 0.806545

H 1.215349 2.929973 -0.111041

C -4.505796 1.374515 -0.466382

H -3.948404 0.490083 -0.815764

C -5.288671 0.946981 0.783028

H -5.967433 0.108757 0.564504

H -5.881651 1.795451 1.163249

H -4.609337 0.623906 1.585500

C -5.463029 1.737159 -1.600943

H -6.049530 0.849914 -1.886598

H -4.919486 2.094640 -2.490075

H -6.180414 2.520591 -1.306188

B 3.206058 -0.147704 -0.071965

C -3.798076 -2.267045 0.202990

H -2.668631 0.146744 0.468415

C -4.696084 -2.198853 -0.880371

H -4.319107 -2.013137 -1.897157

N -6.653344 -2.591704 0.460635

N -1.343629 -1.821760 -0.938417

H -1.788002 -1.343004 -1.722066

C -0.755959 -3.056169 -1.487616

H -2.334318 -2.067717 -0.292476

C 0.395033 -2.714450 -2.438392

H 0.852540 -3.623650 -2.861104

H 0.038169 -2.090252 -3.275066

H 1.180278 -2.155197 -1.913608

C -0.233034 -3.894025 -0.320535

H -1.058429 -4.155010 0.359743

H 0.237877 -4.822161 -0.680647

H 0.523326 -3.326849 0.240448

C -1.823720 -3.857114 -2.251286

H -2.667974 -4.110552 -1.593848

H -2.219988 -3.266383 -3.094772

H -1.401731 -4.788574 -2.662893

**^5^TS^γ^_4_**

Imaginary frequency: -70.57 cm^-1^

Atom X Y Z

Fe -0.308968 -0.280317 -0.051950

N 2.412548 -0.467187 1.334745

N 0.968860 -1.200063 2.776555

N 2.386948 0.896889 -0.893644

N 0.932840 1.818545 -2.209513

N -1.871771 0.745428 0.671063

C 1.074786 0.856740 -1.249242

C 3.042257 1.870974 -1.611139

H 4.100032 2.075750 -1.487321

C 2.137447 2.456316 -2.431104

H 2.259625 3.270303 -3.137032

C -0.282035 2.212057 -2.969933

C -0.733174 3.596345 -2.495412

H -1.622709 3.917629 -3.057060

H 0.058182 4.347278 -2.644411

H -0.995680 3.572637 -1.429655

C 0.074416 2.238940 -4.465082

H -0.834704 2.436719 -5.051514

H 0.488467 1.269486 -4.781233

H 0.801978 3.025052 -4.713342

C -1.403367 1.201104 -2.771857

H -2.280270 1.524716 -3.350676

H -1.704678 1.127446 -1.719914

H -1.092550 0.211062 -3.133574

C 1.096970 -0.763969 1.489040

C 3.091989 -0.718548 2.505373

H 4.161304 -0.566233 2.604036

C 2.193550 -1.169068 3.414352

H 2.343952 -1.463006 4.445490

C -0.298304 -1.635013 3.426504

C -1.163063 -0.408560 3.722211

H -2.094525 -0.720386 4.219538

H -1.428523 0.114726 2.789741

H -0.627600 0.285415 4.387982

C 0.023907 -2.360284 4.738730

H -0.916399 -2.734051 5.167294

H 0.478086 -1.691318 5.484872

H 0.688983 -3.222717 4.577842

C -1.025765 -2.612600 2.505058

H -1.899895 -3.029010 3.025295

H -0.361798 -3.441765 2.222638

H -1.395451 -2.134721 1.589230

C 4.638622 0.459242 0.369575

C 5.843474 -0.186619 0.061636

H 5.815944 -1.136462 -0.478746

C 7.082733 0.346628 0.428722

H 8.001382 -0.187805 0.171233

C 7.149582 1.553228 1.120462

H 8.116581 1.973928 1.408911

C 5.965103 2.219204 1.442521

H 5.998901 3.167092 1.986509

C 4.738303 1.674261 1.071821

H 3.821384 2.208682 1.332340

C 3.327839 -1.536309 -0.879322

C 3.674725 -1.487600 -2.240149

H 3.777687 -0.514322 -2.730314

C 3.880261 -2.642135 -2.992207

H 4.141746 -2.564871 -4.051139

C 3.739056 -3.897007 -2.398000

H 3.888266 -4.807153 -2.984759

C 3.397416 -3.974967 -1.050036

H 3.273693 -4.950662 -0.572419

C 3.201856 -2.808745 -0.307185

H 2.930254 -2.896879 0.749064

C -6.146504 -2.352881 -0.719376

H -6.845732 -2.350883 -1.568251

C -5.837376 -2.502496 1.514859

H -6.283387 -2.622450 2.512843

C -4.456223 -2.348293 1.378002

H -3.859960 -2.343249 2.299480

C -2.217964 2.072026 0.587008

C -1.344080 3.087955 1.080041

C -1.691655 4.433432 0.950927

H -1.011215 5.194014 1.346812

C -2.873933 4.835809 0.338537

H -3.122991 5.895001 0.240247

C -3.738666 3.851710 -0.137072

H -4.675623 4.154629 -0.611926

C -3.444427 2.494385 -0.026599

C -0.067419 2.730298 1.822788

H 0.084291 1.647842 1.720466

C -0.208880 3.025593 3.320593

H 0.666619 2.656349 3.880917

H -1.109443 2.546170 3.730485

H -0.296636 4.110028 3.502665

C 1.170386 3.421221 1.249675

H 2.073231 3.090801 1.785971

H 1.111228 4.517097 1.354316

H 1.304075 3.191265 0.183433

C -4.444931 1.449659 -0.491008

H -3.879897 0.557265 -0.806697

C -5.343659 1.021489 0.676658

H -6.017083 0.200720 0.386999

H -5.950301 1.876822 1.018501

H -4.743760 0.671350 1.529313

C -5.287332 1.864540 -1.697222

H -5.869750 1.001664 -2.056545

H -4.656012 2.225927 -2.524556

H -6.007000 2.661905 -1.449045

B 3.167648 -0.162013 -0.015811

C -3.840144 -2.190365 0.121522

H -2.684783 0.137974 0.573033

C -4.776744 -2.195272 -0.933099

H -4.439352 -2.071945 -1.973318

N -6.691808 -2.509331 0.489339

N -1.385307 -1.745933 -1.128819

H -1.832806 -1.201210 -1.868630

C -0.836842 -2.961929 -1.759751

H -2.309694 -1.991111 -0.482747

C 0.273142 -2.572454 -2.738320

H 0.726149 -3.460962 -3.205464

H -0.124283 -1.931548 -3.543684

H 1.070048 -2.017309 -2.225667

C -0.276261 -3.857852 -0.655904

H -1.081981 -4.165933 0.027325

H 0.190311 -4.758958 -1.083447

H 0.488214 -3.318291 -0.078527

C -1.944812 -3.713601 -2.513307

H -2.760366 -3.993995 -1.831114

H -2.372492 -3.079552 -3.308221

H -1.548081 -4.625658 -2.987943

**^5^TS_K_^α^_1-2_**

Imaginary frequency: -273.46 cm^-1^

Atom X Y Z

Fe 0.150456 0.426842 -0.680660

K 0.637456 -1.950549 1.646575

N -2.663107 1.218387 -0.087060

N -1.660519 3.095725 -0.515536

N -2.354051 -1.210258 -0.968652

N -0.901397 -2.148993 -2.279181

N 1.818693 0.223302 0.118760

B -3.015355 -0.309322 0.158641

C -1.479364 1.750022 -0.476207

C -3.564951 2.232203 0.149041

H -4.579162 2.042803 0.484317

C -2.946703 3.408745 -0.125793

H -3.330407 4.420359 -0.073591

C -0.651891 4.063831 -1.025726

C -1.160056 5.494007 -0.831540

H -1.353215 5.715303 0.228663

H -0.385927 6.190731 -1.181244

H -2.071985 5.695495 -1.412733

C 0.651585 3.886696 -0.250829

H 1.083523 2.889947 -0.392251

H 1.403272 4.613659 -0.589553

H 0.492139 4.029124 0.827535

C -0.448112 3.792401 -2.520450

H -1.389666 3.931550 -3.072868

H 0.307107 4.477132 -2.933022

H -0.102682 2.762822 -2.685372

C -1.061113 -1.155743 -1.365857

C -2.988899 -2.249723 -1.609197

H -4.032782 -2.491268 -1.437733

C -2.085796 -2.839593 -2.435523

H -2.208303 -3.682178 -3.105307

C 0.354280 -2.382448 -3.044203

C 0.633128 -1.141143 -3.898305

H 0.801172 -0.257533 -3.266270

H 1.540582 -1.292332 -4.500998

H -0.208987 -0.929668 -4.574231

C 1.499395 -2.628240 -2.063033

H 1.285782 -3.478967 -1.399024

H 2.428842 -2.850843 -2.607035

H 1.696453 -1.746090 -1.440534

C 0.186430 -3.600727 -3.953690

H -0.028644 -4.511797 -3.375856

H -0.605862 -3.454530 -4.702981

H 1.128075 -3.766903 -4.494507

C -4.637922 -0.453501 0.053573

C -5.288483 -0.139541 -1.154728

H -4.690051 0.175669 -2.015746

C -6.672034 -0.213045 -1.289271

H -7.142914 0.036348 -2.243515

C -7.457801 -0.601957 -0.202140

H -8.544413 -0.660673 -0.300233

C -6.842524 -0.909656 1.007792

H -7.446587 -1.209442 1.867785

C -5.451632 -0.834417 1.127934

H -4.995048 -1.075317 2.091480

C -2.440269 -0.805528 1.600861

C -1.898990 0.059415 2.562653

H -1.813872 1.127026 2.340340

C -1.461388 -0.404945 3.807633

H -1.044906 0.297507 4.533010

C -1.559065 -1.758558 4.127662

H -1.225515 -2.121569 5.102509

C -2.099360 -2.643717 3.189072

H -2.192074 -3.706041 3.429352

C -2.525197 -2.167752 1.948613

H -2.947767 -2.876121 1.228952

C 2.707465 1.255833 0.437923

C 3.701530 1.662983 -0.493474

C 4.565247 2.711863 -0.172609

H 5.322800 3.028094 -0.893973

C 4.483069 3.368077 1.051826

H 5.166431 4.187109 1.287223

C 3.528812 2.958994 1.978242

H 3.475703 3.466823 2.944333

C 2.647751 1.913765 1.696736

C 3.766242 0.993902 -1.857448

H 3.379225 -0.027268 -1.723112

C 5.176684 0.884093 -2.436654

H 5.870309 0.419494 -1.719900

H 5.167492 0.266277 -3.348310

H 5.590183 1.866480 -2.716157

C 2.830252 1.712968 -2.834275

H 1.802487 1.720508 -2.439752

H 3.138163 2.761764 -2.975000

H 2.813826 1.222665 -3.821066

C 1.645878 1.436053 2.731650

H 0.788437 1.044190 2.161798

C 2.246660 0.274583 3.536509

H 3.066604 0.636114 4.177245

H 1.494988 -0.197963 4.193179

H 2.685111 -0.484712 2.868226

C 1.116984 2.530406 3.657536

H 0.307701 2.139765 4.294317

H 1.896221 2.916431 4.333564

H 0.715183 3.381698 3.087250

C 3.639572 -4.385848 1.074233

C 4.980297 -4.332604 0.713424

C 5.493776 -3.097329 0.311902

C 4.642325 -1.998901 0.283424

C 3.280571 -2.102560 0.643829

N 2.823882 -3.325311 1.043324

H 6.546699 -3.001963 0.028844

H 3.199108 -5.334069 1.407725

H 5.603147 -5.228737 0.753555

H 5.029031 -1.021241 -0.020845

H 2.385393 -0.776659 0.322268

**^5^TS_K_^β^_1-2_**

Imaginary frequency: -1091.30 cm^-1^

Atom X Y Z

Fe 0.186374 0.185073 -0.831441

N -2.334616 -1.402099 -0.895149

N -0.927380 -2.525734 -2.102044

N -2.546902 1.098326 -0.182343

N -1.552716 2.892634 -0.894586

N 1.745175 0.175485 0.182131

B -2.927707 -0.400801 0.179383

C -1.044323 -1.436485 -1.303503

C -2.135844 -3.192149 -2.172857

H -2.283834 -4.100347 -2.748188

C -3.012101 -2.481553 -1.416042

H -4.063407 -2.661434 -1.214482

C 0.299610 -2.988759 -2.794293

C 0.730109 -4.318334 -2.163122

H 0.941844 -4.182290 -1.092218

H 1.643849 -4.691790 -2.647888

H -0.049366 -5.087417 -2.269134

C 1.419956 -1.962728 -2.639900

H 1.136717 -0.984333 -3.061496

H 2.304945 -2.309653 -3.192233

H 1.723346 -1.826418 -1.592537

C -0.020063 -3.168157 -4.283602

H 0.886726 -3.475203 -4.824049

H -0.378597 -2.222768 -4.716855

H -0.785624 -3.938567 -4.454608

C -1.404724 1.551416 -0.754244

C -3.383905 2.163180 0.074345

H -4.358282 2.039210 0.536179

C -2.768687 3.287645 -0.371223

H -3.108436 4.318262 -0.368781

C -0.657733 3.827575 -1.627957

C -0.190150 4.934144 -0.677827

H 0.402942 5.673060 -1.235631

H 0.449241 4.517850 0.112324

H -1.036524 5.461901 -0.213446

C 0.559113 3.089598 -2.175658

H 1.190503 3.803016 -2.723756

H 0.266684 2.290270 -2.872930

H 1.177222 2.671130 -1.369813

C -1.459721 4.415770 -2.797855

H -1.841171 3.612188 -3.445385

H -0.813373 5.070135 -3.400316

H -2.314544 5.015669 -2.453181

C -4.555544 -0.512129 0.136913

C -5.334252 -0.903603 1.232987

H -4.847106 -1.155241 2.178911

C -6.728120 -0.979618 1.154767

H -7.305648 -1.287276 2.030091

C -7.380178 -0.663418 -0.033606

H -8.469276 -0.722106 -0.098601

C -6.628612 -0.267447 -1.142283

H -7.129085 -0.013854 -2.080227

C -5.241613 -0.193328 -1.049926

H -4.668547 0.124055 -1.927316

C -2.339555 -0.814470 1.643193

C -2.045798 0.134816 2.632800

H -2.123827 1.198148 2.391886

C -1.661536 -0.237247 3.924232

H -1.451001 0.532345 4.671036

C -1.561722 -1.586910 4.266162

H -1.279122 -1.881940 5.279561

C -1.846529 -2.556171 3.298889

H -1.786808 -3.617559 3.554678

C -2.222424 -2.168184 2.010670

H -2.448633 -2.943820 1.272283

C 2.434886 1.372408 0.318224

C 3.541079 1.679770 -0.529747

C 4.172149 2.919683 -0.429304

H 5.006742 3.147873 -1.099336

C 3.765204 3.875989 0.496687

H 4.272057 4.841271 0.559590

C 2.713812 3.571178 1.356785

H 2.412251 4.307867 2.106953

C 2.047439 2.346740 1.290360

C 4.000877 0.686445 -1.586002

H 3.432942 -0.236446 -1.421285

C 5.481018 0.324795 -1.444571

H 6.137177 1.193317 -1.618974

H 5.760578 -0.454395 -2.171806

H 5.690058 -0.064646 -0.437219

C 3.664767 1.176684 -2.998447

H 3.960697 0.433068 -3.756504

H 4.181736 2.121214 -3.234728

H 2.583173 1.355381 -3.102950

C 0.984575 1.998384 2.320173

H 0.385385 1.188633 1.879981

C 0.003218 3.120736 2.655683

H 0.506279 4.021061 3.041638

H -0.703652 2.791859 3.434444

H -0.585533 3.409111 1.772444

C 1.663173 1.461913 3.588606

H 2.214571 2.264558 4.103203

H 2.408384 0.686826 3.345469

H 0.931792 1.046822 4.302738

C 3.390263 -1.943969 0.597103

C 3.816777 -3.115192 -0.051418

C 4.930836 -3.826873 0.396291

H 3.284857 -3.488476 -0.935415

H 5.270352 -4.735865 -0.107895

H 2.463019 -0.833863 0.243896

H 3.965140 -0.654853 2.243141

C 4.192770 -1.585833 1.695971

K 0.937452 -1.502783 2.145919

C 5.615694 -3.350908 1.514745

H 6.492060 -3.887158 1.898474

N 5.259896 -2.245159 2.164154

**^5^TS_K_^γ^_1-2_**

Imaginary frequency: -936.39 cm^-1^

Atom X Y Z

Fe -0.168154 -0.318712 -0.849955

N 2.448931 1.147433 -0.907430

N 1.147283 2.259751 -2.239217

N 2.533252 -1.322549 -0.077097

N 1.406719 -3.100569 -0.605884

N -1.815064 -0.157308 -0.010396

B 2.986127 0.165968 0.218796

C 1.174847 1.218116 -1.367323

C 2.392063 2.853017 -2.307668

H 2.611133 3.707438 -2.939737

C 3.200242 2.153627 -1.470866

H 4.251377 2.294969 -1.241097

C 0.009649 2.697832 -3.087101

C -0.265333 4.179367 -2.804470

H -0.486302 4.334714 -1.739118

H -1.135601 4.516883 -3.384755

H 0.588272 4.816721 -3.077259

C -1.239488 1.878480 -2.782639

H -1.089499 0.812782 -3.017520

H -2.063215 2.233789 -3.417326

H -1.573962 1.965707 -1.739874

C 0.404985 2.490518 -4.555358

H -0.425438 2.786978 -5.212203

H 0.635643 1.431478 -4.743866

H 1.284954 3.089050 -4.832663

C 1.345811 -1.746015 -0.570678

C 3.314229 -2.413490 0.235619

H 4.312698 -2.315573 0.649230

C 2.614154 -3.529416 -0.092250

H 2.893959 -4.573327 -0.012071

C 0.314596 -4.007360 -1.049744

C -0.572214 -4.328318 0.155198

H -1.387595 -5.006266 -0.136146

H -1.033096 -3.412279 0.548542

H 0.012182 -4.808324 0.955257

C -0.509996 -3.327842 -2.142870

H -1.249955 -4.042073 -2.530060

H 0.126017 -2.993157 -2.975699

H -1.076004 -2.471027 -1.753354

C 0.930575 -5.289375 -1.621686

H 1.649643 -5.064435 -2.423614

H 0.130356 -5.914080 -2.041985

H 1.435762 -5.890147 -0.851984

C 4.617939 0.189188 0.187675

C 5.411117 0.536421 1.288248

H 4.933979 0.826240 2.228182

C 6.807691 0.517601 1.223753

H 7.395945 0.793236 2.102559

C 7.448468 0.147024 0.045142

H 8.539588 0.131839 -0.009255

C 6.682526 -0.209469 -1.067094

H 7.173545 -0.506883 -1.997083

C 5.292997 -0.189139 -0.988199

H 4.708662 -0.478613 -1.867791

C 2.394739 0.669702 1.653732

C 1.837187 -0.193791 2.608606

H 1.733472 -1.257773 2.376658

C 1.404974 0.267842 3.856717

H 0.978748 -0.433620 4.577438

C 1.520454 1.618381 4.186759

H 1.193009 1.978733 5.164706

C 2.072577 2.502471 3.253980

H 2.180074 3.561776 3.501563

C 2.496030 2.028374 2.011182

H 2.930316 2.736253 1.298168

C -2.680900 -1.215482 0.251254

C -3.550692 -1.720695 -0.765001

C -4.441236 -2.753046 -0.466598

H -5.110248 -3.127950 -1.244473

C -4.505169 -3.319897 0.803242

H -5.217396 -4.119593 1.017794

C -3.642043 -2.858688 1.790364

H -3.679208 -3.314084 2.783208

C -2.727500 -1.835150 1.535610

C -3.506534 -1.124053 -2.162784

H -2.457281 -0.830897 -2.333329

C -4.337866 0.159489 -2.257892

H -5.397735 -0.049486 -2.039786

H -4.279241 0.590726 -3.271217

H -3.988388 0.912978 -1.539940

C -3.898461 -2.101109 -3.272436

H -3.676889 -1.663317 -4.258346

H -4.976361 -2.328409 -3.258942

H -3.354065 -3.054044 -3.191998

C -1.787848 -1.365660 2.632910

H -0.912129 -0.947170 2.110774

C -1.271012 -2.485887 3.537369

H -2.070046 -2.921283 4.157420

H -0.505560 -2.100984 4.229300

H -0.819020 -3.298874 2.950905

C -2.438526 -0.254948 3.469007

H -3.274056 -0.661292 4.060287

H -2.871279 0.532486 2.831881

H -1.722668 0.194758 4.181114

C -3.139428 2.207755 0.416731

C -5.192910 3.033085 1.456524

C -2.819845 3.572878 0.298357

H -6.163247 2.832602 1.928635

C -3.682344 4.572886 0.759678

H -1.880937 3.898130 -0.174972

H -3.411251 5.632279 0.655330

H -2.385854 0.917996 0.071538

H -4.748959 0.951850 1.165296

N -4.854546 4.319257 1.336109

C -4.387603 1.978337 1.026136

K -0.655100 1.753462 1.744058

**^5^TS_K_^α^_2-3_**

Imaginary frequency: -775.78 cm^-1^

Atom X Y Z

Fe -0.083008 0.379902 -0.147384

N 2.588475 -0.656437 -1.115143

N 1.244011 -0.921153 -2.794226

N 2.503985 0.029070 1.419296

N 0.999791 0.382972 2.936070

N -1.780942 -0.754724 -0.112315

H -2.464042 -0.083702 -0.458422

C 1.181483 0.267972 1.582641

C 3.137112 0.002927 2.641184

H 4.205939 -0.154002 2.738942

C 2.202049 0.214465 3.596765

H 2.311849 0.261043 4.673100

C -0.297706 0.473122 3.643360

C -0.889016 -0.939867 3.690866

H -1.823974 -0.954143 4.277125

H -0.190595 -1.636115 4.176241

H -1.059237 -1.311161 2.669088

C -0.083089 0.985331 5.072831

H -1.062889 1.157228 5.540522

H 0.463551 1.939505 5.074071

H 0.456648 0.264992 5.703711

C -1.232321 1.444719 2.927686

H -2.192752 1.515372 3.461477

H -1.434761 1.159062 1.888086

H -0.797181 2.450061 2.905992

C 1.341170 -0.355890 -1.553535

C 3.248358 -1.415881 -2.053767

H 4.265377 -1.765243 -1.911383

C 2.411609 -1.590341 -3.104908

H 2.564862 -2.130999 -4.031221

C 0.075476 -0.901340 -3.710090

C 0.578679 -0.832246 -5.158906

H -0.279510 -0.696276 -5.832007

H 1.089874 -1.753166 -5.472369

H 1.265359 0.015990 -5.297554

C -0.775860 0.334900 -3.439605

H -1.560440 0.412808 -4.205964

H -0.165763 1.248419 -3.459378

H -1.267038 0.252185 -2.464059

C -0.757682 -2.170478 -3.499484

H -1.605463 -2.188488 -4.200636

H -1.157863 -2.208392 -2.477018

H -0.152253 -3.073188 -3.670726

C 4.700583 -0.867305 0.379672

C 6.000136 -0.378918 0.197036

H 6.140867 0.649631 -0.146048

C 7.124770 -1.172779 0.442068

H 8.125180 -0.759752 0.289134

C 6.973563 -2.485512 0.880891

H 7.850492 -3.108782 1.072590

C 5.688801 -2.997126 1.076869

H 5.555597 -4.025033 1.424163

C 4.578224 -2.194514 0.830143

H 3.578346 -2.608643 0.996396

C 3.707944 1.560636 -0.328830

C 3.677967 2.618124 0.591057

H 3.376196 2.426598 1.624983

C 4.010930 3.924245 0.227871

H 3.965688 4.723743 0.972060

C 4.395768 4.209996 -1.079891

H 4.655674 5.230920 -1.370106

C 4.441126 3.176517 -2.015797

H 4.738077 3.385601 -3.046808

C 4.100954 1.878326 -1.639951

H 4.134227 1.088422 -2.396684

C -2.307453 -2.023269 -0.149741

C -1.550000 -3.158276 0.287522

C -2.141854 -4.421244 0.298120

H -1.542032 -5.276920 0.619847

C -3.461863 -4.632924 -0.087880

H -3.897255 -5.633623 -0.070206

C -4.210118 -3.537701 -0.504654

H -5.244766 -3.693949 -0.818291

C -3.675370 -2.248119 -0.543100

C -0.086430 -3.041819 0.689749

H 0.135460 -1.978145 0.854701

C 0.837223 -3.530109 -0.430586

H 1.894859 -3.397650 -0.158558

H 0.664995 -2.977612 -1.360208

H 0.665751 -4.599653 -0.634535

C 0.248295 -3.782599 1.990658

H 1.240400 -3.475574 2.358319

H 0.280545 -4.873464 1.840572

H -0.487840 -3.584314 2.783195

C -4.532031 -1.096899 -1.068204

H -4.227432 -0.159436 -0.574430

C -4.292927 -0.876296 -2.566420

H -4.837252 0.010417 -2.927741

H -4.629622 -1.753812 -3.141003

H -3.225418 -0.731163 -2.774555

C -6.029164 -1.232486 -0.776825

H -6.544504 -0.294437 -1.031678

H -6.227657 -1.443306 0.287578

H -6.497126 -2.038843 -1.362435

B 3.359796 0.022958 0.086967

N -1.021054 2.208443 -0.581451

H -2.137028 2.204964 -0.015643

C -0.334325 3.519513 -0.610202

C -5.695129 1.953383 1.362959

H -6.218985 1.593432 2.259638

C -6.424861 2.544789 0.339247

H -7.507726 2.657390 0.419398

C -5.718588 2.974162 -0.784706

H -6.243178 3.433489 -1.627844

C -4.338755 2.807469 -0.801078

H -3.770712 3.152734 -1.671207

N -4.367231 1.772822 1.333177

C -3.634113 2.205969 0.266175

H -1.357939 2.060241 -1.536497

K -3.618047 -0.689109 2.061724

C -1.318760 4.627059 -1.017447

H -1.723371 4.437259 -2.025706

H -2.164613 4.684026 -0.316272

H -0.819348 5.608409 -1.040112

C 0.214925 3.830499 0.781874

H 0.868126 3.019100 1.135284

H 0.806971 4.757917 0.765832

H -0.609879 3.963516 1.498085

C 0.815259 3.476621 -1.621631

H 1.369281 4.427448 -1.642404

H 1.529743 2.679029 -1.375576

H 0.430447 3.291086 -2.638341

**^5^TS_K_^β^_2-3_**

Imaginary frequency: -722.25 cm^-1^

Atom X Y Z

Fe -0.018738 0.527014 0.227337

N -2.649367 -0.710715 1.109789

N -1.434383 -0.679775 2.902193

N -2.396391 -0.342649 -1.470899

N -0.819819 0.009365 -2.912461

N 1.730289 -0.481898 0.588459

H 2.330756 0.265656 0.926487

C -1.116797 0.090096 -1.577138

C -2.889759 -0.677706 -2.710550

H -3.904849 -1.028645 -2.859257

C -1.906882 -0.466631 -3.617867

H -1.916209 -0.613098 -4.690509

C 0.483518 0.341327 -3.534857

C 1.496284 -0.738046 -3.142022

H 2.469818 -0.490359 -3.596791

H 1.169889 -1.719664 -3.513895

H 1.590443 -0.790605 -2.047113

C 0.354587 0.361649 -5.061827

H 1.317509 0.675071 -5.489270

H -0.406957 1.082286 -5.393316

H 0.114656 -0.628064 -5.477342

C 0.926479 1.725890 -3.071542

H 1.903657 1.981394 -3.505972

H 1.022608 1.784998 -1.982533

H 0.191584 2.478991 -3.388176

C -1.475511 -0.247160 1.605701

C -3.316972 -1.436038 2.069298

H -4.285455 -1.889953 1.890155

C -2.557921 -1.429854 3.190525

H -2.739290 -1.890796 4.154171

C -0.370567 -0.455484 3.912842

C -1.024569 -0.247170 5.287037

H -0.252572 0.047079 6.012043

H -1.497946 -1.160020 5.674671

H -1.781148 0.550256 5.244515

C 0.422248 0.800492 3.569621

H 1.132690 1.016489 4.380444

H -0.243869 1.663794 3.439144

H 0.995547 0.646538 2.650466

C 0.553384 -1.678078 3.947500

H 1.306664 -1.564172 4.741271

H 1.077687 -1.791553 2.988482

H -0.018253 -2.597280 4.145397

C -4.634322 -1.217455 -0.491402

C -5.956218 -0.758125 -0.539041

H -6.157406 0.305749 -0.387834

C -7.027979 -1.624164 -0.776099

H -8.047441 -1.231039 -0.806748

C -6.800293 -2.983366 -0.973555

H -7.635846 -3.663017 -1.158334

C -5.491474 -3.469602 -0.934497

H -5.297742 -4.534222 -1.089389

C -4.434089 -2.595099 -0.698612

H -3.414376 -2.993656 -0.675777

C -3.802703 1.324602 -0.027177

C -3.737210 2.263540 -1.065547

H -3.336878 1.963783 -2.038971

C -4.160542 3.583269 -0.895926

H -4.083532 4.290181 -1.726160

C -4.675291 4.000391 0.329125

H -5.006195 5.032567 0.467682

C -4.759859 3.084987 1.378714

H -5.158778 3.398971 2.346778

C -4.327359 1.772905 1.196874

H -4.394033 1.076284 2.038564

C 2.448311 -1.628790 0.537703

C 1.904792 -2.825832 -0.055613

C 2.698885 -3.965797 -0.172890

H 2.267037 -4.861067 -0.627458

C 4.016042 -4.015041 0.291840

H 4.604360 -4.931417 0.214528

C 4.536728 -2.878431 0.912702

H 5.553955 -2.916291 1.309503

C 3.809013 -1.692469 1.029077

C 0.443741 -2.880027 -0.469391

H 0.138160 -1.858744 -0.734609

C -0.432589 -3.319623 0.708761

H -1.499473 -3.275836 0.443788

H -0.284842 -2.668514 1.576967

H -0.188095 -4.351851 1.007767

C 0.155900 -3.764160 -1.685211

H -0.862421 -3.569053 -2.055835

H 0.212462 -4.836701 -1.438629

H 0.858103 -3.578030 -2.511897

C 4.438029 -0.483355 1.714303

H 4.085077 0.424498 1.199326

C 3.991766 -0.388933 3.178352

H 4.380822 0.525710 3.652797

H 4.358028 -1.257689 3.748381

H 2.898584 -0.375353 3.257347

C 5.964385 -0.428653 1.621411

H 6.324815 0.542063 1.992789

H 6.315580 -0.528484 0.582497

H 6.448529 -1.211132 2.227339

B -3.358031 -0.231000 -0.218615

N 0.787671 2.448866 0.540739

H 1.892627 2.541135 -0.014870

C 0.046324 3.727692 0.495049

C 5.961139 1.846254 -1.267789

H 6.977458 1.570043 -1.569138

C 5.721738 3.022494 -0.564127

H 6.556607 3.681988 -0.312891

C 4.408872 3.325782 -0.185245

H 4.233350 4.247415 0.385357

C 3.334907 2.481546 -0.508067

H 1.121838 2.362151 1.504788

K 4.416278 -1.669334 -1.879815

C 0.979943 4.876165 0.911183

H 1.362534 4.716750 1.933183

H 1.842589 4.941490 0.231235

H 0.451762 5.842526 0.897136

C -0.440587 3.968157 -0.933595

H -1.065527 3.129655 -1.274861

H -1.048318 4.884285 -0.985538

H 0.412383 4.079201 -1.619151

C -1.155255 3.675171 1.442914

H -1.747658 4.601579 1.390033

H -1.820495 2.836935 1.189969

H -0.824035 3.549684 2.486926

N 4.979349 0.999023 -1.596366

C 3.735275 1.343822 -1.221420

H 2.960845 0.616704 -1.488208

**^5^TS_K_^γ^_2-3_**

Imaginary frequency: -794.80 cm^-1^

Atom X Y Z

Fe 0.120332 0.397551 0.319514

N -2.614025 -0.735133 1.038486

N -1.386272 -1.087659 2.788667

N -2.333611 0.011403 -1.460815

N -0.732317 0.467133 -2.844189

N 1.792759 -0.816908 0.330392

H 2.446437 -0.332402 0.940880

C -1.028003 0.371515 -1.509178

C -2.841633 -0.110526 -2.733997

H -3.876285 -0.371363 -2.926626

C -1.844103 0.163736 -3.606186

H -1.854168 0.170427 -4.688998

C 0.594112 0.735802 -3.439737

C 1.389175 -0.573725 -3.475984

H 2.379361 -0.409966 -3.935015

H 0.873774 -1.325164 -4.089458

H 1.497620 -0.994303 -2.465062

C 0.429289 1.271514 -4.867932

H 1.412703 1.580390 -5.250140

H -0.234138 2.148254 -4.889145

H 0.037753 0.513039 -5.560063

C 1.327697 1.792138 -2.623751

H 2.302564 2.000081 -3.090432

H 1.488245 1.489518 -1.581131

H 0.763761 2.733124 -2.598334

C -1.392665 -0.472590 1.567038

C -3.346479 -1.515343 1.904007

H -4.357558 -1.840319 1.684233

C -2.581810 -1.747692 2.996372

H -2.801570 -2.322052 3.888802

C -0.302357 -1.130585 3.801805

C -0.921708 -0.948923 5.196073

H -0.118787 -0.856066 5.941170

H -1.544758 -1.802156 5.497759

H -1.536058 -0.037228 5.232552

C 0.690655 0.002948 3.575783

H 1.440287 -0.008443 4.380124

H 0.182419 0.976461 3.577912

H 1.214099 -0.130116 2.623206

C 0.415552 -2.481752 3.710368

H 1.175138 -2.563909 4.502170

H 0.917853 -2.586699 2.738278

H -0.292181 -3.316211 3.824976

C -4.604632 -0.924553 -0.627055

C -5.906587 -0.408863 -0.643017

H -6.072329 0.632704 -0.355464

C -7.003264 -1.190442 -1.019377

H -8.005711 -0.754743 -1.020238

C -6.821785 -2.519272 -1.392977

H -7.676871 -3.132902 -1.686976

C -5.533996 -3.059755 -1.389302

H -5.375489 -4.100983 -1.681774

C -4.451705 -2.269029 -1.013021

H -3.450016 -2.709509 -1.020779

C -3.704130 1.488128 0.210908

C -3.577781 2.571462 -0.668934

H -3.155092 2.410966 -1.665336

C -3.966728 3.863758 -0.310788

H -3.842926 4.685617 -1.020890

C -4.508716 4.106817 0.948899

H -4.813414 5.116472 1.234904

C -4.656848 3.044928 1.841871

H -5.079927 3.220539 2.834322

C -4.258750 1.761879 1.472579

H -4.375566 0.948798 2.195687

C 2.304743 -2.050686 -0.008702

C 1.533652 -2.994622 -0.764451

C 2.139326 -4.150181 -1.252504

H 1.537005 -4.850406 -1.836084

C 3.476267 -4.454098 -1.008186

H 3.926495 -5.364162 -1.408469

C 4.209775 -3.593024 -0.202172

H 5.241635 -3.853069 0.041917

C 3.659726 -2.415500 0.314996

C 0.034112 -2.816739 -0.935527

H -0.181703 -1.741984 -0.905549

C -0.701854 -3.461506 0.243838

H -1.785668 -3.285843 0.181253

H -0.358379 -3.046370 1.197417

H -0.523973 -4.548942 0.259664

C -0.536163 -3.346119 -2.253869

H -1.555914 -2.958423 -2.401765

H -0.599784 -4.446164 -2.262070

H 0.067231 -3.043598 -3.120516

C 4.472696 -1.571802 1.294826

H 4.252480 -0.504186 1.123856

C 4.046206 -1.860205 2.740307

H 4.576915 -1.206820 3.451187

H 4.267003 -2.907161 2.999906

H 2.967875 -1.705276 2.871702

C 5.990112 -1.703158 1.151016

H 6.495725 -0.979458 1.807478

H 6.334178 -1.516046 0.120321

H 6.345324 -2.703599 1.440396

B -3.297436 -0.033190 -0.205287

N 0.960066 2.206243 0.983696

H 2.078054 2.478651 0.451971

C 0.207623 3.470597 1.145725

C 5.773546 1.917871 0.442016

H 6.509036 1.425326 1.090331

C 4.442446 2.055398 0.847959

H 4.160236 1.663011 1.834337

C 3.471963 2.650669 0.021317

H 1.285810 1.955783 1.920933

K 3.958789 -0.014660 -1.486608

C 1.105561 4.528470 1.808906

H 1.430596 4.189995 2.807030

H 2.007009 4.718447 1.207903

H 0.564492 5.478860 1.938570

C -0.208822 3.957238 -0.243160

H -0.814457 3.193572 -0.753946

H -0.812752 4.874295 -0.172648

H 0.681078 4.175290 -0.854505

C -1.033567 3.251109 2.013754

H -1.625854 4.174293 2.107816

H -1.685708 2.478765 1.584726

H -0.747575 2.932071 3.029861

C 4.002641 3.162378 -1.179263

H 3.360903 3.700485 -1.886553

C 5.345680 2.982623 -1.520563

H 5.735374 3.361563 -2.474383

N 6.223243 2.334305 -0.745230

**^5^TS_K(12-C-4)_^γ^_1-3_**

Imaginary frequency: -1368.27 cm^-1^

Atom X Y Z

Fe -2.040680 -0.253089 0.174915

N -0.967781 2.280142 1.289696

N -0.509486 0.786535 2.798928

N -2.158771 2.472122 -1.058854

N -3.441139 1.243785 -2.309541

N -2.728060 -1.978605 0.363915

C -2.583449 1.198214 -1.253474

C -2.752589 3.305397 -1.982863

H -2.555310 4.371592 -2.020328

C -3.559258 2.544066 -2.762415

H -4.193840 2.834529 -3.590582

C -4.201588 0.092215 -2.879082

C -5.422721 -0.181195 -1.999048

H -6.020006 -1.002866 -2.420492

H -6.058755 0.714435 -1.924096

H -5.123011 -0.489795 -0.990183

C -4.656365 0.443245 -4.300582

H -5.125820 -0.445101 -4.745012

H -3.807660 0.734688 -4.938082

H -5.407640 1.246530 -4.316203

C -3.298304 -1.139458 -2.944290

H -3.843716 -1.964725 -3.424344

H -3.010817 -1.479511 -1.940382

H -2.390295 -0.931396 -3.531053

C -0.962125 0.945612 1.526029

C -0.534601 2.950129 2.413786

H -0.458615 4.031750 2.454718

C -0.243526 2.022554 3.360600

H 0.109010 2.159984 4.375547

C -0.125011 -0.524254 3.391221

C -1.327399 -1.466378 3.447836

H -1.026762 -2.411876 3.923808

H -1.722097 -1.704501 2.446627

H -2.134823 -1.021634 4.047270

C 0.408179 -0.309029 4.810600

H 0.669608 -1.287195 5.237674

H -0.349986 0.146033 5.465325

H 1.314752 0.315425 4.827049

C 0.989438 -1.109598 2.514995

H 1.347382 -2.061552 2.936124

H 1.831906 -0.401146 2.455950

H 0.628638 -1.301453 1.496031

C -1.066999 4.556292 0.042448

C -0.065372 5.454949 -0.344448

H 0.845795 5.073039 -0.813514

C -0.198309 6.832748 -0.148231

H 0.601828 7.507518 -0.463879

C -1.347483 7.346730 0.446645

H -1.455660 8.423130 0.601345

C -2.362580 6.472696 0.842325

H -3.270342 6.863725 1.309043

C -2.218003 5.102641 0.639788

H -3.023494 4.430427 0.953089

C 0.455494 2.509392 -0.871523

C 0.604001 2.492100 -2.268289

H -0.269960 2.677439 -2.899999

C 1.837295 2.265587 -2.881558

H 1.914257 2.280026 -3.971691

C 2.974420 2.039224 -2.103397

H 3.950588 1.913220 -2.586352

C 2.847854 2.009184 -0.713056

H 3.710259 1.806738 -0.073687

C 1.607695 2.237376 -0.117631

H 1.544333 2.220432 0.973980

C 1.976615 -3.298593 -0.435028

H 2.815216 -3.933348 -0.120898

C 1.313230 -1.356491 -1.436130

H 1.585134 -0.413298 -1.928702

C -0.021457 -1.672127 -1.222983

H -0.773775 -0.960886 -1.599262

C -4.033348 -2.350160 0.463904

C -4.983668 -1.601315 1.243108

C -6.333609 -1.952411 1.235817

H -7.040028 -1.354108 1.821519

C -6.811467 -3.032135 0.500604

H -7.874196 -3.284294 0.500189

C -5.895815 -3.795959 -0.225006

H -6.262890 -4.656140 -0.790890

C -4.537448 -3.493418 -0.250096

C -4.543975 -0.412404 2.086537

H -3.444481 -0.373061 2.032038

C -4.888619 -0.603884 3.567426

H -4.455920 0.203836 4.181714

H -4.506161 -1.567126 3.937069

H -5.978453 -0.595816 3.731328

C -5.073863 0.934670 1.578721

H -4.695642 1.763732 2.199762

H -6.175388 0.964390 1.607993

H -4.763836 1.129674 0.541702

C -3.547836 -4.365632 -1.002466

H -2.801007 -3.692014 -1.452973

C -2.785405 -5.272258 -0.026864

H -1.985335 -5.829055 -0.542720

H -3.471648 -5.999382 0.438265

H -2.323746 -4.676156 0.773510

C -4.155669 -5.187263 -2.138939

H -3.360333 -5.687289 -2.714271

H -4.731034 -4.553629 -2.832245

H -4.830605 -5.975888 -1.767683

B -0.954710 2.939908 -0.152597

C -0.413521 -2.844594 -0.555099

H -1.723590 -2.686116 -0.104702

H 0.478848 -4.615362 0.347129

C 0.660232 -3.670715 -0.177972

N 2.316208 -2.152475 -1.045135

O 7.059210 -2.158330 -1.417558

O 5.191745 -2.435998 0.678734

O 5.284933 0.257337 1.053059

O 6.966812 0.668974 -1.049686

C 7.136153 -3.168796 -0.436568

H 8.182745 -3.489488 -0.285851

H 6.570840 -4.027809 -0.828793

C 6.532749 -2.763800 0.897763

H 6.628659 -3.608841 1.607920

H 7.094483 -1.912305 1.323883

C 4.472174 -1.866976 1.745018

H 4.428113 -2.549233 2.614638

H 3.448706 -1.742698 1.364041

C 5.034807 -0.523920 2.193473

H 4.309347 -0.037994 2.871572

H 5.974093 -0.655418 2.764830

C 6.223092 1.286238 1.197254

H 7.089586 0.923385 1.784745

H 5.807002 2.153909 1.743655

C 6.657088 1.740365 -0.192013

H 5.837663 2.287290 -0.680690

H 7.509802 2.438301 -0.095370

C 8.125134 -0.059004 -0.737003

H 8.146558 -0.338903 0.331238

H 9.032638 0.544055 -0.936187

C 8.164250 -1.308327 -1.598838

H 8.155993 -1.006363 -2.657673

H 9.111508 -1.846210 -1.411368

K 4.610163 -0.822792 -1.454867

**^5^TS_K(12-C-4)_^γ^_4_**

Imaginary frequency: -754.12 cm^-1^

Atom X Y Z

Fe 1.454370 0.559988 0.316461

N 3.522810 -0.195939 -1.816394

N 1.882417 0.673031 -2.930178

N 3.952625 -1.187187 0.557338

N 2.902110 -1.492212 2.424373

N -0.484871 -0.117781 0.453419

C 2.897053 -0.735738 1.284720

C 4.587299 -2.210733 1.221288

H 5.461001 -2.713668 0.822191

C 3.930531 -2.412302 2.387499

H 4.117990 -3.129065 3.179204

C 2.008848 -1.409364 3.605139

C 1.103189 -2.645097 3.625409

H 0.423829 -2.604704 4.490012

H 1.694756 -3.570215 3.699778

H 0.493982 -2.694878 2.713190

C 2.877350 -1.352486 4.871734

H 2.232407 -1.198146 5.749025

H 3.590881 -0.517019 4.813468

H 3.442212 -2.279983 5.040556

C 1.176022 -0.138138 3.547545

H 0.553061 -0.073591 4.450624

H 0.517896 -0.137735 2.672021

H 1.826849 0.745610 3.507089

C 2.309927 0.390228 -1.661987

C 3.847678 -0.269263 -3.151146

H 4.784602 -0.687642 -3.501694

C 2.822839 0.267045 -3.855675

H 2.708331 0.383711 -4.925854

C 0.619193 1.368626 -3.279513

C -0.561586 0.461453 -2.928860

H -1.507015 0.950220 -3.213301

H -0.591159 0.235397 -1.850913

H -0.483488 -0.485890 -3.483730

C 0.582600 1.671540 -4.781172

H -0.346989 2.214677 -5.002138

H 0.582322 0.755306 -5.390169

H 1.423156 2.308850 -5.093205

C 0.574884 2.692528 -2.516545

H -0.312032 3.274769 -2.804966

H 1.471715 3.288097 -2.741348

H 0.539504 2.532921 -1.432485

C 5.725492 -1.487174 -1.339422

C 7.074853 -1.133955 -1.469722

H 7.406300 -0.152819 -1.120134

C 8.012951 -2.001111 -2.037783

H 9.057508 -1.690242 -2.122935

C 7.620848 -3.256335 -2.494835

H 8.351619 -3.937113 -2.938644

C 6.281647 -3.635361 -2.380548

H 5.957650 -4.617194 -2.735751

C 5.358637 -2.760227 -1.813983

H 4.314219 -3.074522 -1.733752

C 5.265606 0.923606 -0.211235

C 5.985383 0.989079 0.993974

H 6.043171 0.100902 1.631236

C 6.625989 2.154396 1.409215

H 7.174209 2.168975 2.354882

C 6.559574 3.305774 0.623463

H 7.053638 4.225279 0.946974

C 5.852454 3.269658 -0.575704

H 5.786549 4.165405 -1.198883

C 5.221945 2.091958 -0.982988

H 4.674462 2.091036 -1.930621

C -3.709210 3.932834 1.377409

H -4.326674 4.389675 2.161310

C -3.604256 2.970283 -0.676780

H -4.125246 2.645866 -1.587900

C -2.237034 2.756857 -0.551188

H -1.731349 2.242923 -1.369740

C -1.017005 -1.290205 0.914044

C -0.651675 -2.538841 0.311702

C -1.215063 -3.729873 0.764884

H -0.923202 -4.666641 0.280568

C -2.138492 -3.765521 1.808311

H -2.564035 -4.711136 2.150627

C -2.473921 -2.563475 2.425618

H -3.165866 -2.581989 3.272583

C -1.930149 -1.343193 2.022935

C 0.324347 -2.591281 -0.851268

H 0.685226 -1.570405 -1.028064

C -0.361026 -3.054900 -2.140909

H 0.330790 -2.994541 -2.996629

H -1.237948 -2.431212 -2.369915

H -0.705452 -4.099252 -2.059753

C 1.545097 -3.461164 -0.544971

H 2.282172 -3.385775 -1.358548

H 1.270449 -4.523646 -0.440676

H 2.036898 -3.147705 0.385692

C -2.309691 -0.078503 2.781066

H -1.497010 0.652555 2.643924

C -3.575733 0.559989 2.207242

H -3.844082 1.477995 2.752428

H -4.409875 -0.158669 2.267346

H -3.413110 0.828399 1.152727

C -2.461267 -0.287090 4.290290

H -2.533767 0.683716 4.804808

H -1.604134 -0.835577 4.706688

H -3.372429 -0.854457 4.539838

B 4.600752 -0.482017 -0.697840

C -1.522158 3.150439 0.595802

H -1.017429 0.680768 0.791389

O -7.497201 0.546475 -1.975591

O -7.651073 -1.001979 0.430480

O -5.038043 -1.470625 0.073881

O -4.672696 0.128069 -2.066604

C -8.514158 -0.387955 -1.700449

H -8.924743 -0.807678 -2.636581

H -9.323770 0.164452 -1.197934

C -8.069622 -1.528555 -0.801953

H -8.918820 -2.226330 -0.662575

H -7.255673 -2.089809 -1.291210

C -7.005775 -1.902764 1.303705

H -7.686639 -2.718676 1.611163

H -6.767804 -1.315560 2.203303

C -5.730747 -2.498527 0.716111

H -5.126412 -2.961048 1.518029

H -5.968252 -3.303849 -0.007234

C -4.044834 -1.885229 -0.833287

H -4.467198 -2.660692 -1.504172

H -3.177588 -2.331111 -0.320321

C -3.590963 -0.666037 -1.614729

H -2.974973 -0.012978 -0.979257

H -2.955657 -0.987176 -2.458623

C -5.511141 -0.448305 -3.026928

H -5.797508 -1.478243 -2.748063

H -5.005398 -0.508034 -4.010881

C -6.753852 0.413189 -3.167956

H -6.449171 1.431580 -3.454197

H -7.388337 0.010716 -3.978355

K -5.936900 1.184699 0.158470

C -2.332891 3.782027 1.556424

H -1.897565 4.149061 2.494710

N -4.361223 3.519609 0.283884

N 1.031471 2.386344 1.263851

H 0.753425 2.078536 2.199518

C 1.954850 3.520661 1.462920

H -0.086481 2.788312 0.871436

C 2.329048 4.088674 0.094111

H 2.785622 3.308315 -0.531506

H 1.436654 4.477224 -0.419632

H 3.056639 4.908253 0.197265

C 3.217448 3.050068 2.190678

H 3.713199 2.243601 1.632414

H 3.941885 3.870318 2.312889

H 2.969676 2.668974 3.195748

C 1.264111 4.614159 2.295112

H 0.365057 4.987488 1.782371

H 0.957767 4.218131 3.278211

H 1.940809 5.464922 2.473102

**Pyridine**

Atom X Y Z

C -1.139841 -0.723003 -0.000221

C -1.197103 0.670781 -0.000117

C 0.000062 1.382625 0.000096

C 1.197163 0.670683 0.000218

C 1.139776 -0.723094 0.000130

N -0.000062 -1.411984 -0.000104

H 0.000083 2.475004 0.000143

H -2.063754 -1.311820 -0.000380

H -2.161156 1.182486 -0.000219

H 2.161284 1.182264 0.000366

H 2.063631 -1.311998 0.000182

***^t^*BuNH_2_**

Atom X Y Z

N -0.000459 0.182026 1.473258

H 0.813573 0.727544 1.754048

H -0.814976 0.727070 1.753565

C 0.000006 -0.004965 0.020375

C 0.000357 1.331864 -0.741256

H 0.000585 1.182697 -1.832781

H -0.890609 1.926925 -0.482523

H 0.891386 1.926657 -0.482135

C -1.253271 -0.806351 -0.342913

H -1.292879 -1.015028 -1.422808

H -1.267088 -1.760575 0.203471

H -2.165888 -0.248488 -0.075863

C 1.253381 -0.806563 -0.342133

H 1.266675 -1.760790 0.204253

H 1.293673 -1.015229 -1.422009

H 2.165922 -0.248874 -0.074461

**References**

1. Y. Gao, M. Pink, J. M. Smith, Alkali Metal Ions Dictate the Structure and Reactivity of an Iron(II) Imido Complex. *J. Am. Chem. Soc.* **144**, (2022): 1786.
2. J. Wang, A. K. Dash, M. Kapon, J. C. Berthet, M. Ephritikhine, M. S. Eisen, Oligomerization and Hydroamination of Terminal Alkynes Promoted by the Cationic Organoactinide Compound [(Et_2_N)_3_U][BPh_4_]. *Chem. Eur. J.* **8**, (2002): 5384.
3. D. F. Evans, The Determination of the Paramagnetic Susceptibility of Substances in Solutionby Nuclear Magnetic Resonance. *J. Chem. Soc.* (1959): 2003.
4. H. F. Yang, C. Zarate, W. N. Palmer, N. Rivera, D. Hesk, P. J. Chirik, Site-Selective Nickel-Catalyzed Hydrogen Isotope Exchange in *N*-Heterocycles and Its Application to the Tritiation of Pharmaceuticals. *ACS Catal.* **8**, (2018): 10210.
5. C. Yao, C. Copéret, Site-Selective and Late-Stage Deuteration of (Hetero)arenes with Supported Iridium Nanoparticles. *ACS Catal.*, **15**, (2025): 2822.
6. Y. Li, C. Zheng, Z. J. Jiang, J. Tang, B. Tang, Z. Gao, Potassium *Tert*-Butoxide Promoted Regioselective Deuteration of Pyridines. *Chem. Commun.* 58, (2022): 3497.
7. SAINT V8.40A (2020), Bruker AXS, Madison, WI.
8. L. Krause, R. Herbst-Irmer, G. M. Sheldrick, D. Stalke. Comparison of Silver and Molybdenum Microfocus X-ray Sources for Single-Crystal Structure Determination. *J. Appl. Cryst.* **48**, (2015): 3.
9. Gaussian 16, Revision A.03, M. J. Frisch, G. W. Trucks, H. B. Schlegel, G. E. Scuseria, M. A. Robb, J. R. Cheeseman, G. Scalmani, V. Barone, G. A. Petersson, H. Nakatsuji, X. Li, M. Caricato, A. V. Marenich, J. Bloino, B. G. Janesko, R. Gomperts, B. Mennucci, H. P. Hratchian, J. V. Ortiz, A. F. Izmaylov, J. L. Sonnenberg, D. Williams-Young, F. Ding, F. Lipparini, F. Egidi, J. Goings, B. Peng, A. Petrone, T. Henderson, D. Ranasinghe, V. G. Zakrzewski, J. Gao, N. Rega, G. Zheng, W. Liang, M. Hada, M. Ehara, K. Toyota, R. Fukuda, J. Hasegawa, M. Ishida, T. Nakajima, Y. Honda, O. Kitao, H. Nakai, T. Vreven, K. Throssell, J. A. Montgomery, Jr., J. E. Peralta, F. Ogliaro, M. J. Bearpark, J. J. Heyd, E. N. Brothers, K. N. Kudin, V. N. Staroverov, T. A. Keith, R. Kobayashi, J. Normand, K. Raghavachari, A. P. Rendell, J. C. Burant, S. S. Iyengar, J. Tomasi, M. Cossi, J. M. Millam, M. Klene, C. Adamo, R. Cammi, J. W. Ochterski, R. L. Martin, K. Morokuma, O. Farkas, J. B. Foresman, and D. J. Fox, Gaussian, Inc., Wallingford CT, 2016.
10. J. D. Chai, M. Head-Gordon, Long-range corrected hybrid density functionals with damped atom–atom dispersion corrections. *Phys. Chem. Chem. Phys.* **10**, (2008): 6615.
11. Yang, K; Zheng, J; Zhao, Y; Truhlar. D. G. Tests of the RPBE, revPBE, τHCTHhyb, ωB97X-D, and MOHLYP Density Functional Approximations and 29 Others Against Representative Databases for Diverse Bond Energies and Barrier Heights in Catalysis. *J. Chem. Phys*. **2010**, *132*, 164117
12. Li, R; Peverati, R.; Isegawa, M.; Truhlar, D. G. Assessment and Validation of Density Functional Approximations for Iron Carbide and Iron Carbide Cation. *J. Phys. Chem. A* **2013**, *117*, 169–173.
13. Hu, M. -Y; He, Q; Fan, S. -J; Wang. Z. -C; Liu, L. -Y; Mu, Y. -J; Peng, Q; Zhu, S. -F. Ligands with 1,10-phenanthroline scaffold for highly regioselective iron-catalyzed alkene hydrosilylation. *Nat. Commun*. **2018**, *9*, 221.
14. Zhang, H; Wang, E; Geng, S; Liu, Z; He, Y; Peng, Q; Feng, Z. Experimental and Computational Studies of the Iron-Catalyzed Selective and Controllable Defluorosilylation of Unactivated Aliphatic *gem*-Difluoroalkenes. *Angew. Chem. Int. Ed*. **2021**, *60*, 10211–10218.
15. Zhang, Y; Du, P; Ji, Y; Wang, S; Zhu, Y; Liu, Z; He, Y; Peng, Q; Feng, Z. Catalytic cross-electrophile coupling of aryl chlorides with unactivated alkyl chlorides: The synergy of iron and Li. *Chem*. **2023**, *9*, 3623–3636.
16. F. Weigend, R. Ahlrichs, Balanced basis sets of split valence, triple zeta valence and quadruple zeta valence quality for H to Rn: Design and assessment of accuracy. *Phys. Chem. Chem. Phys*. **7**, (2005): 3297.
17. F. Weigend, Accurate Coulomb-fitting basis sets for H to Rn. *Phys. Chem. Chem. Phys*. **8**, (2006): 1057.
18. K. Fukui, Formulation of the Reaction Coordinate. *J. Phys. Chem. A*. **74**, (1970): 4161.
19. K. Fukui, The Path of Chemical Reactions-The IRC Approach. *Acc. Chem. Res.* **14**, (1981): 363.
20. J. Zheng, X. Xu, D. G. Truhlar, Minimally augmented Karlsruhe basis sets. *Theoretical Chemistry Accounts.* **128**, (2011): 295.
21. A. V. Marenich, C. J. Cramer, D. G. Truhlar, Universal Solvation Model Based on Solute Electron Density and on a Continuum Model of the Solvent Defined by the Bulk Dielectric Constant and Atomic Surface Tensions. *J. Phys. Chem. B*. **113**, (2009): 6378.
22. T. Lu, Q. Chen, Independent Gradient Model based on Hirshfeld Partition: A New Method for Visual Study of Interactions in Chemical Systems, *J. Comput. Chem*. **43**, (2022): 539.
23. T. Lu, Q. Chen, Interaction Region Indicator: A Simple Real Space Function Clearly Revealing Both Chemical Bonds and Weak Interactions. *Chem. Methods.* **1**, (2021): 231.
24. T. Lu, Q. Chen, Multiwfn: A Multifunctional Wavefunction Analyzer. *J. Comput. Chem*. **33**, (**2012):** 580.
25. W. Humphrey, A. Dalke, K. Schulten, `VMD: Visual Molecular Dynamics. *J. Molec. Graphics*. **14**, (1996): 33.
26. CYLview, 1.0b; C. Y. Legault, Université de Sherbrooke, 2009 (<http://www.cylview.org>).
27. Gao, Y.; Carta, V.; Pink, M.; Smith, J. M. Catalytic Carbodiimide Guanylation by a Nucleophilic, High Spin Iron(II) Imido Complex. *J. Am. Chem. Soc*. **2021**, 143, 5324−5329.
